# Monocyte-derived macrophages exhibit distinct and more restricted HIV-1 integration site repertoire than CD4^+^ T cells

**DOI:** 10.1038/srep24157

**Published:** 2016-04-12

**Authors:** Yik Lim Kok, Valentina Vongrad, Mohaned Shilaih, Francesca Di Giallonardo, Herbert Kuster, Roger Kouyos, Huldrych F. Günthard, Karin J. Metzner

**Affiliations:** 1 Division of Infectious Diseases and Hospital Epidemiology, University Hospital Zurich, Zurich, Switzerland; 2 Institute of Medical Virology, University of Zurich, Zurich, Switzerland; 3 Marie Bashir Institute for Infectious Diseases and Biosecurity, Charles Perkins Centre, School of Biological Sciences and Sydney Medical School, The University of Sydney, Sydney, Australia

## Abstract

The host genetic landscape surrounding integrated HIV-1 has an impact on the fate of the provirus. Studies analysing HIV-1 integration sites in macrophages are scarce. We studied HIV-1 integration site patterns in monocyte-derived macrophages (MDMs) and activated CD4^+^ T cells derived from seven antiretroviral therapy (ART)-treated HIV-1-infected individuals whose cells were infected *ex vivo* with autologous HIV-1 isolated during the acute phase of infection. A total of 1,484 unique HIV-1 integration sites were analysed. Their distribution in the human genome and genetic features, and the effects of HIV-1 integrase polymorphisms on the nucleotide selection specificity at these sites were indistinguishable between the two cell types, and among HIV-1 isolates. However, the repertoires of HIV-1-hosting gene clusters overlapped to a higher extent in MDMs than in CD4^+^ T cells. The frequencies of HIV-1 integration events in genes encoding HIV-1-interacting proteins were also different between the two cell types. Lastly, HIV-1-hosting genes linked to clonal expansion of latently HIV-1-infected CD4^+^ T cells were over-represented in gene hotspots identified in CD4^+^ T cells but not in those identified in MDMs. Taken together, the repertoire of genes targeted by HIV-1 in MDMs is distinct from and more restricted than that of CD4^+^ T cells.

The human immunodeficiency virus-1 (HIV-1) mainly infects cells that express CD4 and the co-receptor CCR5 or CXCR4, i.e. CD4^+^ T cells and macrophages^1^,^2^,^3^,^4^,^5^,^6^. The phenotypes as a result of an HIV-1 infection in macrophages differ considerably from those of CD4^+^ T cells. For instance, macrophages are more resistant to HIV-1 cytopathic effects^7^,^8^. Specialized macrophages are widely distributed in various tissues, including anatomical sanctuaries. Indeed, HIV-1 has been detected in microglial cells of the central nervous system^9^,^10^,^11^. The involvement of HIV-1-infected macrophages, especially in the central nervous system, leading to the pathogenesis of HIV-1-associated diseases has been shown in various studies (reviewed in^12^).

Although it is conceivable that the different cellular physiology of macrophages in contrast to that of CD4^+^ T cells may contribute to the observed difference in productivity of the HIV-1 provirus in these two cell types, a multitude of studies, both *in vivo* and *in vitro*, have shown that the host genetic landscape surrounding the provirus does have an impact on the subsequent fate of the provirus. Earlier integration site studies have established the host genetic requirements for HIV-1 integration: (i) HIV-1 favours integration into active transcription units in gene-dense regions but disfavours centromeric alphoid repeats and human endogenous retrovirus (HERV) elements^13^,^14^,^15^, (ii) Alu repetitive elements are over-represented at HIV-1 integration sites^14^,^15^,^16^,^17^, (iii) HIV-1 provirus is flanked by the duplicated weakly conserved palindromic sequence 5′–GT(A/T)AC–3′^13^,^15^,^17^ and (iv) HIV-1 does not have a preference in transcription orientation relative to the gene into which it is integrated^14^,^17^.

Studies of HIV-1 integration site patterns in macrophages are scarce. This is partly due to the number of HIV-1-infected macrophages that can be recovered from blood samples of HIV-1-infected individuals is too low for HIV-1 integration site analysis, and obtaining them from other tissues is too invasive. Hence, *ex vivo* infection of primary monocyte-derived macrophages (MDMs) is a practical solution. The study of Barr *et al*.^18^ examined HIV-1 integration site patterns in MDMs and compared them with those in CD4^+^ T cells obtained from other studies. They observed that HIV-1 integration events are found more often in long intergenic regions and, correspondingly, less often in gene-dense regions in MDMs compared to CD4^+^ T cells. In the study of Wellensiek *et al*.^19^, although they had examined HIV-1 integration site patterns in MDMs and CD4^+^ T cells, they focussed on the differences between adult and neonatal cells of each cell type rather than between cell types. To date, a systematic comparison of HIV-1 integration site patterns between MDMs and CD4^+^ T cells derived from the same experimental settings has never been done.

Here, we extended the characterization of HIV-1 integration site patterns in MDMs and CD4^+^ T cells in an experimental setting that mimics the *in vivo* situation. We used MDMs and CD4^+^ T cells derived from ART-treated HIV-1-infected individuals and infected these cells *ex vivo* with the individuals’ autologous HIV-1 isolated during the acute phase of their HIV-1 infection. We focussed on other aspects of HIV-1 integration site patterns, e.g. structural and functional gene clusters formed by HIV-1-hosting genes and the frequencies of HIV-1 integration events in certain gene subsets, and compared these features between the two cell types. We also examined the effects of two naturally occurring amino acid polymorphisms in the HIV-1 integrase on its nucleotide selection specificity at HIV-1 integration sites in primary cells. We showed that the repertoire of genes targeted by HIV-1 in MDMs was distinct and more restricted than that of CD4^+^ T cells.

## Results

### Characteristics of study participants and their autologous primary HIV-1 isolates

At the time of autologous HIV-1 isolation, the study participants were infected with HIV-1 for an estimated 15–153 days (Supplementary Table S1). All HIV-1-infected individuals are enrolled in the Zurich Primary HIV Infection (ZPHI) study^20^ and six of seven HIV-1-infected individuals started early ART during the acute phase of infection. The replication capacities of these primary HIV-1 isolates in MDMs were most comparable to that of HIV-1_JR-FL_, a macrophage-tropic strain. The replication capacity of primary HIV-1 isolate 6 in MDMs was consistently the highest among the seven primary HIV-1 isolates (Supplementary Figure S1). All primary HIV-1 isolates were also replication competent in CD4^+^ T cells (Supplementary Figure S2).

At the time of MDMs and CD4^+^ T cell isolation from HIV-1-infected individuals, viral suppression ranged from 2.8–7.8 years (mean = 4.7 yr) (Supplementary Table S1). These cells were then infected *ex vivo* with autologous primary HIV-1 isolates or heterologous HIV-1 isolates. The non-restrictive linear amplification-mediated polymerase chain reaction (nrLAM-PCR)^21^ was used to amplify the 5′ HIV-1 integration junctions, which were analysed using our new Integration Site Analysis Pipeline (InStAP) (Supplementary Figure S3). Prior to these experiments, CD4^+^ T cells from three ART-treated HIV-1-infected individuals were activated and monitored for HIV-1 outgrowth. Reactivation of HIV-1 from latently infected cells did not occur within 7–8 days (data not shown). Thus, we are certain that the HIV-1 integration sites amplified from CD4^+^ T cells after two days of activation and a further two days of *ex vivo* infection were the result of these *ex vivo* infections. More importantly, no HIV-1 integration sites were found in mock infected cell samples of HIV-1-infected individuals.

### The basic genetic requirements for HIV-1 integration were similar among individual HIV-1 isolates and between MDMs and CD4^+^ T cells

**Figure 1:**
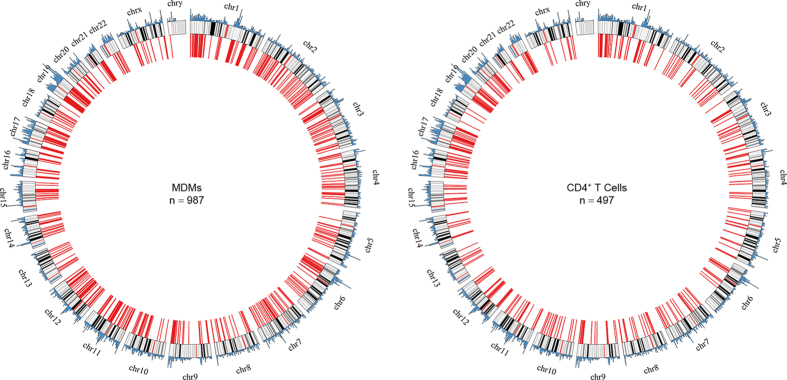
Chromosomal distribution of HIV-1 integration sites in monocyte-derived macrophages and CD4^+^ T cells infected *ex vivo*. Inner red lines indicate the position at which HIV-1 was found and outer blue peaks indicate gene densities. In each chromosome, red bands indicate centromeres, darker bands are AT-rich, and lighter bands are GC-rich.

We obtained a total of 1,484 unique HIV-1 integration sites, derived from the seven selected HIV-1-infected individuals whose MDMs (n = 987) and CD4^+^ T cells (n = 497) were infected *ex vivo* with autologous primary HIV-1 isolates and heterologous HIV-1 isolates (Supplementary Table S2). Of these, 783 (79.3%) and 431 (86.7%) integration sites were found within genes in MDMs and CD4^+^ T cells, respectively. HIV-1 integration sites were also found mainly in gene-dense regions and away from centromeres, Giemsa dark regions, and the p arms of acrocentric chromosomes in both MDMs and CD4^+^ T cells (Fig. 1). The genic distribution and genetic profiles of HIV-1 integration sites were consistent with previous studies^14^,^18^,^22^, and they were indistinguishable between MDMs and CD4^+^ T cells, as well as between pooled autologous and heterologous HIV-1 isolates (Supplementary Figure S4). These basic genetic requirements for HIV-1 integration were also similar among individual HIV-1 isolates (Supplementary Figure S5).

### Amino acid residue polymorphisms in HIV-1 integrase altered its nucleotide selection specificity in primary cells but not the genetic patterns of HIV-1 integration sites

Recent reports have identified the amino acid residues in the HIV-1 integrase that dictate the nucleotide selection specificity at integration sites, i.e. S119, T122, R231, and K258, in cell lines^23^,^24^. Since the full-length genomes of all well characterized primary HIV-1 isolates were sequenced, we could analyse these amino acid residues of our seven primary HIV-1 isolates and two clonal HIV-1 strains. Six HIV-1 isolates had the most common amino acid residues in all four positions. Primary HIV-1 isolates 2 and 6 exhibited a polymorphism at positions 122 (T122I) and 119 (S119P), respectively. These two polymorphisms were present concurrently in primary HIV-1 isolate 5 (Supplementary Figure S6). We investigated the effects of S119P and T122I polymorphisms on the nucleotide selection specificity at HIV-1 integration sites independently by analysing the 20 nucleotide positions immediately upstream of HIV-1 integration junctions (nucleotide position 0) in both MDMs and CD4^+^ T cells.

**Figure 2:**
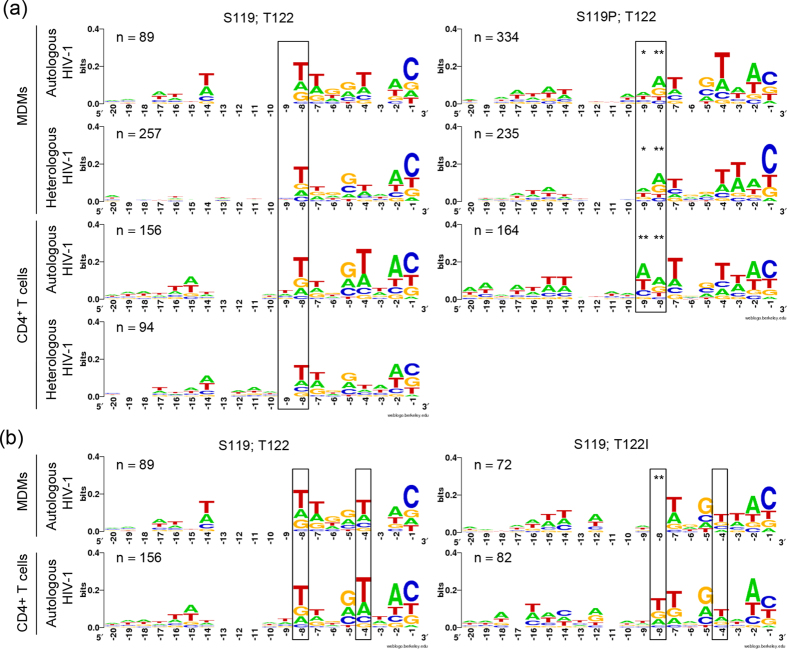
Conservation and frequency plots of 20 host nucleotides upstream of integrated HIV-1. (**a**) Left panel shows the nucleotide selection specificity of HIV-1 with a serine residue at position 119 of the HIV-1 integrase protein in a T122 background. Right panel shows the nucleotide selection specificity of HIV-1 with serine substituted by a proline residue at position 119 of the HIV-1 integrase protein in a T122 background. (**b**) Left panel shows the nucleotide selection specificity of HIV-1 with a threonine residue at position 122 of the HIV-1 integrase protein in an S119 background. Right panel shows the nucleotide selection specificity of HIV-1 with threonine substituted by an isoleucine residue at position 122 of the HIV-1 integrase protein in an S119 background. χ^2^ test: *p < 0.01 and **p < 0.0001 compared to the same nucleotide positions in the left panel.

We analysed two nucleotide positions that were previously reported to be altered by the S119P HIV-1 integrase variant^23^. S119P resulted in a significantly increased preference for adenines at positions −8 (p < 0.0001) and −9 (p < 0.01) in both MDMs and CD4^+^ T cells (Fig. 2a), consistent with previous report^23^. T122I significantly decreased the preference of HIV-1 for thymines at position −8 in MDMs (p < 0.0001) (Fig. 2b), similar to the previously reported T122K^23^. Nonetheless, a consistent decrease in preference of HIV-1 for thymines at positions −4 and −8 was observed in both MDMs and CD4^+^ T cells (Fig. 2b). Primary HIV-1 isolate 5, which exhibited both polymorphisms in its integrase, could not be analysed due to the lack of sufficient HIV-1 integration sites. Both polymorphisms (S119P and T122I) dictated the nucleotide selection specificity at HIV-1 integration sites in primary cells.

### The repertoire of HIV-1-hosting genes in MDMs was more restricted and distinct from that of CD4^+^ T cells

Next, we assessed whether the annotated features of HIV-1-hosting genes, e.g. involvement in cellular and molecular processes, were different between the two cell types using the Database for Annotation, Visualization and Integrated Discovery (DAVID)^25^,^26^. The majority of HIV-1-hosting gene clusters over-represented in the human genome had annotations associated with structural motifs and domains of DNA and proteins in both cell types (Supplementary Figure S7). However, these gene clusters were more diverse in CD4^+^ T cells, which was also the case in most other categories of gene clusters (data not shown). MDMs had more over-represented gene clusters that were associated with protein metabolism, regulation, and transport whereas the proportion of over-represented gene clusters associated with a variety of other cellular processes, e.g. regulation of apoptosis, was higher in CD4^+^ T cells (Supplementary Figure S7). There were also gene clusters that were observed exclusively in either cell type, e.g. deubiquitination and regulation of axonogenesis in MDMs whereas T cell receptor signalling and cell cycle progression in CD4^+^ T cells (data not shown), which concordantly reflected the cellular and molecular functions of the corresponding cell types.

As the diversity of structural and functional HIV-1-hosting gene clusters appeared to be higher in CD4^+^ T cells, we investigated further the extent to which these gene clusters overlap between cell types in order to estimate the relative size of the repertoire of genes accessible to HIV-1 for integration. For this purpose, we analysed the top 40 gene clusters of each data set, including both over-represented and not over-represented gene clusters in the human genome. We scored gene clusters having the same or highly similar annotations between data sets, and observed that the gene cluster overlap was the highest (47.5%) between MDM/HIV-1_autologous_ and MDM/HIV-1_heterologous_, and decreased by 7.5–15% between MDMs and CD4^+^ T cells. As expected, between CD4^+^ T cell/HIV-1_autologous_ and CD4^+^ T cell/HIV-1_heterologous_, the extent of overlap was lower (32.5%) (Table 1). This apparent trend of gene cluster overlaps was reproducible when we included HIV-1 integration site data sets from two other studies: the one other publicly available MDM data set (n = 754)^18^ and another CD4^+^ T cell data set (n = 534)^27^ (Table 1). Furthermore, to confirm the quality and comparability among data sets, we used a human T-lymphotropic virus-1 (HTLV-1) integration site data set from the study of Meekings *et al*.^28^ as a denominator control (Table 1). All six HIV-1 integration site data sets overlapped to similarly low extents with that of HTLV-1.

Due to the unequal number of HIV-1 integration sites recovered from each HIV-1-infected individual’s sample infected *ex vivo*, the observed results could have been driven mainly by samples of two individuals with the highest number of recovered HIV-1 integration sites, i.e. individuals 1 and 6 (Supplementary Table S2). To address this issue, we performed two analyses: (i) the proportion of HIV-1 integration sites forming the top 40 gene clusters were compared with the proportion of HIV-1 integration sites recovered from each HIV-1-infected individual’s sample and (ii) gene clusters formed with data from individuals 1 or 6 only, and when removed, were compared with the entire corresponding data sets. A high correlation (R^2^ = 0.9774) was observed between the proportion of HIV-1 integration sites forming the top 40 gene clusters and that of HIV-1 integration sites recovered from each HIV-1-infected individual’s sample, indicating that each individual’s sample contributed towards the formation of the top 40 gene clusters, and the extent of their contributions were concordant with the proportion of HIV-1 integration sites recovered (Supplementary Figure S8). Additionally, data sets containing only HIV-1 integration sites derived from HIV-1-infected individuals 1 or 6 versus data sets without these HIV-1 integration sites overlapped with the entire corresponding data set to comparable levels (Supplementary Table S3), demonstrating our observations were neither HIV-1-infected individual-specific nor skewed by these two individuals who had the highest number of recovered HIV-1 integration sites.

**Figure 3:**
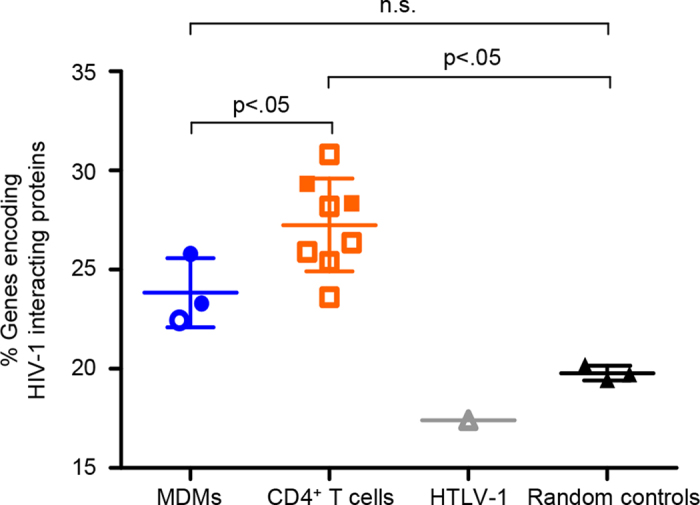
Frequency of HIV-1 integration events in genes encoding HIV-1-interacting proteins. A total of three MDM data sets (two derived from this study; closed circles) and eight CD4^+^ T cell data sets (two derived from this study; closed squares) were compared to three sets of 5,000 random genes generated *in silico*. Details of data sets derived from other studies (open symbols) are found in Table 2. HTLV-1 integration site data set served as an independent control. Two-tailed Mann-Whitney U test with 95% confidence interval was used to test for statistical significance.

Using the National Centre for Biotechnology Information (NCBI) database of published protein interactions between HIV-1 and its cellular host^29^, we investigated the frequency of HIV-1 integration events in a subset of genes encoding HIV-1-interacting proteins. We hypothesized that this frequency would be different between MDMs and CD4^+^ T cells given the lower HIV-1-hosting gene cluster overlap between them (Table 1). Here, we expanded our analysis and included a total of 11 HIV-1 integration site data sets: two MDM data sets (n = 987) and two CD4^+^ T cell data sets (n = 497) derived from this study and one other MDM data set (n = 754) and six other CD4^+^ T cell data sets (n = 7,557) obtained elsewhere^14^,^18^,^27^,^30^,^31^,^32^,^33^ (Table 2). We observed an increase of HIV-1 integration events in genes encoding HIV-1-interacting proteins in MDMs (1.2×) and a significant increase in CD4^+^ T cells (1.4×, p < 0.001) compared to three sets of 5,000 random genes generated *in silico* (Fig. 3). As the CD4^+^ T cell data sets were derived from both activated and resting states, we further tested if the difference in cellular activation states in CD4^+^ T cells would have an influence on the frequency of HIV-1 integration into genes encoding HIV-1-interacting proteins; no significant difference was observed (data not shown). Though quantitatively modest, there was a significant difference (p < 0.05) between MDMs and CD4^+^ T cells (Fig. 3).

Given the consistent trend of gene cluster overlaps, a rigorous assessment to show the robustness of our analysis, and a significantly different (p < 0.05) over-representation of HIV-1 integration events in genes encoding HIV-1-interacting proteins between MDMs and CD4^+^ T cells, we demonstrated that the repertoires of genes accessible to HIV-1 for integration in MDMs and CD4^+^ T cells were distinct and that of MDMs’ was more restricted.

### Genes linked to clonal expansion of latently HIV-1-infected CD4^+^ T cells were targeted more frequently in CD4^+^ T cells but not monocyte-derived macrophages

Expansion of the viral reservoir by means of clonal expansion of latently HIV-1-infected CD4^+^ T cells harbouring HIV-1 proviruses in genes of certain functional nature has recently been suggested^27^,^30^,^32^. These genes are referred to as genes linked to clonal expansion of latently HIV-1-infected CD4^+^ T cells here.

**Figure 4:**
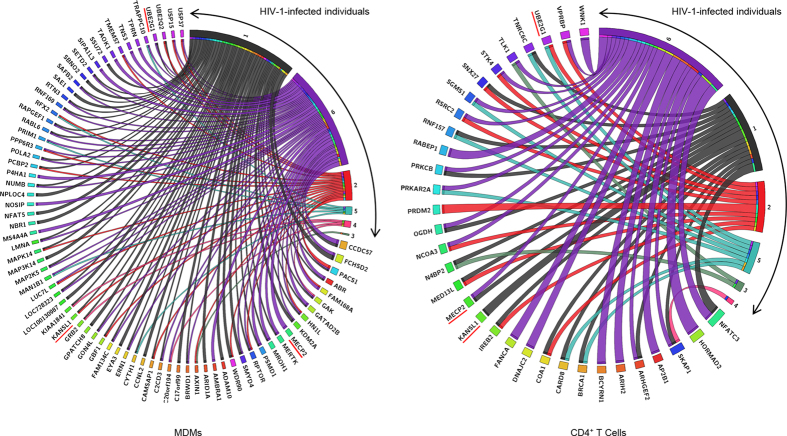
Gene hotspots for HIV-1 integration identified in MDMs and CD4^+^ T cells. Genes in which at least two independent HIV-1 integration events were identified in MDMs (n = 75) and CD4^+^ T cells (n = 33) are shown along with the HIV-1-infected individuals’ samples from which these HIV-1-hosting gene hotspots were identified. HIV-1-infected individuals’ samples are colour-coded and strand widths are directly proportional to the number of independent integration sites. Underlined in red are gene hotspots that were identified at least four times in both cell types.

**Figure 5:**
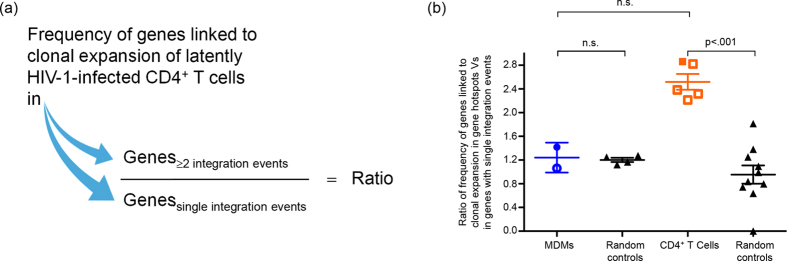
Frequency of HIV-1 integration events in genes linked to clonal expansion of latently HIV-1-infected CD4^+^ T cells. (**a**) Each HIV-1 integration site data set was first divided into two groups: one comprises genes in which two or more HIV-1 integration events were identified (gene hotspots) and the other comprises genes in which only single HIV-1 integration events were identified. The frequency of genes linked to clonal expansion of latently HIV-1-infected CD4^+^ T cells in the two groups of genes for each data set was determined and the ratio was computed. (**b**) Ratio of frequency of genes linked to clonal expansion of latently HIV-1-infected CD4^+^ T cells in gene hotspots to in genes with single HIV-1 integration events. Autologous and heterologous HIV-1 data sets were combined here (closed symbols). Details of data sets derived from other studies (open symbols) are found in Table 2. Each random control comprised 5,000 random genes generated *in silico* instead of genes linked to clonal expansion of latently HIV-1-infected CD4^+^ T cells. Two random controls were analysed for each data set. Two-tailed Mann-Whitney U test with 95% confidence interval was used to test for statistical significance.

We compiled genes linked to clonal expansion of latently HIV-1-infected CD4^+^ T cells identified in two studies^27^,^30^ and collapsed them into a total of 196 unique genes. Next, we divided each HIV-1 integration site data set into two groups; one containing unique protein-coding genes in which HIV-1 was found ≥2 times, i.e. gene hotspots (Fig. 4) and the other, only once (Fig. 5a). We included seven HIV-1 integration site data sets for this analysis: two derived from this study (data sets of the same cell type combined; n = 1,484) and one other MDM data set (n = 754) and four other CD4^+^ T cell data sets (n = 4,613) obtained elsewhere^14^,^18^,^31^,^32^,^33^ (Table 2),

By comparing the frequencies of HIV-1-hosting genes linked to clonal expansion of latently HIV-1-infected CD4^+^ T cells in gene hotspots and genes with single HIV-1 integration events, we observed a highly significant increase of genes linked to clonal expansion of latently HIV-1-infected CD4^+^ T cells in gene hotspots in CD4^+^ T cells (2.5×, p < 0.001), but not in MDMs (Fig. 5b), showing again that the repertoires of genes accessible to HIV-1 for integration in the two cell types were distinct.

### Gene hotspots identified for HIV-1 integration

We identified a total of 75 (9.5%) and 33 (7.7%) genes in which HIV-1 was found ≥2 times in MDMs and CD4^+^ T cells, respectively (Fig. 4). Furthermore, ~70% of these genes were identified in ≥2 HIV-1-infected individuals. As the probability of getting the same gene ≥2 times in multiple data sets was low^34^, these genes were considered as hotspots for HIV-1 integration here. Three gene hotspots were identified ≥4 times in both cell types, i.e. *MECP2*, *KANSL1*, and *UBE2G1*, and they were also listed in other studies (Table 3). In most cases, HIV-1 proviruses integrated into the same introns (Table 3), and they spanned 36 kb, 139 kb, and 22 kb in *MECP2*, *KANSL1*, and *UBE2G1*, respectively.

## Discussion

We compared HIV-1 integration sites patterns between macrophages and CD4^+^ T cells derived from seven ART-treated HIV-1-infected individuals whose cells were infected *ex vivo* with autologous, as well as heterologous, primary HIV-1 isolates generated during the acute phase of infection. This unique experimental set-up allowed us to replicate the *in vivo* situation for the examination of HIV-1 integration site patterns arising from the two major cellular targets of HIV-1 and potentially among different HIV-1 isolates.

The various genetic features at HIV-1 integration sites were similar among different HIV-1 isolates, regardless of whether the virus was autologous or heterologous. However, given that the gene clusters consistently overlapped to a higher extent in MDMs, the repertoire of genes accessible to HIV-1 for integration in MDMs appeared to be more restricted than and distinct from that of CD4^+^ T cells. This distinction between the two cell types was again reflected in the different frequencies of HIV-1-hosting genes in two gene subsets: genes encoding HIV-1-interacting proteins and those linked to clonal expansion of latently HIV-1-infected CD4^+^ T cells. Firstly, we observed an over-representation of HIV-1 integration events in the former gene subset in both MDMs and CD4^+^ T cells, but to different extents. Despite the heterogeneity of the NCBI HIV-1 Human Interaction Database^29^, its representativeness and specificity were demonstrated by the substantially lower frequency of HIV-1-interacting genes in random and HTLV-1 data sets. Secondly, HIV-1 integration into genes linked to clonal expansion of latently HIV-1-infected CD4^+^ T cells was over-represented in gene hotspots in CD4^+^ T cells but not in MDMs.

The gene cluster distribution of our CD4^+^ T cell data set was consistent with that derived from the study of Wagner *et al*.^27^, whereas such consistency was somewhat lacking between our MDM data sets with that derived from the study of Barr *et al*.^18^. Comparison of HIV-1 integration sites between MDMs and CD4^+^ T cells derived from the same experimental settings is important, as evident from the absolute percentages of the top 40 DAVID gene cluster overlaps; MDMs from the study of Barr *et al*.^18^ and CD4^+^ T cells from the study of Wagner *et al*.^27^ had the same absolute values when compared to MDMs from this study. Nonetheless, the trend of gene cluster overlaps was consistent across all data sets that were derived from the same experimental settings, i.e. gene cluster overlaps were the highest between MDM data sets, followed by between MDM and CD4^+^ T cell data sets, and the lowest was observed between CD4^+^ T cell data sets.

We focussed on gene hotspots that were identified at least four times in both cell types, i.e. *MECP2*, *KANSL1*, and *UBE2G1*. The products of these genes are involved in transcriptional and post-translational regulations of genes^35^,^36^,^37^. Interestingly, downregulation of MECP2 has been reported to enhance replication of HIV-1_NL4-3_ in Jurkat cells^38^. Although it was unclear if these gene hotspots, as well as genes encoding HIV-1-interacting proteins were highly accessible prior to or upon HIV-1 infection, it is conceivable that they were transcriptionally active enough to allow integration^14^,^15^,^18^,^31^ and/or located in the vicinity of the nuclear pore, close to the incoming HIV-1 DNA^34^. The model of Marini *et al*.^34^ describes that specific chromatin organization in the nucleus results in certain genes being closer to the nuclear pore, thus recurrently targeted by HIV-1 for integration. This model could be envisaged to be applicable down to the levels of genes, in which certain intragenic regions might be closer and more exposed to the incoming HIV-1 DNA. This would explain why HIV-1 proviruses that were found in the three gene hotspots preferred integration into the same introns in most cases.

The effect of T122I polymorphism on HIV-1 nucleotide selection specificity has not been reported previously. This natural and the second most abundant amino acid polymorphism at position 122 consistently altered the nucleotide selection specificity of HIV-1, demonstrating its role in integration site targeting. The effect of S119P polymorphism on HIV-1 nucleotide selection at the site of integration in cell lines has been described by Demeulemeester *et al*.^23^, and our observation in primary cells was consistent with theirs. Certain amino acid polymorphisms of the HIV-1 integrase, especially at position 119, have been associated with rapid disease progression^23^. However, association of natural integrase variants with disease progression could not be examined in our study participants because all of them have been successfully treated. Although these HIV-1 integrase polymorphisms altered the nucleotide selection specificity at integration sites, they did not have an effect on various genetic features at HIV-1 integration sites^23^. It is likely that the retargeting of HIV-1 was restricted by the repertoire of accessible genes in MDMs and CD4^+^ T cells.

In summary, we demonstrated in various analyses using our own data sets and of others that the repertoires of HIV-1-hosting genes targeted by HIV-1 in MDMs and CD4^+^ T cells were distinct in terms of their structural and functional classes and relative sizes. These cell type-dependent HIV-1 integration site patterns might account for the differences in the life cycle of HIV-1 in these cell populations.

## Methods

### HIV-1-infected study participants and HIV-1 negative donors

HIV-1-infected individuals are enrolled in the Zurich Primary HIV Infection (ZPHI) study, which is a non-randomized, observational, open-label, and single-centre study (www.clinicaltrials.gov; ID: NCT00537966; registered 25^th^ September 2007)^20^. For the current sub-study, the inclusion criteria were: (i) HIV-1-infected individuals had at least 1.5 years of viral suppression of <20 HIV-1 RNA copies/mL plasma with antiretroviral therapy (ART) at the time of cell sample collection, and (ii) their primary HIV-1 isolates were capable of replication in monocyte-derived macrophages (MDMs).

### Ethics, consent, and permission

This study was approved by the Ethics Committee of the University Hospital Zurich and written informed consent was obtained from all HIV-1-infected individuals and HIV-1 negative donors. All experiments were performed in accordance with the relevant guidelines and regulations.

### Generation and sequencing of primary HIV-1 isolates and clonal HIV-1 strains

The generation of primary HIV-1 isolates has been described elsewhere^39^. Briefly, CD4^+^ T cells from HIV-1-infected individuals in the acute phase of infection were co-cultured with activated donors’ peripheral blood mononuclear cells (PBMCs) until the HIV-1 p24 levels of the culture supernatant was >2,000 pg/mL prior to harvesting of the viral supernatant. HIV-1 p24 levels were measured with an enzyme-linked immunosorbent assay (ELISA)^40^. The 50% tissue culture infectious dose (TCID_50_) of the primary HIV-1 isolates was determined by co-culturing quadruplicates of 5-fold dilutions of primary HIV-1 isolates and scoring the number of p24-positive wells, and the TCID_50_ was subsequently calculated using the method of Reed and Muench^41^.

HIV-1_JR-FL_^42^ and HIV-1_NL4-3_^43^ were generated by transfecting HEK 293T cells with pJR-FL and pNL4-3, respectively, and the viral supernatant was harvested and cleared off of cells with a 0.22 μm filter 48 hours post-transfection. pJR-FL^42^ was obtained from Irvin SY Chen and Yoshio Koyanagi, and pNL4-3^43^ was obtained from Malcolm Martin through the NIH AIDS Reagent Programme, Division of AIDS, NIAID, NIH.

We had previously developed a sensitive and robust method to amplify and sequence the full-length genomes of primary HIV-1 isolates with minimal artefactual *in vitro* recombination^44^. The seven selected primary HIV-1 isolates were all confirmed to be of subtype B as determined by their *pol* nucleotide sequences using the REGA HIV-1 subtyping tool V3^45^.

### *Ex vivo* HIV-1 infection of monocyte-derived macrophages and CD4^+^ T cells

Blood samples of ART-treated HIV-1-infected individuals were first depleted of CD8^+^ T cells using RosetteSep Human CD8 Depletion Cocktail (Stemcell Technologies). CD8^−^ PBMCs were isolated by density gradient centrifugation using Lymphoprep (Stemcell Technologies). CD14^+^ monocytes were positively isolated by magnetic-activated cell sorting using CD14 microbeads (Miltenyi Biotec) whereas the remaining CD8^−^CD14^−^ fraction was treated functionally as CD4^+^ T cells. The median purities of CD14^+^ monocytes and CD4^+^ T cells were 95.4% and 98.8%, respectively, as measured by flow cytometry. CD14^+^ cells were induced to differentiate into MDMs in RPMI-1640 (10% (v/v) fetal bovine serum (FBS), 100 U/mL Penicillin, and 100 μg/mL Streptomycin) supplemented with 5% (v/v) human serum (Sigma) and 20 ng/mL macrophage-colony stimulating factor (M-CSF) (Peprotech) for eight days. CD4^+^ T cells were activated in RPMI-1640 (10% (v/v) FBS, 100 U/mL Penicillin, and 100 μg/mL Streptomycin) supplemented with 10 U/mL IL-2 (Roche) and cultured in OKT3-coated flasks for two days. MDMs and activated CD4^+^ T cells were infected *ex vivo* with primary HIV-1 isolates or clonal strains (HIV-1_JR-FL_ or HIV-1_NL4-3_) at an MOI of 0.74–1.0 for six days and 0.01 for two days, respectively.

In the screening of macrophage-tropic primary HIV-1 isolates, HIV-1 negative donors’ blood samples were used and treated essentially the same way to obtain MDMs and CD4^+^ T cells, except that the isolated CD4^+^ T cells were divided into three fractions, activated individually with 5 U/ml phytohaemagglutinin (PHA), 0.5 U/mL PHA, and OKT3, and pooled two days later prior to HIV-1 infection *ex vivo*.

### Amplification and sequencing of 5′ HIV-1 integration junctions

The 5′ HIV-1 integration junctions were amplified with the non-restrictive linear amplification-mediated PCR (nrLAM-PCR)^21^ from a total of 0.2–1.0 μg of genomic DNA extracted from cells infected *ex vivo* with HIV-1 isolates. The procedure was essentially the same as described elsewhere^21^, except: (i) the 3′ primers used for linear, first exponential, and second exponential amplifications were 5′CCCTGGTGTGTAGTTCTGCC3′, 5′CAATCAGGGAAGTAGCCTTGTG3′, and 5′TCGTCGGCAGCGTCAGATGTGTATAAGAGACAGN_0–3_ TGTGGTAGACCCACAGATCAAG3′, respectively, (ii) the 5′ primer used for second exponential amplification was 5′GTCTCGTGGGCTCGGAGATGTGTATAAGAGACAGN_0–3_GATCTGAATTCAGTGGCACAG3′, (iii) the cycling conditions for linear amplification were 2× (95 °C for 2 min followed by 50 cycles of 95 °C for 45 s, 57 °C for 45 s, and 72 °C for 8 s) and (iv) The cycling conditions for first and second exponential amplifications were 95 °C for 2 min followed by 40 cycles of 95 °C for 45 s, 57 °C for 45 s, and 72 °C for 8 s, and a final extension at 72 °C for 2 min. The HIV-1 5′ LTR-specific primers were designed based on the most conserved regions of the consensus sequence of HIV-1 subtype B in the Los Alamos HIV Sequence Database. The N_0–3_ random nucleotides in the primers used for the second exponential amplification were introduced to increase nucleotide diversity to enable DNA cluster generation during DNA sequencing with the Illumina MiSeq platform.

5′ HIV-1 integration junctions were sequenced with the Illumina MiSeq platform. Illumina sequencing adaptors were added to 1 ng of purified PCR products in a PCR using the following primers: 5′AATGATACGGCGACCACCGAGATCTACAC(Index)TCGTCGGCAGCGTC3′ and 5′CAAGCAGAAGACGGCATACGAGAT(Index)GTCTCGTGGGCTCGG3′ at 0.5 μM each. The cycling conditions were 72 °C for 3 min, 95 °C for 30 s, 12 cycles of 95 °C for 10 s, 55 °C for 30 s, and 72 °C for 30 s, and a final extension at 72 °C for 5 min. A total of 12–14 pM of purified PCR products were sequenced with the MiSeq Reagent Kit v2 (300 or 500 cycles) with 8% PhiX.

### Post-sequencing processing and mapping to the human genome assembly

Sequencing reads were filtered to remove low quality reads. True 5′ HIV-1 integration junctions were identified using an in-house bioinformatic pipeline, i.e. Integration Site Analysis Pipeline (InStAP), and satisfied the following criteria: (i) presence of both the nrLAM-PCR adaptor and 5′ HIV-1 LTR, (ii) host DNA was within 3 nucleotides from the end of 5′ HIV-1 LTR, and (iii) ≥85% of the lengths of the reads mapped to the human assembly GRCh37.p13 with ≥98% identity. Contaminants were accounted for and removed from all samples in which they were identified except the sample that was sequenced first and/or with the highest number of read count. Manuscript on the details of InStAP is in preparation.

HIV-1 integration site data sets derived from this study and obtained elsewhere for comparison are shown in Table 2. All HIV-1 integration sites derived from this study are listed in Supplementary Table S4.

### Bioinformatic databases

Annotated features of HIV-1-hosting genes were assessed using the Database for Annotation, Visualization and Integrated Discovery (DAVID)^25^,^26^. DAVID groups genes, which are related by their structural and functional annotations, into clusters and calculates an enrichment score to indicate if the gene clusters were significantly over-represented in a particular genomic background. Clusters of HIV-1-hosting genes that were significantly over-represented in the human genome are defined as having a DAVID enrichment score >1.3 with the stringency level in DAVID set to high. By definition, a gene cluster having a DAVID enrichment score >1.3 was equivalent to an overall geometric mean <0.05 for all its gene members, and a high stringency would consider a gene cluster as biologically significant only when the kappa coefficient, κ ≥ 0.85 for at least three genes with at least three related annotations between any two.

The National Centre for Biotechnology Information (NCBI) catalogues and provides access to a database of published interactions between HIV-1 proteins and its cellular host^29^. We used the November 2014 release (the latest at time of analysis) of the HIV-1 Human Interaction Database, which documented a total of 3,337 unique RefSeq protein-coding genes whose products interact physically, as well as indirectly, with HIV-1. This number of genes represented ~16.15% of all unique RefSeq protein-coding genes in the human genome (20,664 as of 15^th^ of December 2014).

### Statistical analyses

Two-tailed Fisher’s exact test, χ^2^ test, and Mann-Whitney U test with a 95% confidence interval were used at where indicated.

### Graphical plots

Some of the graphical plots were generated using the following online platforms: Plotly (https://plot.ly/), WebLogo^46^,^47^, and Circos^48^.

## Additional Information

**How to cite this article**: Kok, Y. L. *et al*. Monocyte-derived macrophages exhibit distinct and more restricted HIV-1 integration site repertoire than CD4^+^ T cells. *Sci. Rep*. **6**, 24157; doi: 10.1038/srep24157 (2016).

## Supplementary Material

Supplementary Information

## Figures and Tables

**Table 1:** DAVID structural and functional gene clusters overlap between data sets.

Data set 1	Data set 2	Top 40 DAVID gene cluster overlap (%)
MDM/HIV-1_a_	MDM/HIV-1_h_	47.5
	CD4^+^ T cell/HIV-1_a_	32.5
	CD4^+^ T cell/HIV-1_h_	37.5
MDM/HIV-1_h_	CD4^+^ T cell/HIV-1_a_	37.5
	CD4^+^ T cell/HIV-1_h_	40.0
CD4^+^ T cell/HIV-1_a_	CD4^+^ T cell/HIV-1_h_	32.5
MDMs^18^	MDM/HIV-1_a_	37.5
	MDM/HIV-1_h_	37.5
	CD4^+^ T cell/HIV-1_a_	27.5
	CD4^+^ T cell/HIV-1_h_	22.5
CD4^+^ T cells^27^	MDM/HIV-1_a_	37.5
	MDM/HIV-1_h_	37.5
	CD4^+^ T cell/HIV-1_a_	32.5
	CD4^+^ T cell/HIV-1_h_	27.5
HTLV-1^28^	MDM/HIV-1_a_	22.5
	MDM/HIV-1_h_	20.0
	CD4^+^ T cell/HIV-1_a_	17.5
	CD4^+^ T cell/HIV-1_h_	20.0
	MDMs^18^	25.0
	CD4^+^ T cells^27^	25.0

HIV-1- and HTLV-1-hosting genes were clustered based on their structural and functional annotations using DAVID. The top 40 clusters from all data sets were compared and clusters having the same or highly similar annotations were scored.

MDM: Monocyte-derived macrophages; a: autologous; h: heterologous; HTLV-1: Human T-lymphotropic virus-1.

**Table 2:** Data sets examined in this study.

Cell type	Virus	Number of integration sites reported	Source
MDMs	HIV-1	987	This study
CD4^+^ T cells	HIV-1	497	This study
MDMs	HIV-1	754	18
CD4^+^ T cells	HIV-1	457	32
CD4^+^ T cells	HIV-1	2661	33
CD4^+^ T cells	HIV-1	2410	30
PBMCs	HIV-1	534	27
SupT1 cells	HIV-1	524	14
Jurkat cells	HIV-1	971	31
Jurkat cells and PBMCs	HTLV-1	579	28

**Table 3:** Gene hotspots for HIV-1 integration identified in both monocyte-derived macrophages and CD4^+^ T cells.

Gene	Cell type	HIV-1-infected individual	HIV-1	Intragenic region	Other studies
*MECP2, chr. X*	MDM	6	autologous	Intron 1/2* (−)	14, 32, 33
	MDM	6	autologous	Intron 1/2* (−)	
	MDM	6	heterologous	Intron 1/2* (−)	
	CD4^+^ T cell	6	autologous	Intron 1/2* (−)	
	CD4^+^ T cell	1	autologous	Intron 1/2* (−)	
*KANSL1, chr. 17*	MDM	1	heterologous	Intron 2/3* (+)	14, 31, 33
	MDM	2	heterologous	Intron 2/3* (+)	
	CD4^+^ T cell	1	autologous	Intron 2 (+)	
	CD4^+^ T cell	1	autologous	Intron 5/6* (+)	
*UBE2G1, chr. 17*	MDM	6	autologous	Intron 1 (−)	14, 31, 32, 33
	MDM	1	heterologous	Intron 1 (−)	
	CD4^+^ T cell	2	autologous	Intron 2 (−)	
	CD4^+^ T cell	5	heterologous	Intron 1 (+)	

Parentheses in the Intragenic region column indicate transcription orientation of HIV-1 proviruses relative to the genes into which they were integrated: + indicates same whereas − indicates convergent. *Transcript variants.
