# Supplementary material for: Monocyte-derived macrophages exhibit distinct and more restricted HIV-1 integration site repertoire than CD4+ T cells
Source: Sci Rep. 2016 Apr 12;6:24157. doi: 10.1038/srep24157 (PMC4828718; doi:10.1038/srep24157)
Supplement: Supplementary Information [file srep24157-s1.pdf]

Title

Monocyte-derived macrophages exhibit distinct and more restricted HIV-1 integration site repertoire than $CD4^+$ T cells

Authors

Yik Lim Kok[1,2,*], Valentina Vongrad[1,2,*], Mohaned Shilaih[1,2,*], Francesca Di Giallonardo[3], Herbert Kuster[1,2], Roger Kouyos[1,2], Huldrych F. Günthard[1,2], and Karin J. Metzner[1,2].

Affiliation

[1]Division of Infectious Diseases and Hospital Epidemiology, University Hospital Zurich, Zurich, Switzerland.

[2]Institute of Medical Virology, University of Zurich, Zurich, Switzerland.

[3]Marie Bashir Institute for Infectious Diseases and Biosecurity, Charles Perkins Centre, School of Biological Sciences and Sydney Medical School, The University of Sydney, Sydney, Australia.

Corresponding author: Karin J. Metzner

E-mail: karin.metzner@usz.ch

[*]These authors contributed equally to this work

Supplementary Figure S1

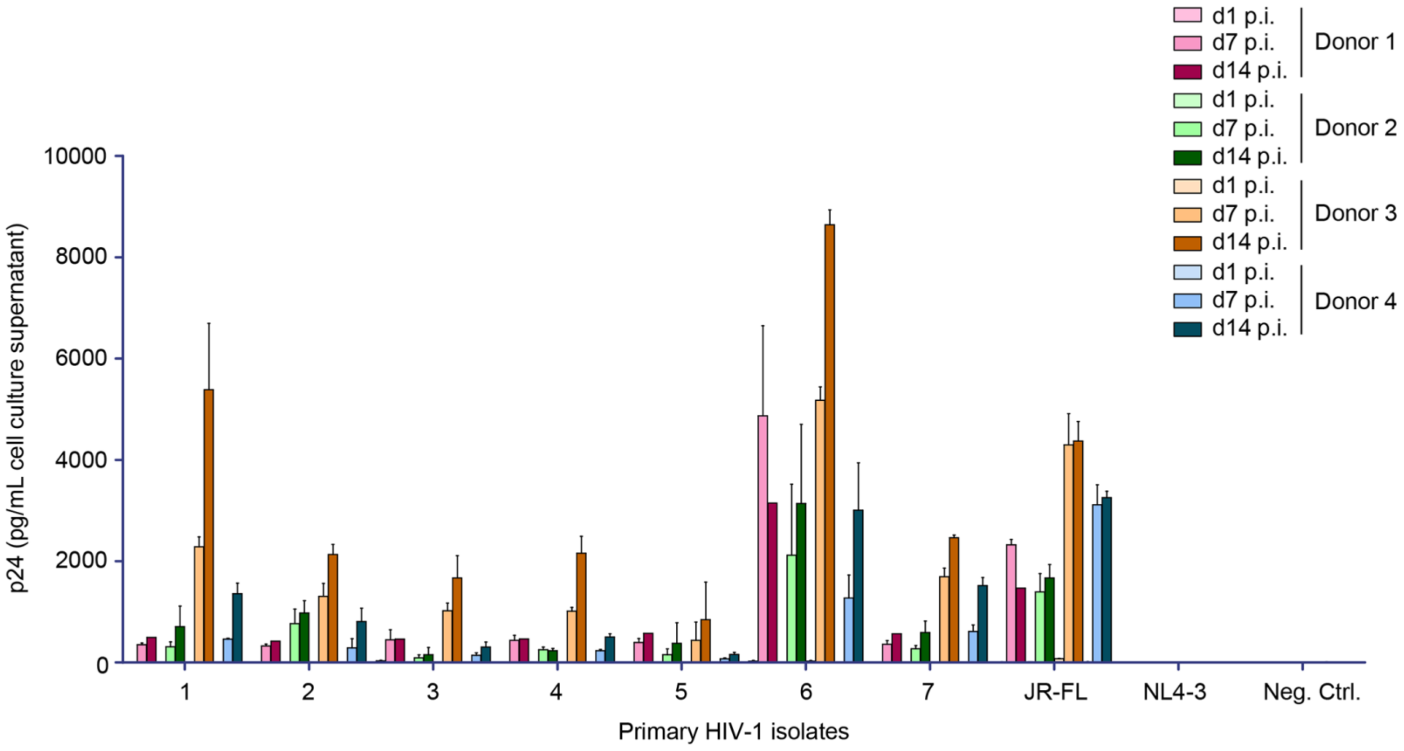

Supplementary Figure S1. Replication capacities of primary HIV-1 isolates in monocyte-derived macrophages.

Each primary HIV-1 isolate was tested on MDMs from four donors in two independent experiments. Each colour group represents a different donor and each colour shade represents a different time point when the production of HIV-1 p24 antigen was measured with an ELISA. The mean of triplicate measurements is indicated by the height of the bars and error bars indicate the standard deviation. HIV-1$_{JR-FL}$ is a macrophage-tropic strain whereas HIV-1$_{NL4-3}$ is a non-macrophage-tropic strain.

Supplementary Figure S2

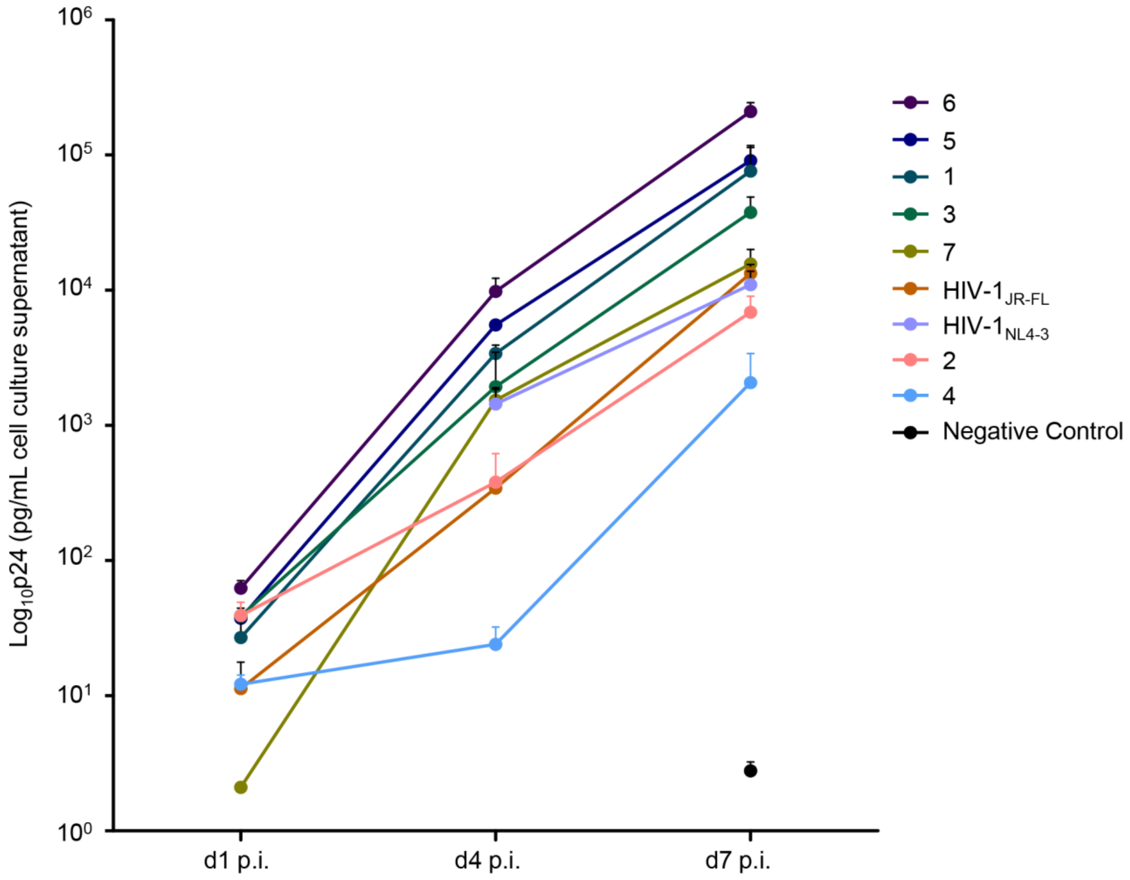

Supplementary Figure S2. Replication capacities of primary HIV-1 isolates in $CD4^+$ T cells.

Each primary HIV-1 isolate was tested on 3-way stimulated mixed donors´ PBMCs. HIV-1 p24 antigen production was monitored with an ELISA. Each dot represents the mean of a triplicate measurement and error bars indicate the standard deviation.

Supplementary Figure S3

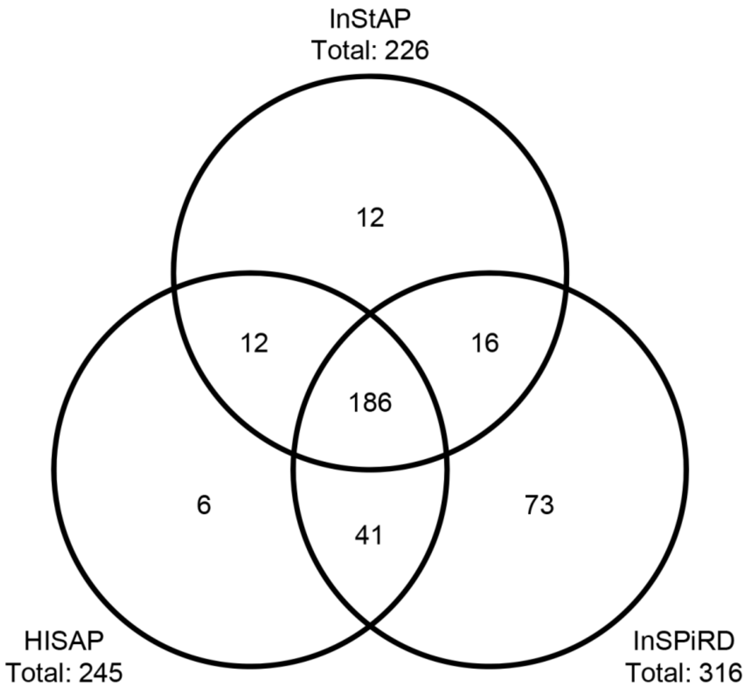

Supplementary Figure S3. Comparison of HIV-1 integration sites obtained via three mapping pipelines.

To test the reliability of our in-house mapping pipeline, i.e. Integration Site Analysis Pipeline (InStAP), we compared the same subset of our data with two other pipelines, i.e. Integration Site Pipeline in R and Database (InSPiRD) (courtesy of Frederic D. Bushman), and High-throughput Insertion Site Analysis Pipeline (HISAP)*.

*Arens, A. *et al.* Bioinformatic clonality analysis of next-generation sequencing-derived viral vector integration sites. *Human gene therapy methods* **23**, 111-118, doi:10.1089/hgtb.2011.219 (2012).

Supplementary Figure S4

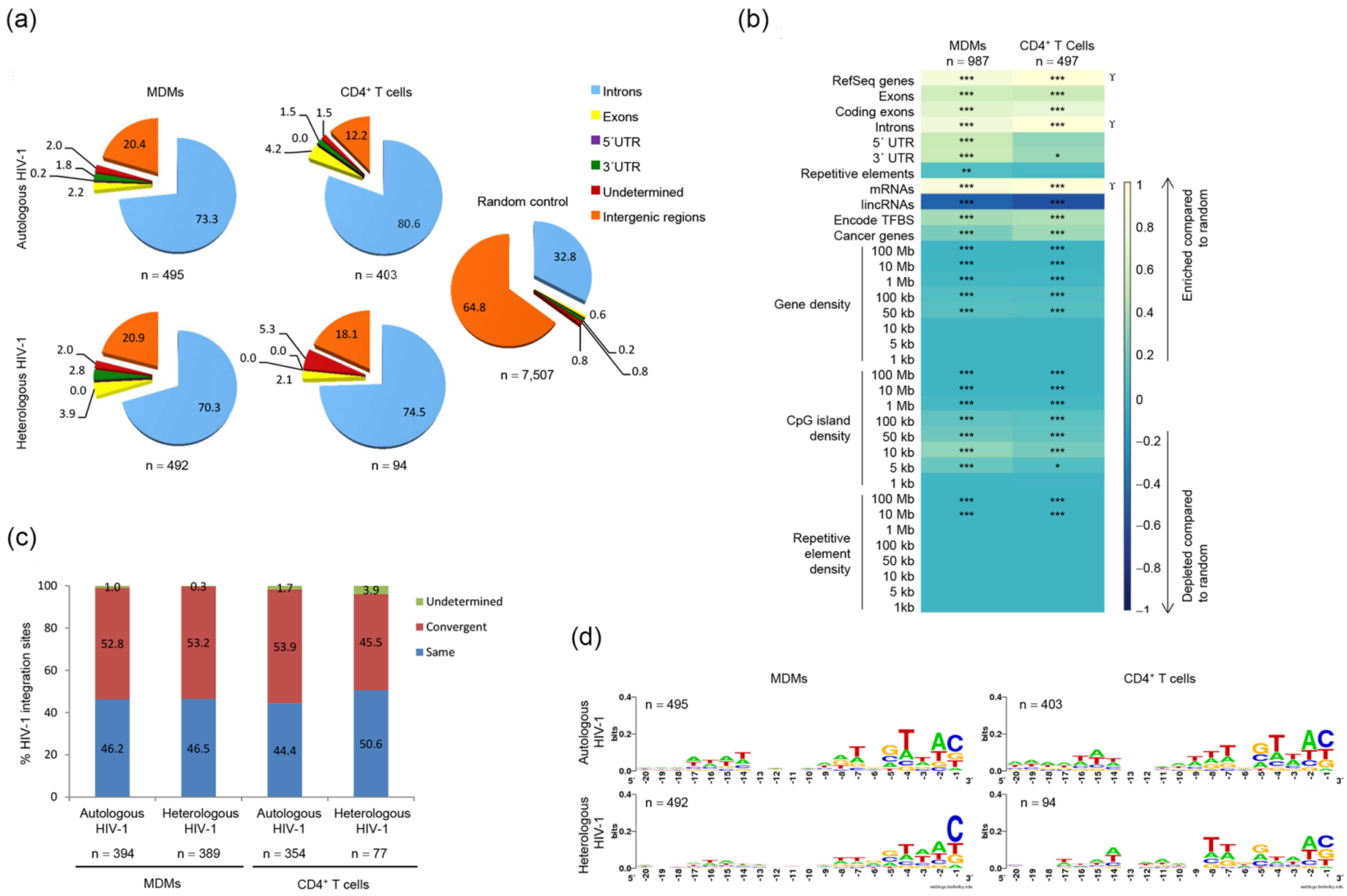

Supplementary Figure S4. Genetic patterns of HIV-1 integration sites in monocyte-derived macrophages and CD4$^+$ T cells

Cells were infected *ex vivo* with autologous and heterologous HIV-1 isolates. (a) Genic distribution of HIV-1 integration sites. (b) Various genetic features surrounding integrated HIV-1. Only repetitive elements at HIV-1 integration sites that could be mapped to a unique host genomic locus were considered. Two-tailed Fisher's exact test with 95% confidence interval: ***p<0.0001, **p <0.01, and *p <0.05 compared to random integration sites generated *in silico*; ϒ p<0.001 and Log_{10}OR<–0.3 between MDMs and CD4$^+$ T cells. (c) Transcription orientation of integrated HIV-1 relative to its hosting gene. Only intragenic HIV-1 integration sites were considered. (d) Adjacent nucleotide sequence upstream of integrated HIV-1.

Supplementary Figure S5

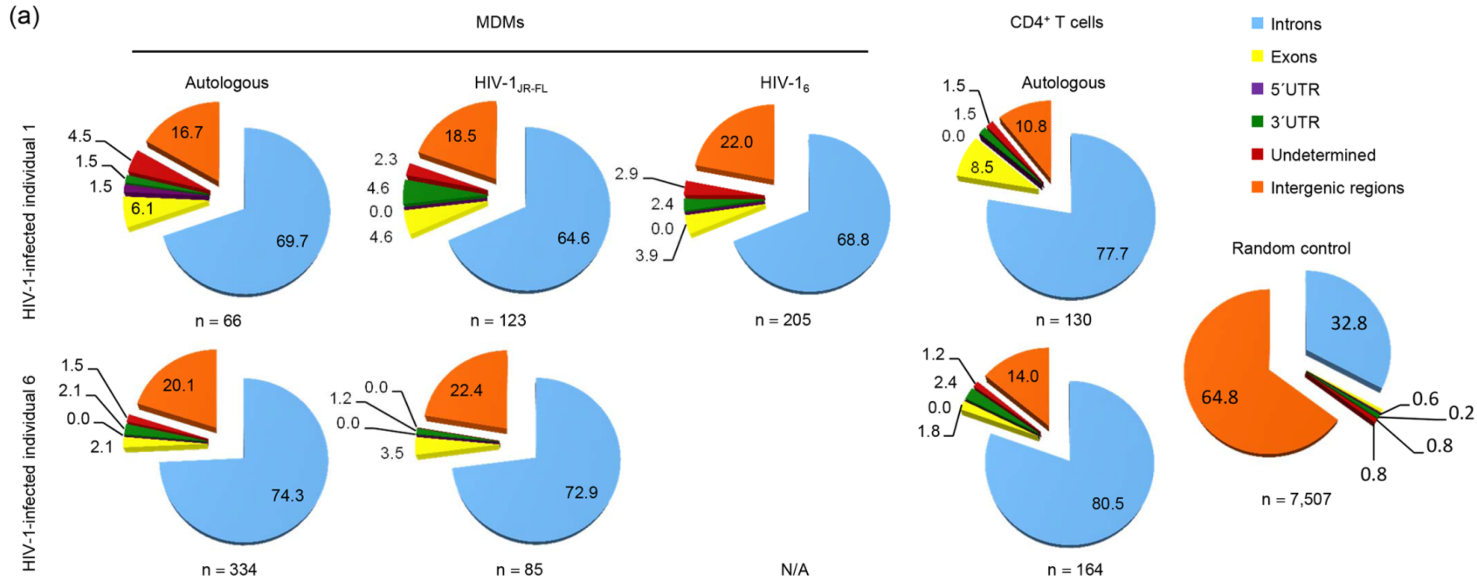

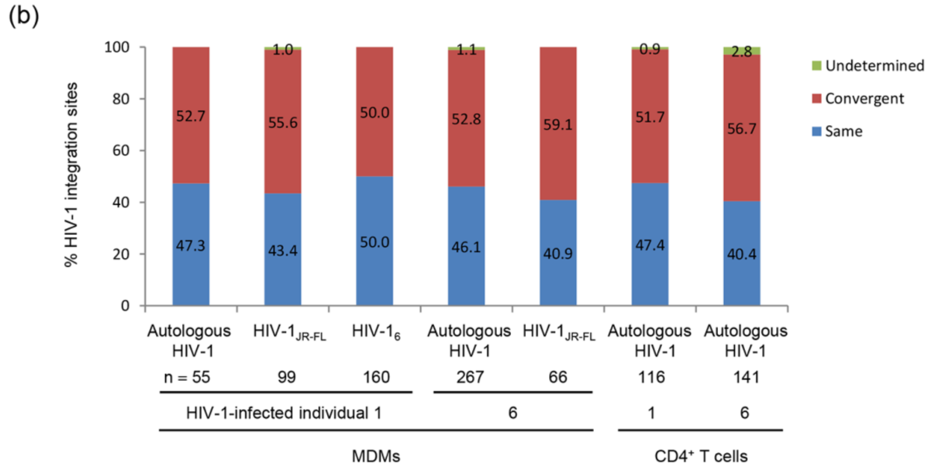

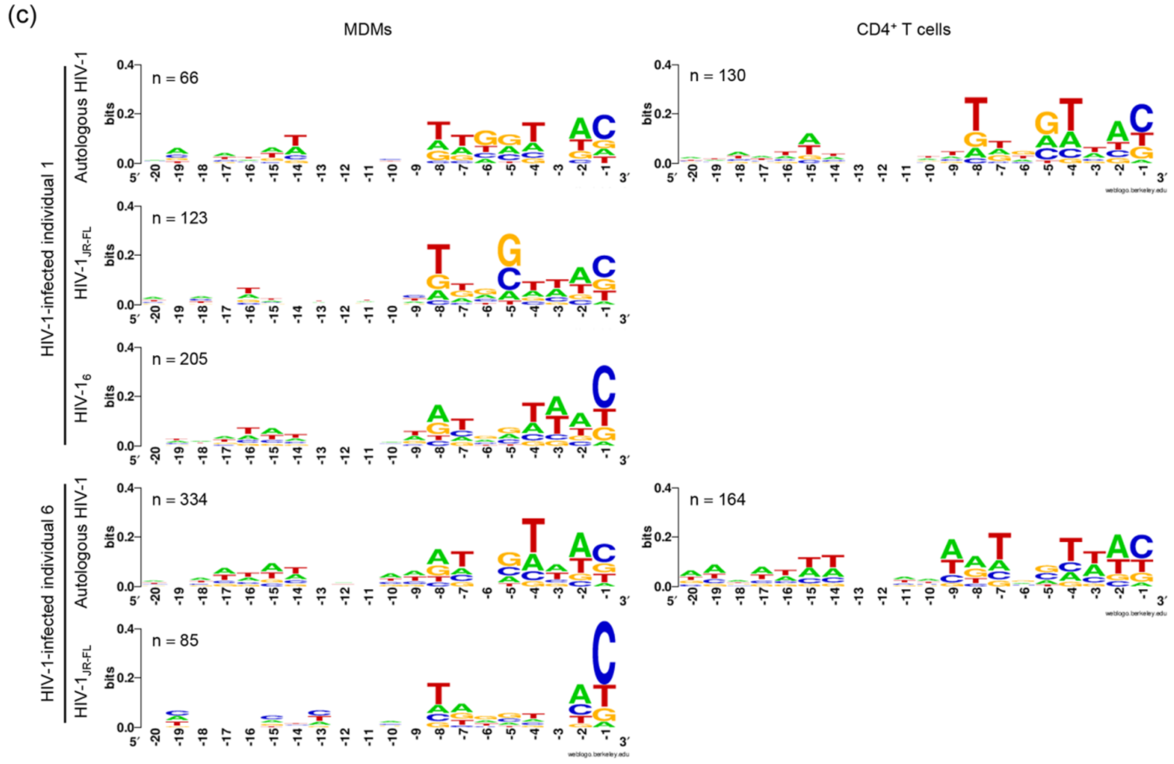

Supplementary Figure S5. Genetic patterns of HIV-1 integration sites in monocyte-derived macrophages and $CD4^+$ T cells of HIV-1-infected individuals 1 and 6

Cells were infected *ex vivo* with autologous and heterologous HIV-1 isolates. (a) Genic distribution of HIV-1 integration sites. (b) Transcription orientation of integrated HIV-1 relative to its hosting gene. Only intragenic HIV-1 integration sites were considered. (c) Adjacent nucleotide sequence upstream of integrated HIV-1. HIV-1_6 indicates primary HIV-1 isolate derived from HIV-1-infected individual 6 during the acute phase of infection.

Supplementary Figure S6

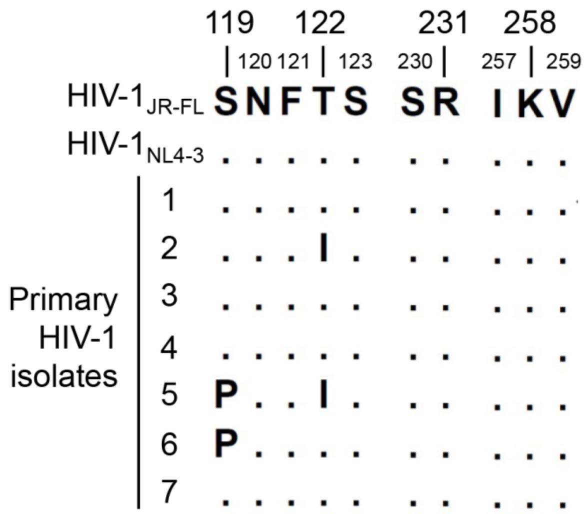

Supplementary Figure S6. Alignment of amino acid sequences of HIV-1 integrase of HIV-1$_{JR\text{-}FL}$, HIV-1$_{NL4\text{-}3}$, and primary HIV-1 isolates at positions 119, 122, 231, and 258.

Supplementary Figure S7

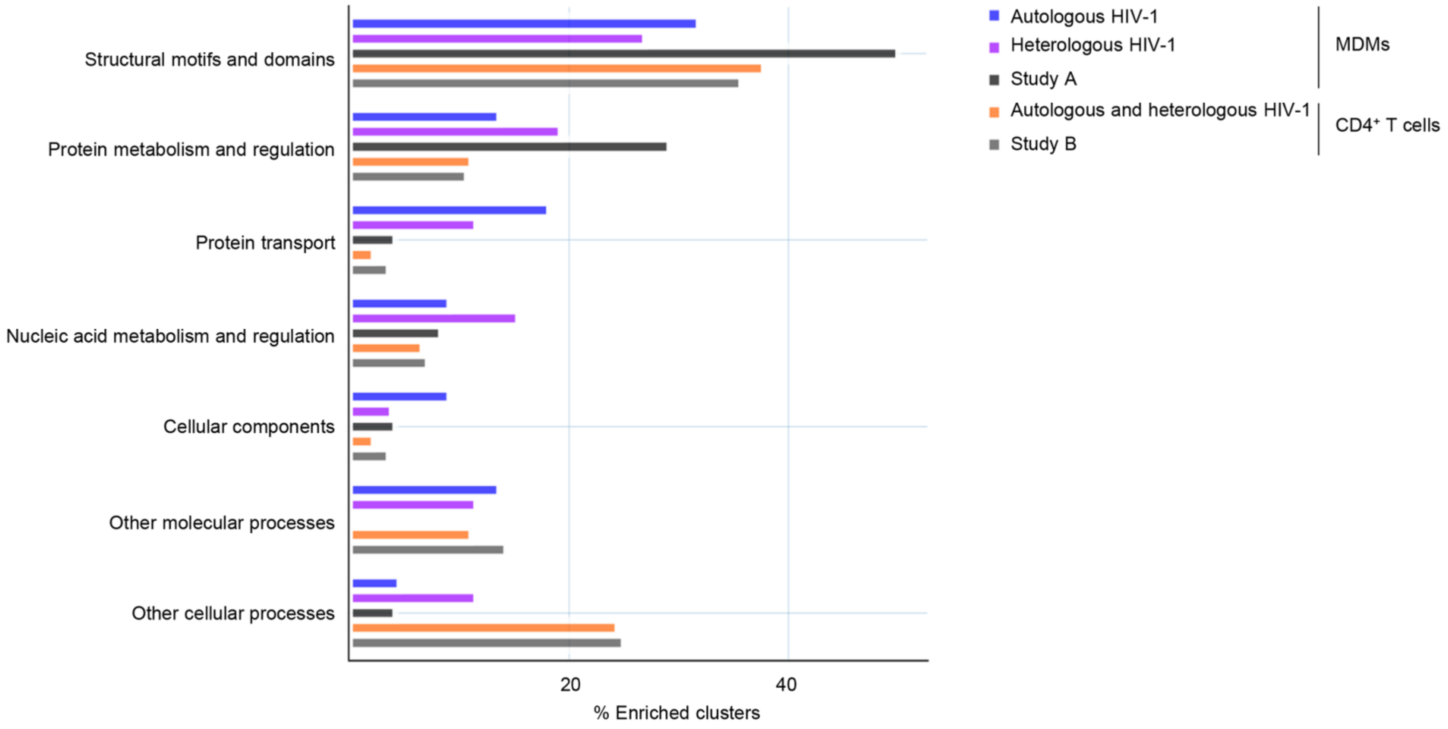

Supplementary Figure S7. Distribution of HIV-1 integration sites in DAVID gene clusters in monocyte-derived macrophages and CD4^{+} T cells.

Only DAVID structural and functional gene clusters that were over-represented, i.e. DAVID enrichment score >1.3 at high stringency, in the human genome were included in this analysis. Studies A [18] and B [27] examined HIV-1 integration sites in MDMs and CD4^{+} T cells, respectively.

Supplementary Figure S8

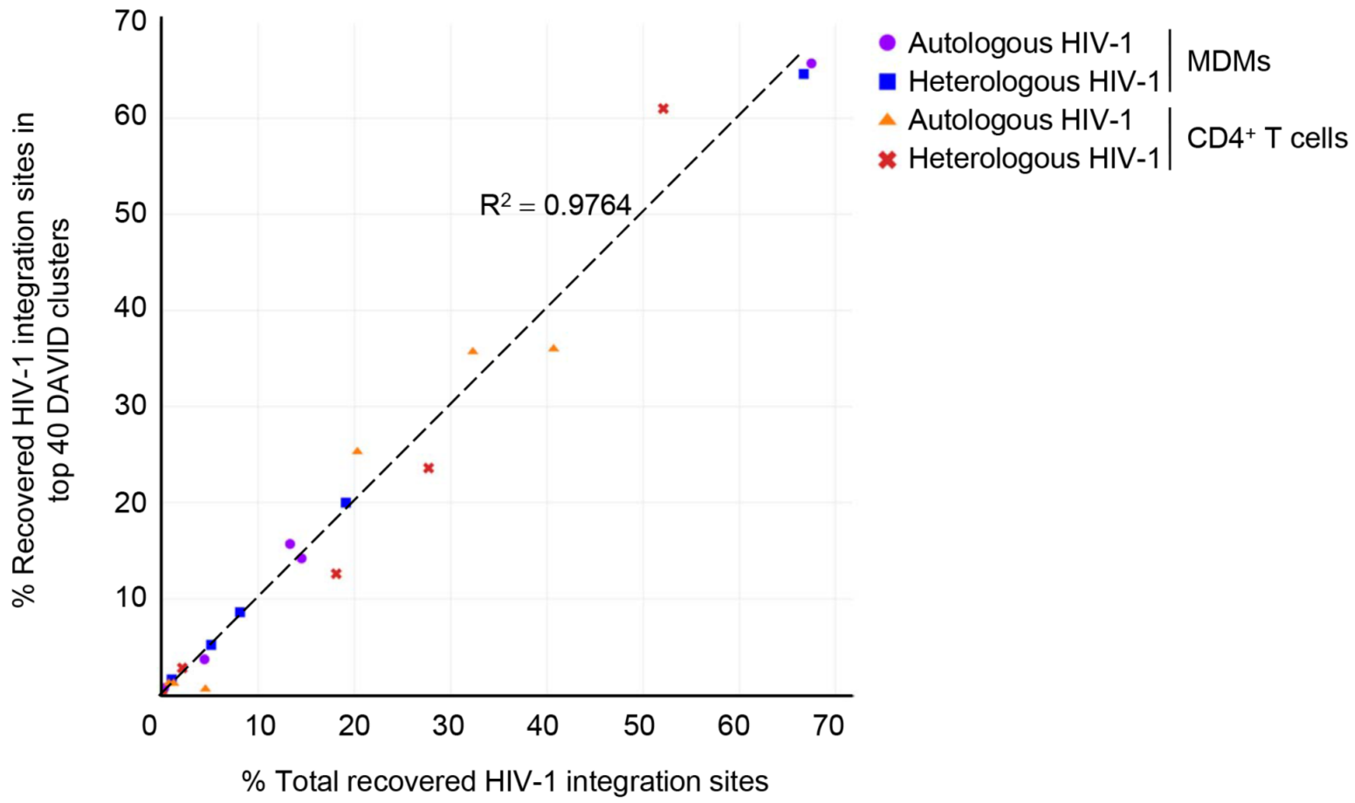

Supplementary Figure S8. Contribution of recovered HIV-1 integration sites from each sample towards the formation of the top 40 DAVID gene clusters at high stringency.

Each colour group represents a specific cell type and HIV-1 sample and each point within a colour group represents a different HIV-1-infected individual's sample. For instance, 65.7% of HIV-1-hosting genes derived from MDMs infected *ex vivo* with autologous primary HIV-1 isolates in the top 40 DAVID gene clusters originated from HIV-1-infected individual 6, whose MDMs when infected *ex vivo* with his autologous primary HIV-1 isolate yielded 67.5% of all HIV-1 integration sites recovered in this specific cell type and HIV-1 set-up.

Supplementary Table S1. Characteristics of HIV-1-infected individuals at the time of *ex vivo* infection of their monocyte-derived macrophages and $CD4^+$ T cells.

HIV-1-infected individual	Gender	Ethnicity	Transmission Route	Duration of uninterrupted ART	Duration of viral load <20 HIV-1 RNA copies/mL plasma	$CD4^+$ T cell count (μL^{-1} blood)	Number of days between culture of primary HIV-1 isolates and date of estimated infection	Drug regimen
1	Male	Caucasian	MSM	6.1 yr	3.3 yr	624	153	3TC<br>ABC<br>RTV/DRV
2	Male	Caucasian	MSM	7.8 yr	3.3 yr	274	45	ETC<br>TNV<br>EFV
3	Male	Caucasian	MSM	5.3 yr	2.0 yr	638	45	3TC<br>ABC<br>RTV/DRV
4	Male	Caucasian	MSM	4.4 yr	3.4 yr	594	35	ETC<br>TNV<br>RGV
5	Male	Hispanic	MSM	3.2 yr	2.8 yr	562	44	ETC<br>TNV<br>RTV/DRV
6	Male	Caucasian	Bisexual	2.8 yr	1.5 yr	587	29	ETC<br>TNV<br>RTV/DRV
7	Male	Caucasian	MSM	3.0 yr	2.7 yr	906	15	ETC<br>TNV<br>RTV/DRV

MSM: Men who have sex with men; 3TC: Lamivudine; ABC: Abacavir; RTV: Ritonavir; DRV: Darunavir; ETC: Emtricitabine; TNV: Tenofovir; EFV: Efavirenz; RGV: Raltegravir

Supplementary Table S2. Number of integration sites derived from seven HIV-1-infected individuals whose monocyte-derived macrophages and CD4+ T cells were infected *ex vivo* with autologous and heterologous HIV-1 isolates.

**HIV-1-infected individuals**	**Autologous HIV-1**		**Heterologous HIV-1**			
	**MDMs**	**CD4+ T cells**	**MDMs**		**CD4+ T cells**	
1	66	130	123	*JR-FL*	26	*JR-FL*
			205	6		
2	72	82	32	*JR-FL*	17	*JR-FL*
			8	*1*		
3	1	18	-		-	
4	22	3	5	*6*	-	
5	0	1	25	*6*	49	*NL4-3*
6	334	164	85	*JR-FL*	2	*JR-FL*
			9	*1*		
7	0	5	-		-	
Total	495	403	492		94	

Autologous primary HIV-1 isolates were derived from the same HIV-1-infected individuals during the acute phase of infection whose cells were infected *ex vivo*. Heterologous HIV-1 isolates was either primary HIV-1 isolates derived from other HIV-1-infected individuals or clonal HIV-1 strains. The heterologous HIV-1 isolates used for each sample are indicated next to the number of integration sites recovered. ‘-’ indicates samples whose HIV-1 integration sites were not amplified.

Supplementary Table S3. **DAVID structural and functional clusters overlap between data sets with only, and without, HIV-1 integration sites derived from HIV-1-infected individuals 1 and/or 6 and the entire corresponding data sets.**

**Data set 1**	**Data set 2**	**Top 40 DAVID cluster overlap (%)**
MDM/HIV-1_a	MDM/HIV-1_a without 6	42.5
	MDM/HIV-1_a only 6	67.5
MDM/HIV-1_h	MDM/HIV-1_h without 6	57.5
	MDM/HIV-1_h only 6	57.5
MDM/HIV-1_a	MDM/HIV-1_a without 1 and 6	34.8
	MDM/HIV-1_a only 1	47.8
$CD4^+$ T cell/HIV-1_a*	$CD4^+$ T cell/HIV-1_a without 1 and 6*	51.4
	$CD4^+$ T cell/HIV-1_a only 1*	51.4

HIV-1 hosting genes were clustered based on their structural and functional annotations using DAVID. The top 40 clusters from all data sets were compared and clusters having the same or highly similar annotations were scored.

MDM: Monocyte-derived macrophages; a: autologous; h: heterologous

*Only 37 clusters were analysed.

Supplementary Table S4

HIV-1-infected individual	type	HIV-1	Amplicon start (Adaptor end)	Amplicon end (5´ HIV-1 LTR end)	Chr	Strand	RefSeq name	RefSeq number	Intragenic/ Intergenic
1	MDM	6	183653673	183653796	chr3	-	ABCC5	NM_005688	Intron
1	CD4 T	1	31666793	31666859	chr6	+	ABHD16A	NM_001177515	Intron
1	MDM	6	1005443	1005566	chr17	-	ABR	NM_001092	Intron
1	MDM	1	35665244	35665319	chr17	-	ACACA	NM_198836	Intron
1	MDM	6	59013485	59013585	chr15	-	ADAM10	NM_001110	Intron
1	MDM	6	59021068	59021212	chr15	-	ADAM10	NM_001110	Intron
1	MDM	1	36451344	36451401	chr1	+	AGO3	NM_024852	Intron
1	MDM	6	36291642	36291666	chr1	+	AGO4	NM_017629	Intron
1	CD4 T	JR-FL	86241079	86241185	chr15	+	AKAP13	NM_006738	Intron
1	MDM	6	118088761	118088831	chr11	+	AMICA1	NM_001286570	Intron
1	MDM	1	110825391	110825541	chr12	-	ANAPC7	NM_016238	Intron
1	MDM	JR-FL	91968988	91969023	chr7	-	ANKIB1	NM_019004	Intron
1	MDM	6	133302821	133302976	chr12	+	ANKLE2	NM_015114	3´UTR
1	MDM	JR-FL	47431269	47431329	chrX	+	ARAF	NM_001256196	3´UTR
1	CD4 T	1	36142656	36142684	chr4	-	ARAP2	NM_015230	Intron
1	CD4 T	1	155920936	155921024	chr1	-	ARHGEF2	NM_004723	Exon
1	CD4 T	1	135765185	135765343	chrX	-	ARHGEF6	NM_004840	Intron
1	MDM	6	27100147	27100176	chr1	+	ARID1A	NM_139135	Exon
1	MDM	JR-FL	27055630	27055712	chr1	+	ARID1A	NM_139135	Intron
1	CD4 T	1	27051354	27051459	chr1	-	ARID1A	NM_139135	Intron
1	CD4 T	1	63427617	63427795	chr11	+	ATL3	NM_015459	Intron
1	MDM	JR-FL	42273570	42273614	chr17	+	ATXN7L3	NM_001098833	Intron
1	CD4 T	1	49463257	49463311	chr19	+	BAX	NM_001291429	Intron
1	MDM	6	35230525	35230583	chr14	+	BAZ1A	NM_013448	Intron
1	CD4 T	1	160203796	160204058	chr2	+	BAZ2B	NM_013450	Exon
1	MDM	JR-FL	30270845	30270906	chr20	-	BCL2L1	NM_001191	Intron
1	CD4 T	1	51689697	51689835	chr12	+	BIN2	NM_001290009	Intron
1	MDM	6	91277163	91277248	chr15	+	BLM	NM_001287248	Intron
1	MDM	6	134340957	134341011	chr7	+	BPGM	NM_001724	Intron
1	CD4 T	1	41227213	41227285	chr17	+	BRCA1	NM_007298	Intron
1	MDM	6	40670131	40670192	chr21	+	BRWD1	NM_018963	Undetermined
1	MDM	JR-FL	29755475	29755544	chr16	+	C16orf54	NM_175900	3´UTR
1	MDM	1	76152974	76153045	chr17	+	C17orf99	NM_001163075	Intron
1	MDM	1	76154198	76154253	chr17	+	C17orf99	NM_001163075	Intron
1	CD4 T	1	7286610	7286701	chr7	-	C1GALT1	NM_020156	3´UTR
1	MDM	6	43236048	43236098	chr1	+	C1orf50	NR_040733	Intron
1	MDM	JR-FL	31887331	31887372	chr6	-	C2	NM_001282457	Intron
1	MDM	JR-FL	31908596	31908661	chr6	+	C2	NM_001282457	Intron
1	MDM	JR-FL	73766807	73766858	chr11	+	C2CD3	NM_001286577	Intron
1	CD4 T	JR-FL	36858657	36858707	chr6	-	C6orf89	NM_001286636	Intron
1	CD4 T	1	74542497	74542530	chr9	-	C9orf85	NM_182505	Intron
1	MDM	6	138719206	138719266	chr9	-	CAMSAP1	NM_015447	Intron
1	CD4 T	JR-FL	64961903	64961988	chr11	-	CAPN1	NM_001198868	Intron
1	MDM	6	3058755	3058786	chr7	+	CARD11	NM_032415	Intron
1	CD4 T	1	48718372	48718426	chr19	-	CARD8	NR_033679	Intron
1	CD4 T	1	67074002	67074091	chr16	-	CBFB	NM_022845	Intron
1	MDM	1	46168406	46168434	chr17	+	CBX1	NM_006807	Intron
1	MDM	6	14020600	14020624	chr19	+	CC2D1A	NM_017721	Intron
1	CD4 T	1	47010397	47010463	chr3	+	CCDC12	NR_102269	Intron
1	MDM	6	92908343	92908397	chr7	-	CCDC132	NM_017667	Intron

1	MDM	JR-FL	78074254	78074369	chr17	+	CCDC40	NM_017950	3´UTR
1	MDM	1	80097727	80097855	chr17	-	CCDC57	NM_198082	Intron
1	MDM	6	80100757	80100777	chr17	+	CCDC57	NM_198082	Intron
1	MDM	1	1327971	1328090	chr1	-	CCNL2	NM_030937	Undetermined
1	MDM	1	1328694	1328716	chr1	-	CCNL2	NM_030937	Intron
1	MDM	JR-FL	135688143	135688247	chr2	-	CCNT2	NR_037649	Intron
1	MDM	6	229470015	229470077	chr1	-	CCSAP	NM_145257	Intron
1	MDM	6	112678457	112678496	chr3	+	CD200R1	NM_138806	Intron
1	CD4 T	1	35612963	35612995	chr9	-	CD72	NM_001782	Exon
1	CD4 T	1	130693385	130693455	chr5	-	CDC42SE2	NM_020240	Intron
1	MDM	6	44369067	44369195	chr6	-	CDC5L	NM_001253	Intron
1	MDM	6	73378017	73378075	chr10	-	CDH23	NM_052836	Intron
1	MDM	6	21189996	21190140	chr6	-	CDKAL1	NM_017774	Intron
1	MDM	JR-FL	5140471	5140510	chr20	+	CDS2	NM_003818	Intron
1	MDM	1	47511042	47511088	chr11	-	CELF1	NM_198700	Intron
1	MDM	6	88157440	88157491	chr3	-	CGGBP1	NM_001195308	Intron
1	MDM	JR-FL	25108663	25108749	chr1	+	CLIC4	NM_013943	Intron
1	MDM	JR-FL	65451453	65451495	chr15	-	CLPX	NM_006660	Intron
1	CD4 T	1	43724041	43724219	chr7	-	COA1	NR_047701	Intron
1	CD4 T	JR-FL	40716448	40716535	chr17	+	COASY	NM_025233	Exon
1	MDM	6	230791987	230792111	chr1	+	COG2	NM_007357	Intron
1	MDM	JR-FL	33132610	33132637	chr6	+	COL11A2	NM_080680	Exon
1	CD4 T	1	128986697	128986955	chr3	-	COPG1	NM_016128	Intron
1	MDM	6	198323755	198323871	chr2	-	COQ10B	NM_025147	Intron
1	MDM	1	145631048	145631146	chr8	+	CPSF1	NM_013291	Intron
1	CD4 T	1	87001422	87001512	chr7	-	CROT	NM_001143935	Intron
1	MDM	6	10787991	10788167	chr11	+	CTR9	NM_014633	Intron
1	CD4 T	1	156847253	156847442	chr4	+	CTSO	NM_001334	Intron
1	MDM	6	111738420	111738485	chr12	+	CUX2	NM_015267	Intron
1	MDM	6	64189690	64189762	chr5	-	CWC27	NM_005869	Intron
1	CD4 T	1	69471468	69471560	chr16	+	CYB5B	NM_030579	Intron
1	CD4 T	1	50796345	50796514	chr16	+	CYLD	NM_001042355	Intron
1	MDM	6	139604924	139604988	chr5	-	CYSTM1	NM_032412	Intron
1	MDM	6	76687033	76687152	chr17	-	CYTH1	NM_001292019	Intron
1	MDM	6	72136374	72136459	chr13	+	DACH1	NM_080759	Intron
1	CD4 T	1	87517139	87517194	chr7	+	DBF4	NM_006716	Intron
1	CD4 T	1	150128549	150128607	chr5	+	DCTN4	NM_001135644	Intron
1	CD4 T	1	38109691	38109725	chr8	+	DDHD2	NM_015214	Exon
1	CD4 T	1	15986766	15986894	chr1	+	DDI2	NM_032341	Undetermined
1	CD4 T	1	134695324	134695375	chrX	+	DDX26B	NM_182540	Intron
1	MDM	JR-FL	6471548	6471576	chr19	-	DENND1C	NM_001290331	Intron
1	MDM	6	13085547	13085576	chr8	+	DLC1	NM_182643	Intron
1	MDM	1	7103580	7103678	chr17	-	DLG4	NM_001128827	Intron
1	MDM	6	88572194	88572295	chr4	+	DMP1	NM_004407	Intron
1	CD4 T	1	100826703	100826933	chr4	-	DNAJB14	NM_001031723	Intron
1	MDM	6	10300648	10300719	chr19	+	DNMT1	NM_001130823	Intron
1	CD4 T	1	431190	431391	chr9	+	DOCK8	NM_203447	Intron
1	MDM	6	37614945	37614970	chr21	+	DOPEY2	NM_005128	Intron
1	MDM	6	65107208	65107291	chr11	+	DPF2	NM_006268	Intron
1	MDM	6	105445597	105445631	chr8	+	DPYS	NM_001385	Intron
1	MDM	6	28677932	28678000	chr18	-	DSC2	NM_004949	Intron
1	MDM	1	2283918	2283968	chr16	-	E4F1	NM_001288778	Undetermined
1	CD4 T	1	3854389	3854447	chr12	+	EFCAB4B	NM_001144958	Intron
1	MDM	JR-FL	14853641	14853672	chr19	+	EMR2	NM_001271052	Intron
1	MDM	6	40273157	40273201	chr22	+	ENTHD1	NM_152512	Intron
1	CD4 T	1	51938586	51938788	chr1	-	EPS15	NM_001981	Exon

1	MDM	JR-FL	98716434	98716517	chr9	-	ERCC6L2	NM_001010895	Intron
1	MDM	6	62146277	62146324	chr17	-	ERN1	NM_001433	Intron
1	MDM	JR-FL	45764912	45764955	chr20	+	EYA2	NM_172110	Intron
1	MDM	1	28383286	28383401	chr1	-	EYA3	NM_001282561	Intron
1	MDM	6	28362811	28362911	chr1	+	EYA3	NM_001282562	Intron
1	MDM	6	175904741	175904873	chr5	+	FAF2	NM_014613	Intron
1	MDM	JR-FL	40748993	40749022	chr17	+	FAM134C	NR_026697	Intron
1	MDM	6	67577818	67577908	chr16	-	FAM65A	NM_024519	Intron
1	CD4 T	1	124790015	124790086	chr8	-	FAM91A1	NM_144963	Intron
1	CD4 T	1	33812540	33812741	chr2	+	FAM98A	NM_015475	Intron
1	MDM	JR-FL	5487773	5487911	chr6	-	FARS2	NM_006567	Intron
1	MDM	6	37522943	37523084	chr17	+	FBXL20	NM_001184906	Intron
1	MDM	6	48415373	48415414	chr3	-	FBXW12	NM_207102	Intron
1	MDM	1	72719579	72719685	chr11	+	FCHSD2	NM_014824	Intron
1	MDM	6	72653001	72653150	chr11	-	FCHSD2	NM_014824	Intron
1	MDM	6	72676762	72676835	chr11	-	FCHSD2	NM_014824	Intron
1	MDM	6	130961005	130961086	chrX	+	FIRRE	NR_026975	Intron
1	MDM	JR-FL	50064687	50064780	chr12	+	FMNL3	NM_198900	Intron
1	MDM	6	216235137	216235257	chr2	+	FN1	NM_212476	Intron
1	MDM	JR-FL	910765	910852	chr4	+	GAK	NM_005255	Intron
1	CD4 T	1	62396371	62396462	chr11	-	GANAB	NM_001278192	Exon
1	CD4 T	JR-FL	110743025	110743085	chr4	-	GAR1	NM_032993	Intron
1	MDM	6	130117502	130117693	chr9	+	GARNL3	NM_001286779	Undetermined
1	CD4 T	1	30648147	30648263	chr7	+	GARS	NM_002047	Intron
1	MDM	6	153853836	153853890	chr1	+	GATAD2B	NM_020699	Intron
1	MDM	1	104135495	104135572	chr10	-	GBF1	NM_004193	Intron
1	CD4 T	JR-FL	109091326	109091452	chr2	+	GCC2	NR_028063	Intron
1	MDM	1	2035545	2035588	chr16	-	GFER	NM_005262	Intron
1	CD4 T	1	85907612	85907664	chr10	+	GHITM	NM_014394	Intron
1	MDM	JR-FL	100280030	100280067	chr7	-	GIGYF1	NM_022574	Exon
1	CD4 T	1	110128275	110128363	chr1	+	GNAI3	NM_006496	Intron
1	MDM	6	121440423	121440444	chr3	+	GOLGB1	NM_001256487	Intron
1	MDM	JR-FL	155802999	155803038	chr1	-	GON4L	NM_001282860	Intron
1	MDM	6	42496546	42496642	chr17	-	GPATCH8	NR_036474	Intron
1	MDM	6	42574466	42574507	chr17	-	GPATCH8	NR_036474	Intron
1	MDM	1	73379070	73379206	chr17	+	GRB2	NM_002086	Intron
1	MDM	6	48842246	48842305	chrX	-	GRIPAP1	NM_020137	Intron
1	CD4 T	JR-FL	39114816	39114980	chr22	-	GTPBP1	NM_004286	Intron
1	CD4 T	1	31432350	31432428	chr6	+	HCP5	NR_040662	3´UTR
1	MDM	6	83750574	83750634	chrX	+	HDX	NM_001177478	Intron
1	MDM	JR-FL	43242631	43242707	chr17	+	HEXIM2	NM_144608	Intron
1	CD4 T	1	42271816	42271959	chr1	+	HIVEP3	NM_024503	Intron
1	MDM	6	1739040	1739107	chr16	-	HN1L	NM_144570	Intron
1	MDM	JR-FL	8540993	8541083	chr19	-	HNRNPM	NM_031203	Intron
1	MDM	JR-FL	30571688	30571773	chr22	+	HORMAD2	NM_152510	Intron
1	CD4 T	1	16257297	16257493	chr19	-	HSH2D	NR_111904	Intron
1	MDM	1	3083233	3083284	chr4	-	HTT	NM_002111	Intron
1	CD4 T	1	48005910	48006041	chr7	+	HUS1	NM_004507	Intron
1	CD4 T	1	118429856	118429986	chr11	+	IFT46	NM_001168618	Intron
1	MDM	6	27018566	27018598	chr9	+	IFT74	NM_001099223	Intron
1	MDM	JR-FL	185484866	185484915	chr3	-	IGF2BP2	NM_001291872	Intron
1	MDM	JR-FL	1534456	1534504	chr7	+	INTS1	NM_001080453	Exon
1	CD4 T	1	153701475	153701528	chr1	+	INTS3	NM_023015	Intron
1	MDM	6	61880138	61880283	chr5	+	IPO11	NM_016338	Intron
1	CD4 T	1	78757798	78757842	chr15	-	IREB2	NM_004136	Intron
1	CD4 T	JR-FL	182395885	182396059	chr2	+	ITGA4	NM_000885	Intron

1	MDM	JR-FL	30523931	30523987	chr16	-	ITGAL	NM_001114380	Intron
1	MDM	6	35173026	35173094	chr21	-	ITSN1	NM_001001132	Intron
1	MDM	6	74714348	74714469	chr17	-	JMJD6	NM_001081461	Intron
1	MDM	JR-FL	42121278	42121311	chr15	+	JMJD7	NM_001114632	Intron
1	MDM	JR-FL	44197597	44197650	chr17	-	KANSL1	NM_001193466	Intron
1	CD4 T	1	44283636	44283952	chr17	-	KANSL1	NM_001193465	Intron
1	CD4 T	1	44144563	44144644	chr17	-	KANSL1	NM_001193466	Intron
1	CD4 T	1	29924627	29924729	chr16	-	KCTD13	NM_178863	Intron
1	MDM	6	66967496	66967623	chr11	+	KDM2A	NR_027473	Intron
1	MDM	6	66978531	66978587	chr11	+	KDM2A	NR_027473	Intron
1	CD4 T	JR-FL	58956591	58956631	chr14	+	KIAA0586	NM_001244189	Intron
1	MDM	6	86558672	86558723	chr7	+	KIAA1324L	NM_001291990	Intron
1	MDM	6	61350733	61350778	chr2	-	KIAA1841	NM_001129993	Undetermined
1	MDM	JR-FL	29808625	29808668	chr16	+	KIF22	NM_001256269	Intron
1	MDM	6	104113395	104113542	chr14	+	KLC1	NM_005552	Intron
1	MDM	JR-FL	51396761	51396882	chr19	-	KLKP1	NR_002948	Intron
1	CD4 T	1	9142900	9142999	chr12	+	KLRG1	NM_005810	Intron
1	CD4 T	1	118366439	118366497	chr11	-	KMT2A	NM_001197104	Exon
1	CD4 T	1	118388928	118389005	chr11	-	KMT2A	NM_001197104	Intron
1	CD4 T	1	36225981	36226090	chr19	-	KMT2B	NM_014727	Intron
1	CD4 T	1	123058110	123058192	chr12	+	KNTC1	NM_014708	Intron
1	MDM	1	98708776	98708875	chr10	-	LCOR	NM_001170766	5´UTR
1	CD4 T	1	11209856	11209972	chr19	+	LDLR	NM_001195803	Intron
1	MDM	6	156691912	156691938	chr3	+	LEKR1	NM_001004316	Intron
1	MDM	6	9758931	9759014	chr8	+	LINC00599	NR_024281	Undetermined
1	MDM	JR-FL	41034675	41034709	chr17	+	LINC00671	NR_027254	Intron
1	MDM	6	66919822	66919931	chr15	-	LINC01169	NR_110372	Intron
1	MDM	1	67091255	67091295	chr11	-	LOC100130987	NR_024469	Intron
1	MDM	6	67094712	67094762	chr11	+	LOC100130987	NR_024469	Intron
1	CD4 T	1	14251673	14251781	chr19	+	LOC100507373	NR_045214	Intron
1	MDM	1	38718622	38718689	chr2	-	LOC101929596	NR_110259	Intron
1	CD4 T	1	47448011	47448189	chr17	+	LOC102724596	NR_110883	Intron
1	MDM	6	110867120	110867193	chr1	+	LOC440600	NR_036595	Intron
1	MDM	JR-FL	243055066	243055176	chr2	-	LOC728323	NR_024437	Intron
1	CD4 T	1	70651648	70651734	chr1	+	LRRC40	NM_017768	Intron
1	MDM	6	117825707	117825751	chr7	-	LSM8	NM_016200	Exon
1	CD4 T	1	2216487	2216614	chr7	-	MAD1L1	NM_001013837	Intron
1	MDM	6	66698055	66698083	chr15	-	MAP2K1	NM_002755	Intron
1	MDM	JR-FL	67954171	67954245	chr15	+	MAP2K5	NM_002757	Intron
1	CD4 T	1	65369819	65369929	chr11	-	MAP3K11	NM_002419	Intron
1	MDM	1	43375369	43375394	chr17	-	MAP3K14	NM_003954	Intron
1	MDM	6	43349982	43350024	chr17	-	MAP3K14	NM_003954	Intron
1	MDM	6	36046658	36046681	chr6	-	MAPK14	NM_139013	Intron
1	MDM	6	55521433	55521548	chr14	+	MAPK1IP1L	NM_144578	Intron
1	CD4 T	1	3843344	3843428	chr20	+	MAVS	NM_020746	Intron
1	MDM	JR-FL	97986752	97986799	chr13	+	MBNL2	NM_207304	Intron
1	CD4 T	1	47659886	47659943	chr21	-	MCM3AP-AS1	NR_110566	Intron
1	MDM	6	121612577	121612630	chr10	+	MCMBP	NM_001256378	Exon
1	CD4 T	1	153310325	153310348	chrX	+	MECP2	NM_004992	Intron
1	CD4 T	1	116681165	116681270	chr12	-	MED13L	NM_015335	Intron
1	MDM	6	16707551	16707577	chr19	+	MED26	NM_004831	Intron
1	MDM	6	112670424	112670588	chr2	-	MERTK	NM_006343	Intron
1	CD4 T	1	74763238	74763492	chr17	+	MFSD11	NM_001242534	Exon
1	CD4 T	1	99300282	99300398	chr2	-	MGAT4A	NM_012214	Intron
1	MDM	JR-FL	4701583	4701617	chr16	+	MGRN1	NM_001142291	Intron
1	MDM	JR-FL	1490838	1490903	chr7	-	MICALL2	NM_182924	Intron

1	MDM	JR-FL	4741952	4742027	chr17	-	MINK1	NM_015716	Intron
1	MDM	6	19937144	19937293	chr1	-	MINOS1	NM_001204083	Intron
1	CD4 T	1	64892737	64892835	chr14	+	MIR548AZ	NR_106755	Undetermined
1	MDM	JR-FL	156056201	156056264	chr1	+	MIR7851	NR_107005	Intron
1	CD4 T	JR-FL	40852779	40853063	chr22	-	MKL1	NM_001282660	Intron
1	MDM	1	75516434	75516537	chr14	+	MLH3	NM_014381	Intron
1	MDM	1	36882978	36883313	chr17	+	MLLT6	NM_005937	3´UTR
1	MDM	JR-FL	145268050	145268188	chr8	-	MROH1	NM_001099281	Undetermined
1	MDM	JR-FL	55129936	55129992	chr1	+	MROH7	NM_001039464	Intron
1	MDM	6	78814198	78814258	chr4	+	MRPL1	NM_020236	Intron
1	MDM	6	16030234	16030277	chr8	-	MSR1	NM_138715	Intron
1	MDM	JR-FL	50958074	50958154	chr19	-	MYBPC2	NM_004533	Intron
1	MDM	JR-FL	52505073	52505164	chr15	-	MYO5C	NM_018728	Intron
1	CD4 T	1	40126417	40126493	chr4	-	N4BP2	NM_018177	Intron
1	MDM	JR-FL	50842950	50843014	chr19	+	NAPSB	NR_002798	Intron
1	MDM	6	15660161	15660199	chr2	-	NBAS	NR_052013	Intron
1	MDM	1	41338604	41338677	chr17	-	NBR1	NM_031862	Intron
1	MDM	6	41328708	41328823	chr17	+	NBR1	NM_031862	Intron
1	CD4 T	JR-FL	179326385	179326532	chr3	+	NDUFB5	NM_001199958	Intron
1	MDM	JR-FL	69666550	69666594	chr16	+	NFAT5	NM_173215	Intron
1	MDM	6	69679980	69680097	chr16	-	NFAT5	NM_173215	Intron
1	CD4 T	1	68195081	68195233	chr16	-	NFATC3	NM_173163	Intron
1	CD4 T	JR-FL	68198410	68198457	chr16	-	NFATC3	NM_173165	Intron
1	CD4 T	1	31524616	31524702	chr6	-	NFKBIL1	NM_001144962	Intron
1	CD4 T	JR-FL	33298636	33298716	chr9	-	NFX1	NM_147134	Intron
1	MDM	6	162050521	162050618	chr1	+	NOS1AP	NM_001164757	Intron
1	MDM	1	79596778	79596967	chr17	+	NPLOC4	NM_017921	Intron
1	MDM	1	79572675	79572701	chr17	+	NPLOC4	NM_017921	Intron
1	CD4 T	1	79588674	79588926	chr17	+	NPLOC4	NM_017921	Intron
1	CD4 T	JR-FL	52283843	52283980	chr1	-	NRD1	NM_001242361	Intron
1	CD4 T	1	126207515	126207554	chr8	-	NSMCE2	NM_173685	Intron
1	MDM	6	104878734	104878788	chr10	-	NT5C2	NM_012229	Intron
1	MDM	JR-FL	34320943	34321021	chr6	-	NUDT3	NM_006703	Intron
1	MDM	1	73805603	73805764	chr14	+	NUMB	NM_001005744	Intron
1	MDM	1	47839600	47839688	chr11	+	NUP160	NM_015231	Intron
1	CD4 T	1	44704204	44704231	chr7	-	OGDH	NM_001003941	Intron
1	MDM	JR-FL	65948913	65948975	chr11	+	PACS1	NM_018026	Intron
1	CD4 T	1	2570481	2570631	chr17	-	PAFAH1B1	NM_000430	Exon
1	CD4 T	1	196470253	196470350	chr3	-	PAK2	NM_002577	Intron
1	MDM	JR-FL	69623444	69623518	chr15	+	PAQR5	NM_017705	Intron
1	MDM	6	52634196	52634253	chr3	+	PBRM1	NM_018313	Intron
1	MDM	JR-FL	128684435	128684485	chr9	-	PBX3	NR_024122	Intron
1	MDM	JR-FL	70244756	70244815	chr2	-	PCBP1-AS1	NR_033872	Intron
1	MDM	JR-FL	53857571	53857609	chr12	-	PCBP2	NM_005016	Intron
1	MDM	6	71465814	71465890	chr14	+	PCNX	NM_014982	Intron
1	MDM	1	70504058	70504148	chr2	+	PCYOX1	NM_016297	Exon
1	CD4 T	1	112642248	112642339	chr10	+	PDCD4	NM_001199492	Intron
1	MDM	1	64363436	64363539	chr2	+	PELI1	NM_020651	Intron
1	CD4 T	JR-FL	76390285	76390474	chr17	-	PGS1	NR_110602	Intron
1	MDM	6	38442247	38442317	chr21	-	PIGP	NM_153681	Intron
1	MDM	JR-FL	145021964	145022013	chr8	+	PLEC	NM_201380	Intron
1	CD4 T	1	145037612	145037649	chr8	+	PLEC	NM_201378	Intron
1	MDM	6	154904694	154904739	chr1	+	PMVK	NM_006556	Intron
1	MDM	6	7614998	7615156	chr19	-	PNPLA6	NM_006702	Exon
1	MDM	6	65039318	65039400	chr11	+	POLA2	NM_002689	Intron
1	MDM	1	31136352	31136481	chr6	+	POU5F1	NM_002701	Intron

1	CD4 T	1	46557227	46557273	chr22	+	PPARA	NM_001001928	Intron
1	MDM	1	30580707	30580780	chr6	-	PPP1R10	NM_002714	Intron
1	MDM	JR-FL	104216215	104216267	chr14	+	PPP1R13B	NM_015316	Exon
1	MDM	6	131891582	131891606	chr9	-	PPP2R4	NM_021131	Intron
1	MDM	JR-FL	42955760	42955804	chr6	-	PPP2R5D	NM_006245	Intron
1	CD4 T	1	73102075	73102285	chr3	+	PPP4R2	NM_174907	Intron
1	MDM	6	55744860	55744952	chr19	+	PPP6R1	NM_014931	Intron
1	MDM	6	50804554	50804593	chr22	-	PPP6R2	NM_001242898	Intron
1	CD4 T	1	23888420	23888486	chr16	+	PRKCB	NM_002738	Intron
1	MDM	JR-FL	74334350	74334479	chr17	+	PRPSAP1	NM_002766	Intron
1	MDM	JR-FL	50122524	50122658	chr19	-	PRR12	NM_020719	Intron
1	MDM	6	171540496	171540535	chr1	+	PRRC2C	NM_015172	Exon
1	CD4 T	1	113955652	113955760	chr2	+	PSD4	NM_012455	Intron
1	MDM	6	87067859	87067957	chr8	-	PSKH2	NM_033126	Intron
1	CD4 T	1	109960084	109960211	chr1	+	PSMA5	NM_001199772	Intron
1	MDM	JR-FL	232001458	232001495	chr2	-	PSMD1	NM_001191037	Intron
1	CD4 T	1	65344311	65344367	chr17	+	PSMD12	NM_174871	Intron
1	MDM	6	237909	237989	chr11	-	PSMD13	NM_175932	Intron
1	CD4 T	JR-FL	800308	800402	chr19	+	PTBP1	NM_031991	Intron
1	MDM	6	57066783	57066962	chr12	-	PTGES3	NR_104219	Undetermined
1	MDM	JR-FL	123265096	123265210	chr3	+	PTPLB	NM_198402	Intron
1	MDM	6	7066648	7066678	chr12	-	PTPN6	NM_080548	Intron
1	MDM	6	2888354	2888403	chr20	+	PTPRA	NM_002836	Intron
1	CD4 T	1	8072604	8072628	chr18	-	PTPRM	NM_002845	Intron
1	MDM	JR-FL	58395569	58395689	chr3	+	PXK	NM_017771	Intron
1	MDM	6	51399419	51399442	chr14	-	PYGL	NM_001163940	Intron
1	MDM	1	32975599	32975656	chr11	+	QSER1	NM_001076786	Exon
1	CD4 T	1	119768545	119768682	chr10	+	RAB11FIP2	NM_014904	Exon
1	MDM	1	28931510	28931533	chr16	-	RABEP2	NM_024816	Intron
1	MDM	6	139709768	139709840	chr9	-	RABL6	NM_001173989	Intron
1	MDM	6	51605674	51605743	chr3	-	RAD54L2	NM_015106	Intron
1	CD4 T	JR-FL	131511708	131511746	chrX	+	RAP2C-AS1	NR_110410	Undetermined
1	MDM	6	134549573	134549602	chr9	-	RAPGEF1	NM_198679	Intron
1	CD4 T	1	38850357	38850381	chr15	+	RASGRP1	NM_005739	Intron
1	MDM	JR-FL	205083906	205083926	chr1	+	RBBP5	NM_005057	Undetermined
1	MDM	6	34317328	34317406	chr20	-	RBM39	NM_004902	Intron
1	MDM	1	50046448	50046516	chr19	-	RCN3	NM_020650	Exon
1	MDM	JR-FL	111698355	111698408	chr6	-	REV3L	NM_001286431	Intron
1	MDM	6	176090316	176090357	chr1	-	RFWD2	NM_022457	Intron
1	MDM	1	74474689	74474828	chr17	+	RHBDF2	NM_001005498	Intron
1	MDM	6	40229820	40229853	chr4	+	RHOH	NM_001278368	Intron
1	CD4 T	1	74216475	74216504	chr17	+	RNF157	NM_052916	Intron
1	MDM	1	74481431	74481522	chr11	-	RNF169	NM_001098638	Intron
1	MDM	6	3974999	3975050	chr20	-	RNF24	NM_007219	Intron
1	CD4 T	1	24625227	24625303	chr14	-	RNF31	NM_017999	Intron
1	MDM	JR-FL	104077689	104077743	chr1	-	RNPC3	NM_017619	Intron
1	MDM	JR-FL	2311817	2311848	chr16	-	RNPS1	NM_001286625	Intron
1	MDM	6	78963101	78963193	chr3	+	ROBO1	NM_133631	Intron
1	CD4 T	JR-FL	92823394	92823548	chr1	-	RPAP2	NM_024813	Intron
1	MDM	6	38166601	38166625	chrX	+	RPGR	NM_000328	Intron
1	MDM	JR-FL	89631426	89631464	chr16	+	RPL13	NM_000977	3´UTR
1	MDM	6	128356797	128356882	chr3	-	RPN1	NM_002950	Exon
1	MDM	1	78830929	78830961	chr17	+	RPTOR	NM_001163034	Intron
1	MDM	6	63486887	63487005	chr11	+	RTN3	NM_201429	Undetermined
1	MDM	6	46542374	46542400	chr3	+	RTP3	NM_031440	3´UTR
1	MDM	6	137267495	137267522	chr9	-	RXRA	NM_002957	Intron

1	MDM	6	33300140	33300291	chr1	-	S100PBP	NM_001256121	Intron
1	MDM	6	47657672	47657741	chr19	-	SAE1	NM_001145713	Intron
1	MDM	6	47638607	47638712	chr19	-	SAE1	NM_005500	Intron
1	CD4 T	1	5639085	5639175	chr19	-	SAFB	NR_037699	Intron
1	MDM	6	5605005	5605073	chr19	-	SAFB2	NM_014649	Intron
1	CD4 T	1	44380073	44380099	chr22	+	SAMM50	NM_015380	Intron
1	MDM	JR-FL	26705185	26705235	chr17	-	SARM1	NM_015077	Intron
1	MDM	JR-FL	65736486	65736509	chr11	-	SART1	NM_005146	Intron
1	MDM	JR-FL	10145679	10145715	chr11	-	SBF2	NM_030962	Intron
1	MDM	6	1127354	1127473	chr19	+	SBNO2	NM_001100122	Intron
1	MDM	1	1140311	1140435	chr19	+	SBNO2	NM_014963	Intron
1	MDM	JR-FL	7481032	7481058	chr17	+	SENP3-EIF4A1	NR_037926	Intron
1	CD4 T	1	101115185	101115279	chr3	-	SENP7	NM_001282803	Intron
1	MDM	1	18031376	18031512	chr11	-	SERGEF	NM_012139	Intron
1	MDM	6	180102694	180102743	chr2	+	SESTD1	NM_178123	Intron
1	MDM	JR-FL	47084114	47084156	chr3	-	SETD2	NM_014159	Exon
1	MDM	6	111878135	111878241	chr12	+	SH2B3	NM_005475	Intron
1	CD4 T	1	46475262	46475377	chr17	+	SKAP1	NM_003726	Intron
1	CD4 T	1	78321745	78321785	chr13	+	SLAIN1	NM_001242868	Intron
1	CD4 T	1	127510576	127510669	chr5	+	SLC12A2	NM_001046	Intron
1	CD4 T	1	172739405	172739515	chr2	+	SLC25A12	NM_003705	Intron
1	MDM	6	73116098	73116124	chr10	-	SLC29A3	NR_033413	Intron
1	MDM	6	92894620	92894663	chr11	+	SLC36A4	NM_001286139	Intron
1	MDM	JR-FL	46582673	46582725	chr12	+	SLC38A1	NM_001278389	3´UTR
1	MDM	1	49801367	49801392	chr19	+	SLC6A16	NM_014037	Intron
1	MDM	6	30109041	30109092	chr13	-	SLC7A1	NM_003045	Intron
1	MDM	1	11160538	11160559	chr19	+	SMARCA4	NM_003072	Intron
1	MDM	6	18820776	18820816	chr16	-	SMG1	NM_015092	3´UTR
1	MDM	6	33051199	33051358	chr9	+	SMU1	NM_018225	Intron
1	MDM	6	1703550	1703682	chr17	-	SMYD4	NM_052928	Exon
1	MDM	1	1696648	1696690	chr17	-	SMYD4	NM_052928	Intron
1	MDM	1	127338928	127339020	chr7	-	SND1	NM_014390	Exon
1	CD4 T	JR-FL	41258352	41258392	chr19	-	SNRPA	NM_004596	Intron
1	CD4 T	1	151628809	151628874	chr1	-	SNX27	NM_030918	Intron
1	CD4 T	1	17933785	17933960	chr20	-	SNX5	NM_014426	Intron
1	MDM	6	231200599	231200735	chr2	+	SP140L	NM_138402	Intron
1	CD4 T	1	49060144	49060174	chr17	-	SPAG9	NM_001251971	Intron
1	CD4 T	1	35622369	35622424	chr5	-	SPEF2	NM_144722	Intron
1	MDM	JR-FL	9377430	9377508	chr1	-	SPSB1	NM_025106	Intron
1	CD4 T	1	74053828	74053935	chr17	-	SRP68	NM_014230	Intron
1	MDM	6	28075867	28075995	chr17	-	SSH2	NM_033389	Intron
1	MDM	JR-FL	67075313	67075433	chr11	-	SSH3	NM_017857	Intron
1	MDM	6	1498403	1498526	chr1	+	SSU72	NM_014188	Intron
1	MDM	6	1490350	1490443	chr1	+	SSU72	NM_014188	Intron
1	MDM	6	76722787	76722913	chr1	+	ST6GALNAC3	NM_001160011	Intron
1	MDM	JR-FL	40353193	40353232	chr17	-	STAT5B	NM_012448	3´UTR
1	MDM	6	47801029	47801082	chr20	-	STAU1	NM_001037328	Intron
1	MDM	JR-FL	36845374	36845524	chr1	+	STK40	NM_032017	Intron
1	MDM	JR-FL	61802273	61802351	chr17	+	STRADA	NM_001003786	Intron
1	MDM	6	31465475	31465611	chr14	-	STRN3	NM_001083893	Intron
1	CD4 T	1	46345816	46345897	chr19	+	SYMPK	NM_004819	Intron
1	CD4 T	1	68903531	68903623	chr16	-	TANGO6	NM_024562	Intron
1	MDM	JR-FL	32794945	32795010	chr6	+	TAP2	NM_018833	Undetermined
1	CD4 T	1	72286101	72286198	chr12	-	TBC1D15	NM_001146213	Intron
1	MDM	6	37276515	37276567	chr6	-	TBC1D22B	NM_017772	Intron
1	MDM	1	176757079	176757165	chr3	-	TBL1XR1	NM_024665	Intron

1	MDM	1	24652992	24653052	chr6	-	TDP2	NM_016614	Intron
1	CD4 T	JR-FL	43801315	43801353	chr2	-	THADA	NM_001083953	Intron
1	MDM	6	72066577	72066633	chr12	-	THAP2	NM_031435	Intron
1	MDM	1	121342963	121343008	chr10	+	TIAL1	NM_003252	Intron
1	MDM	1	201932846	201932932	chr1	+	TIMM17A	NM_006335	Intron
1	MDM	6	50140143	50140246	chr12	+	TMBIM6	NM_003217	Intron
1	CD4 T	1	109301629	109301720	chrX	-	TMEM164	NM_017698	Intron
1	MDM	6	112406864	112406927	chr7	+	TMEM168	NM_022484	3´UTR
1	MDM	JR-FL	4650316	4650391	chr19	-	TNFAIP8L1	NM_152362	Intron
1	MDM	6	72193831	72193882	chr5	-	TNPO1	NM_002270	Intron
1	MDM	6	12822010	12822138	chr19	+	TNPO2	NM_013433	Exon
1	CD4 T	1	24820121	24820186	chr16	+	TNRC6A	NM_014494	Intron
1	CD4 T	1	76025652	76025695	chr17	+	TNRC6C	NM_018996	Intron
1	CD4 T	1	39706419	39706509	chr20	+	TOP1	NM_003286	Intron
1	CD4 T	JR-FL	21950759	21950809	chr14	+	TOX4	NM_014828	Intron
1	MDM	JR-FL	103266750	103266815	chr13	-	TPP2	NM_003291	Intron
1	CD4 T	1	30349906	30350066	chr20	+	TPX2	NM_012112	Intron
1	MDM	1	50855861	50855962	chr15	+	TRPM7	NM_017672	Intron
1	MDM	1	231700322	231700369	chr1	-	TSNAX	NM_005999	Undetermined
1	MDM	6	28679028	28679119	chr22	-	TTC28	NM_001145418	Intron
1	MDM	JR-FL	21616671	21616691	chr18	+	TTC39C	NM_153211	Intron
1	CD4 T	1	135271224	135271366	chr9	+	TTF1	NM_001205296	Intron
1	MDM	6	18686113	18686186	chr19	-	UBA52	NM_001033930	3´UTR
1	CD4 T	1	122602050	122602248	chr11	+	UBASH3B	NM_032873	Intron
1	MDM	JR-FL	4224060	4224132	chr17	+	UBE2G1	NM_003342	Intron
1	MDM	JR-FL	1362705	1362783	chr16	-	UBE2I	NM_194261	Intron
1	MDM	6	76157521	76157559	chr15	+	UBE2Q2	NM_173469	Intron
1	CD4 T	1	165875658	165875787	chr1	-	UCK2	NM_012474	Intron
1	CD4 T	1	1460860	1461041	chr16	+	UNKL	NM_001193388	Intron
1	MDM	6	30402474	30402547	chr21	+	USP16	NM_006447	Intron
1	MDM	6	58455165	58455229	chr17	+	USP32	NM_032582	Intron
1	MDM	6	11934077	11934134	chr11	+	USP47	NM_001282659	Intron
1	MDM	JR-FL	120206094	120206199	chr4	-	USP53	NM_019050	Intron
1	MDM	JR-FL	50720991	50721105	chr15	+	USP8	NM_001128610	Intron
1	MDM	JR-FL	41026448	41026491	chrX	-	USP9X	NM_001039591	Intron
1	MDM	6	108129380	108129401	chr1	-	VAV3	NM_001079874	Intron
1	MDM	6	82828332	82828405	chr5	+	VCAN	NM_004385	Intron
1	CD4 T	1	100548447	100548533	chr8	+	VPS13B	NM_152564	Intron
1	CD4 T	1	196282181	196282224	chr3	+	WDR53	NM_182627	Intron
1	MDM	6	702678	702780	chr16	-	WDR90	NM_145294	Intron
1	MDM	6	707946	707981	chr16	-	WDR90	NM_145294	Intron
1	MDM	6	948589	948728	chr12	-	WNK1	NM_213655	Intron
1	MDM	6	21465635	21465690	chr13	-	XPO4	NM_022459	Intron
1	MDM	1	47711141	47711208	chr21	-	YBEY	NM_001006114	Intron
1	MDM	1	1259000	1259048	chr17	+	YWHAE	NM_006761	Intron
1	MDM	JR-FL	50258383	50258464	chr22	+	ZBED4	NM_014838	Intron
1	MDM	JR-FL	129584423	129584456	chr9	-	ZBTB43	NM_014007	Intron
1	CD4 T	1	129591233	129591432	chr9	-	ZBTB43	NM_001135776	Intron
1	CD4 T	1	113063144	113063281	chr2	-	ZC3H6	NM_198581	Intron
1	MDM	JR-FL	58350084	58350157	chr11	-	ZFP91	NM_053023	Intron
1	MDM	6	124264596	124264627	chr8	+	ZHX1-C8orf76	NM_001204180	Intron
1	MDM	6	99107485	99107574	chr7	-	ZKSCAN5	NM_145102	Intron
1	MDM	JR-FL	133715382	133715430	chr12	-	ZNF10	NM_015394	Intron
1	MDM	6	53595288	53595341	chr19	+	ZNF160	NM_198893	Intron
1	CD4 T	1	3274443	3274506	chr16	+	ZNF200	NM_003454	Exon
1	MDM	6	44459823	44459913	chr19	-	ZNF221	NM_013359	Intron

1	MDM	JR-FL	146116477	146116516	chr8	-	ZNF250	NM_001109689	Intron
1	MDM	1	6778117	6778161	chr12	-	ZNF384	NM_133476	Intron
1	MDM	6	12713469	12713609	chr19	-	ZNF490	NM_020714	Intron
1	MDM	JR-FL	12658100	12658132	chr19	+	ZNF564	NM_144976	Intron
1	MDM	JR-FL	2051806	2051834	chr16	+	ZNF598	NM_178167	Intron
1	MDM	JR-FL	53479142	53479165	chr19	+	ZNF702P	NR_003578	Intron
1	MDM	JR-FL	12079744	12079857	chr19	-	ZNF763	NM_001012753	Intron
1	MDM	JR-FL	100867077	100867108	chr7	+	ZNHIT1	NM_006349	Exon
1	MDM	JR-FL	3996798	3996914	chr17	+	ZZEF1	NM_015113	Intron
1	CD4 T	JR-FL	43818315	43818531	chr21	+			Intergenic
1	CD4 T	JR-FL	74019554	74019589	chr10	-			Intergenic
1	CD4 T	JR-FL	151934569	151934659	chr1	+			Intergenic
1	CD4 T	JR-FL	59057770	59057940	chr16	-			Intergenic
1	CD4 T	JR-FL	55926396	55926490	chr5	-			Intergenic
1	MDM	1	56432664	56432705	chr12	+			Intergenic
1	MDM	1	34377796	34377825	chr17	+			Intergenic
1	MDM	1	158254128	158254167	chr1	+			Intergenic
1	MDM	1	20847410	20847526	chr18	-			Intergenic
1	MDM	1	150307846	150307898	chr7	+			Intergenic
1	MDM	1	61762442	61762510	chr11	-			Intergenic
1	MDM	1	61216102	61216142	chr11	-			Intergenic
1	MDM	1	100003436	100003474	chrX	+			Intergenic
1	MDM	1	46459916	46459947	chr22	-			Intergenic
1	MDM	1	54857001	54857022	chr6	-			Intergenic
1	MDM	1	65229487	65229545	chr11	+			Intergenic
1	MDM	6	151891515	151891595	chr1	+			Intergenic
1	MDM	6	24800489	24800564	chr6	+			Intergenic
1	MDM	6	134071587	134071679	chr7	-			Intergenic
1	MDM	6	92100444	92100532	chr8	-			Intergenic
1	MDM	6	48040470	48040530	chr21	-			Intergenic
1	MDM	6	100834554	100834690	chr7	+			Intergenic
1	MDM	6	68317343	68317367	chr8	-			Intergenic
1	MDM	6	2495800	2495832	chr1	+			Intergenic
1	MDM	6	43845668	43845835	chr1	-			Intergenic
1	MDM	6	69537847	69537894	chr10	+			Intergenic
1	MDM	6	22318376	22318503	chr12	+			Intergenic
1	MDM	6	24864606	24864708	chr12	+			Intergenic
1	MDM	6	66106043	66106096	chr15	+			Intergenic
1	MDM	6	73163495	73163536	chr17	+			Intergenic
1	MDM	6	74115909	74115931	chr17	-			Intergenic
1	MDM	6	47887497	47887557	chr19	-			Intergenic
1	MDM	6	150828636	150828794	chr4	-			Intergenic
1	MDM	6	8186075	8186125	chr4	-			Intergenic
1	MDM	6	42470401	42470425	chr6	-			Intergenic
1	MDM	6	139763406	139763710	chr9	-			Intergenic
1	MDM	6	139627927	139627977	chr9	-			Intergenic
1	MDM	6	100138524	100138575	chr5	-			Intergenic
1	MDM	6	85690439	85690481	chr2	-			Intergenic
1	MDM	6	22528642	22528751	chr8	-			Intergenic
1	MDM	6	79343940	79343963	chr17	+			Intergenic
1	MDM	6	161511812	161511849	chr2	+			Intergenic
1	MDM	6	208200147	208200312	chr2	+			Intergenic
1	MDM	6	129139527	129139565	chr8	-			Intergenic
1	MDM	6	7824209	7824340	chr17	-			Intergenic
1	MDM	6	80246755	80246828	chr17	+			Intergenic
1	MDM	6	152656938	152657095	chr2	+			Intergenic

1	MDM	6	22727032	22727075	chr12	-			Intergenic
1	MDM	6	27397408	27397493	chr16	+			Intergenic
1	MDM	6	17848815	17848861	chr19	-			Intergenic
1	MDM	6	58998666	58998704	chr11	-			Intergenic
1	MDM	6	54852977	54853159	chr19	-			Intergenic
1	MDM	6	89622131	89622151	chr12	-			Intergenic
1	MDM	6	8072128	8072156	chr17	-			Intergenic
1	MDM	6	32407385	32407498	chr6	-			Intergenic
1	MDM	6	24061858	24061882	chr1	-			Intergenic
1	MDM	6	60887591	60887641	chr16	+			Intergenic
1	MDM	6	152997569	152997591	chr3	-			Intergenic
1	MDM	6	140016878	140016905	chr5	+			Intergenic
1	MDM	6	118822545	118822575	chr4	+			Intergenic
1	MDM	6	35132389	35132484	chr14	+			Intergenic
1	MDM	JR-FL	40890238	40890288	chr1	+			Intergenic
1	MDM	JR-FL	58930104	58930148	chr19	-			Intergenic
1	MDM	JR-FL	31264071	31264145	chr6	+			Intergenic
1	MDM	JR-FL	64926686	64926746	chr11	-			Intergenic
1	MDM	JR-FL	74916416	74916463	chr4	+			Intergenic
1	MDM	JR-FL	41149529	41149573	chr17	+			Intergenic
1	MDM	JR-FL	150866660	150866834	chr1	+			Intergenic
1	MDM	JR-FL	30551367	30551449	chr16	+			Intergenic
1	MDM	JR-FL	85992260	85992305	chr16	-			Intergenic
1	MDM	JR-FL	5001192	5001254	chr10	+			Intergenic
1	MDM	JR-FL	65407966	65408095	chr2	+			Intergenic
1	MDM	JR-FL	204533101	204533232	chr2	+			Intergenic
1	MDM	JR-FL	55772990	55773030	chr19	-			Intergenic
1	MDM	JR-FL	1189755	1189802	chr19	-			Intergenic
1	MDM	JR-FL	23280954	23281009	chr7	-			Intergenic
1	MDM	JR-FL	10215040	10215067	chr19	-			Intergenic
1	MDM	JR-FL	43143702	43143735	chr14	+			Intergenic
1	MDM	JR-FL	80059073	80059165	chr17	-			Intergenic
1	MDM	JR-FL	2897225	2897267	chr16	+			Intergenic
1	MDM	JR-FL	187280303	187280330	chr2	+			Intergenic
1	MDM	JR-FL	110877191	110877212	chr11	+			Intergenic
1	MDM	JR-FL	59490644	59490708	chr11	+			Intergenic
1	MDM	JR-FL	120547576	120547649	chr10	-			Intergenic
1	MDM	JR-FL	53537475	53537496	chr12	+			Intergenic
1	CD4 T	1	29359624	29359660	chr8	-			Intergenic
1	CD4 T	1	29105334	29105354	chr17	-			Intergenic
1	CD4 T	1	30468598	30468728	chr16	-			Intergenic
1	CD4 T	1	7424962	7425031	chr17	+			Intergenic
1	CD4 T	1	83837199	83837320	chr1	+			Intergenic
1	CD4 T	1	44778828	44778870	chr7	+			Intergenic
1	CD4 T	1	156686986	156687126	chr1	+			Intergenic
1	CD4 T	1	17363356	17363503	chr2	-			Intergenic
1	CD4 T	1	150187072	150187219	chr7	+			Intergenic
1	CD4 T	1	69722574	69722669	chr12	-			Intergenic
1	CD4 T	1	44447180	44447323	chr20	+			Intergenic
1	CD4 T	1	31327106	31327182	chr6	+			Intergenic
1	CD4 T	1	96058003	96058071	chr6	-			Intergenic
1	CD4 T	1	24727879	24727964	chr2	-			Intergenic
2	CD4 T	JR-FL	105966964	105967030	chr11	+	AASDHPPT	NM_015423	Intron
2	CD4 T	2	41869737	41869912	chr22	-	ACO2	NM_001098	Intron
2	CD4 T	JR-FL	44483853	44483912	chr20	-	ACOT8	NM_005469	Exon
2	MDM	2	46541619	46541671	chr21	+	ADARB1	NM_001112	Intron

2	CD4 T	2	329873	329961	chr5	+	AHRR	NM_001242412	Intron
2	MDM	JR-FL	105257971	105258053	chr14	+	AKT1	NM_005163	Intron
2	CD4 T	2	133329285	133329339	chr12	+	ANKLE2	NM_015114	Intron
2	MDM	2	69082905	69083069	chr15	-	ANP32A	NM_006305	Intron
2	MDM	JR-FL	2101420	2101454	chr19	-	AP3D1	NM_003938	3´UTR
2	MDM	JR-FL	79826274	79826322	chr17	+	ARHGDIA	NM_001185077	Undetermined
2	MDM	1	48986434	48986482	chr3	+	ARIH2	NM_006321	Intron
2	CD4 T	2	150838845	150838968	chr1	-	ARNT	NM_001286035	Intron
2	CD4 T	2	75035032	75035191	chr11	+	ARRB1	NM_020251	Intron
2	CD4 T	JR-FL	197070734	197070806	chr1	-	ASPM	NM_001206846	Undetermined
2	CD4 T	2	47127319	47127392	chr1	+	ATPAF1	NM_001256418	Intron
2	MDM	2	392937	393242	chr16	+	AXIN1	NM_181050	Intron
2	CD4 T	2	91327020	91327162	chr15	+	BLM	NM_001287248	Intron
2	CD4 T	2	59964424	59964469	chr15	+	BNIP2	NM_004330	Intron
2	MDM	2	28213929	28213967	chr2	-	BRE	NM_199192	Intron
2	MDM	2	93773631	93773952	chr14	+	BTBD7	NM_001289133	Intron
2	CD4 T	2	124917492	124917526	chr10	-	BUB3	NM_001007793	Intron
2	CD4 T	2	43834931	43834975	chr18	+	C18orf25	NM_001008239	Intron
2	MDM	JR-FL	138724665	138724695	chr9	+	CAMSAP1	NM_015447	Intron
2	CD4 T	2	6886377	6886500	chr1	+	CAMTA1	NM_001195563	Intron
2	MDM	2	54652655	54652831	chr12	+	CBX5	NM_001127322	Intron
2	CD4 T	2	109416054	109416094	chr2	-	CCDC138	NM_144978	Intron
2	MDM	JR-FL	111310289	111310381	chr12	-	CCDC63	NM_001286243	Intron
2	CD4 T	2	45219410	45219484	chr17	-	CDC27	NM_001256	Intron
2	CD4 T	2	88872352	88872507	chr16	-	CDT1	NM_030928	Intron
2	CD4 T	2	68395719	68395794	chr4	+	CENPC	NM_001812	Intron
2	MDM	2	95112813	95112849	chr9	+	CENPP	NM_001286969	Intron
2	MDM	2	89713046	89713098	chr16	+	CHMP1A	NR_046418	Undetermined
2	MDM	2	46839451	46839506	chr11	-	CKAP5	NM_014756	Intron
2	CD4 T	2	43675082	43675134	chr7	-	COA1	NR_047701	Intron
2	MDM	1	48608744	48608883	chr3	-	COL7A1	NM_000094	Intron
2	CD4 T	2	150717459	150717637	chr1	-	CTSS	NM_004079	Intron
2	MDM	2	107888882	107889014	chr11	+	CUL5	NM_003478	Intron
2	MDM	2	50898314	50898351	chr18	-	DCC	NM_005215	Intron
2	CD4 T	2	118656500	118656632	chr11	+	DDX6	NM_001257191	Intron
2	MDM	JR-FL	47935807	47935884	chr21	-	DIP2A	NM_206891	Intron
2	MDM	2	62547928	62548016	chr20	+	DNAJC5	NM_025219	Intron
2	CD4 T	2	225734224	225734386	chr2	+	DOCK10	NM_001290263	Intron
2	CD4 T	2	140452727	140452771	chr9	-	DPH7	NM_138778	Intron
2	MDM	2	26634630	26634659	chr2	+	DRC1	NM_145038	Intron
2	MDM	2	21568429	21568518	chr1	+	ECE1	NM_001113347	Intron
2	CD4 T	2	103803115	103803149	chr14	-	EIF5	NM_001969	Exon
2	CD4 T	JR-FL	13755222	13755306	chr20	+	ESF1	NM_016649	Intron
2	MDM	JR-FL	145135439	145135459	chr8	-	EXOSC4	NM_019037	Exon
2	CD4 T	2	96232337	96232628	chr9	-	FAM120A	NM_001286724	Intron
2	MDM	2	73270291	73270454	chr11	+	FAM168A	NM_001286050	Intron
2	CD4 T	JR-FL	102679860	102680031	chr10	+	FAM178A	NM_001136123	Intron
2	CD4 T	JR-FL	88915467	88915578	chr10	+	FAM35A	NM_019054	Intron
2	MDM	2	242302477	242302527	chr2	-	FARP2	NM_001282984	Intron
2	CD4 T	2	35623010	35623143	chr6	+	FKBP5	NM_001145776	Intron
2	CD4 T	2	50096822	50096921	chr12	+	FMNL3	NM_198900	Intron
2	MDM	2	120894362	120894554	chr12	+	GATC	NR_033684	Intron
2	MDM	2	104059562	104059697	chr10	+	GBF1	NM_001199379	Intron
2	CD4 T	2	92741412	92741480	chr1	-	GLMN	NM_053274	Intron
2	MDM	2	37401438	37401671	chr3	-	GOLGA4	NM_001172713	Intron
2	CD4 T	2	5558945	5559022	chr20	+	GPCPD1	NM_019593	Intron

2	CD4 T	2	153226353	153226407	chrX	-	HCFC1	NM_005334	Intron
2	MDM	JR-FL	1746621	1746687	chr16	+	HN1L	NM_144570	Intron
2	CD4 T	2	5449251	5449303	chr5	+	ICE1	NM_015325	Intron
2	MDM	2	18489474	18489519	chr1	-	IGSF21	NM_032880	Intron
2	CD4 T	2	143280519	143280662	chr4	-	INPP4B	NM_001101669	Intron
2	CD4 T	2	90972849	90972896	chr15	-	IQGAP1	NM_003870	Exon
2	CD4 T	2	78767580	78767647	chr15	+	IREB2	NM_004136	Intron
2	MDM	2	173369485	173369633	chr2	-	ITGA6	NM_000210	3´UTR
2	MDM	2	48825532	48825667	chr13	-	ITM2B	NM_021999	Intron
2	MDM	JR-FL	44230205	44230248	chr17	-	KANSL1	NM_001193466	Intron
2	MDM	2	36429228	36429303	chr6	-	KCTD20	NR_104481	Intron
2	CD4 T	2	7751248	7751320	chr17	-	KDM6B	NM_001080424	Exon
2	CD4 T	JR-FL	6500787	6500813	chr17	+	KIAA0753	NM_014804	Intron
2	MDM	2	115256294	115256344	chr9	+	KIAA1958	NM_133465	Intron
2	MDM	JR-FL	241721320	241721357	chr1	+	KMO	NM_003679	Intron
2	CD4 T	2	151968157	151968223	chr7	-	KMT2C	NM_170606	Intron
2	MDM	2	96300269	96300291	chr5	-	LNPEP	NM_005575	Intron
2	CD4 T	JR-FL	68840548	68840688	chr12	+	LOC100507195	NR_120458	Intron
2	CD4 T	JR-FL	76257744	76257873	chr17	+	LOC100996291	NR_073178	Undetermined
2	CD4 T	2	86050184	86050237	chr8	-	LRRCC1	NM_033402	Intron
2	MDM	2	270896	270932	chr16	+	LUC7L	NM_018032	Intron
2	CD4 T	JR-FL	4104957	4104986	chr19	-	MAP2K2	NM_030662	Intron
2	MDM	2	39617320	39617361	chr2	+	MAP4K3	NM_003618	Intron
2	MDM	2	22180296	22180500	chr22	+	MAPK1	NM_002745	Intron
2	MDM	2	36022685	36022846	chr6	-	MAPK14	NM_139013	Intron
2	CD4 T	JR-FL	66321513	66321552	chr5	-	MAST4	NM_001164664	Intron
2	CD4 T	2	60075265	60075365	chr17	-	MED13	NM_005121	Intron
2	CD4 T	2	116533776	116533961	chr12	+	MED13L	NM_015335	Intron
2	CD4 T	2	21657867	21658172	chr16	+	METTL9	NM_001288659	Intron
2	CD4 T	2	12053314	12053395	chr1	+	MFN2	NM_014874	Intron
2	MDM	JR-FL	74250178	74250274	chr10	-	MIR1256	NR_031657	Intron
2	MDM	2	198866257	198866368	chr1	+	MIR181A1HG	NR_040073	Intron
2	MDM	2	122585590	122585779	chr12	+	MLXIP	NM_014938	Intron
2	MDM	2	123713262	123713318	chr12	-	MPHOSPH9	NM_022782	Intron
2	CD4 T	2	105907271	105907324	chr14	+	MTA1	NM_001203258	Intron
2	CD4 T	2	112503664	112503690	chr12	-	NAA25	NM_024953	Intron
2	MDM	JR-FL	105899585	105899685	chr7	-	NAMPT	NM_005746	Intron
2	CD4 T	2	6609870	6609952	chr12	+	NCAPD2	NM_014865	Intron
2	CD4 T	2	46204051	46204092	chr20	+	NCOA3	NM_181659	Intron
2	CD4 T	2	140107579	140107651	chr9	-	NDOR1	NM_001144027	Intron
2	CD4 T	2	198145029	198145275	chr1	+	NEK7	NM_133494	Intron
2	CD4 T	2	30028944	30029104	chr22	-	NF2	NM_181825	Intron
2	MDM	JR-FL	27659100	27659132	chr2	-	NRBP1	NM_013392	Intron
2	CD4 T	2	176665666	176665932	chr5	-	NSD1	NM_172349	Intron
2	MDM	2	44520073	44520129	chr7	+	NUDCD3	NM_015332	Intron
2	MDM	2	65918213	65918276	chr11	-	PACS1	NM_018026	Intron
2	MDM	2	65936018	65936152	chr11	+	PACS1	NM_018026	Intron
2	MDM	2	53848214	53848234	chr12	-	PCBP2	NM_001128911	Intron
2	MDM	2	47292852	47292889	chr21	+	PCBP3	NM_001130141	Intron
2	CD4 T	2	85679536	85679673	chr11	+	PICALM	NM_007166	Intron
2	CD4 T	JR-FL	208800615	208800658	chr2	-	PLEKHM3	NM_001080475	Intron
2	MDM	1	140435470	140435511	chr9	-	PNPLA7	NM_001098537	Intron
2	MDM	2	133206169	133206258	chr12	+	POLE	NM_006231	Intron
2	MDM	2	68339185	68339288	chr11	-	PPP6R3	NM_001164161	Intron
2	CD4 T	2	14030836	14030903	chr1	+	PRDM2	NM_001135610	Intron
2	CD4 T	JR-FL	14063990	14064025	chr1	-	PRDM2	NM_001135610	Intron

2	CD4 T	2	31452314	31452432	chr1	+	PUM1	NM_014676	Intron
2	CD4 T	2	139133317	139133353	chr9	+	QSOX2	NM_181701	Intron
2	MDM	2	153954812	153954963	chr1	+	RAB13	NM_001272038	Intron
2	MDM	JR-FL	9831222	9831363	chr18	-	RAB31	NM_006868	Intron
2	CD4 T	2	206731540	206731689	chr1	+	RASSF5	NM_182664	Intron
2	MDM	2	6096811	6096863	chr19	+	RFX2	NM_000635	Intron
2	CD4 T	2	41044072	41044168	chr15	-	RMDN3	NM_018145	Intron
2	CD4 T	2	59345290	59345465	chr15	+	RNF111	NM_017610	Intron
2	MDM	JR-FL	2497827	2497856	chr4	+	RNF4	NM_002938	Intron
2	MDM	2	1995561	1995656	chr16	+	RPL3L	NM_005061	Intron
2	MDM	2	78644159	78644214	chr17	+	RPTOR	NM_001163034	Intron
2	CD4 T	2	77392262	77392333	chr7	+	RSBN1L	NM_198467	Intron
2	CD4 T	2	123005633	123005669	chr12	-	RSRC2	NM_023012	Intron
2	CD4 T	2	45764109	45764143	chr3	+	SACM1L	NM_014016	Intron
2	MDM	JR-FL	56157585	56157688	chr12	+	SARNP	NR_026722	Intron
2	MDM	2	50154062	50154166	chr19	-	SCAF1	NM_021228	Intron
2	CD4 T	JR-FL	110420429	110420509	chr4	+	SEC24B	NM_001042734	Intron
2	CD4 T	2	78203630	78203724	chr17	-	SLC26A11	NM_173626	Intron
2	MDM	2	115996983	115997215	chr9	+	SLC31A1	NM_001859	Intron
2	CD4 T	2	38800279	38800326	chr17	+	SMARCE1	NM_003079	Intron
2	CD4 T	2	151637552	151637720	chr1	-	SNX27	NM_030918	Intron
2	MDM	JR-FL	24837304	24837325	chr13	-	SPATA13	NM_001286792	Intron
2	CD4 T	2	121287559	121287683	chr12	+	SPPL3	NM_139015	Intron
2	CD4 T	2	65452326	65452413	chr5	-	SREK1	NM_001270493	Undetermined
2	CD4 T	2	41247171	41247345	chr22	+	ST13	NM_001278589	Intron
2	MDM	2	3991974	3992175	chr11	+	STIM1	NM_001277962	Intron
2	CD4 T	2	43616571	43616735	chr20	-	STK4	NM_006282	Intron
2	MDM	2	48555122	48555226	chr13	-	SUCLA2	NM_003850	Intron
2	CD4 T	2	64448765	64448941	chr14	+	SYNE2	NM_015180	Intron
2	MDM	JR-FL	44020152	44020197	chr20	+	SYS1-DBNDD2	NR_003189	Intron
2	MDM	2	27860114	27860210	chr7	+	TAX1BP1	NM_001206901	Intron
2	MDM	2	45840250	45840446	chr1	-	TESK2	NM_007170	Intron
2	CD4 T	2	100187006	100187029	chr13	+	TM9SF2	NM_004800	Intron
2	MDM	2	25773022	25773120	chr1	-	TMEM57	NM_018202	Intron
2	CD4 T	2	91653011	91653124	chr8	+	TMEM64	NM_001146273	Intron
2	CD4 T	JR-FL	23012708	23012768	chr8	-	TNFRSF10D	NM_003840	Intron
2	MDM	2	145661797	145661847	chr8	-	TONSL	NM_013432	Undetermined
2	MDM	2	184617928	184618152	chr4	+	TRAPPC11	NM_199053	Intron
2	MDM	2	29490032	29490187	chr18	-	TRAPPC8	NM_014939	Intron
2	CD4 T	2	33973529	33973752	chr9	+	UBAP2	NM_001282529	Intron
2	CD4 T	2	4202769	4202907	chr17	+	UBE2G1	NM_003342	Intron
2	MDM	JR-FL	76156465	76156524	chr15	-	UBE2Q2	NM_001284382	Intron
2	CD4 T	2	48712178	48712219	chr20	-	UBE2V1	NM_001282578	Intron
2	MDM	JR-FL	10154332	10154356	chr1	-	UBE4B	NM_001105562	Intron
2	CD4 T	2	42568860	42568959	chr6	+	UBR2	NM_001184801	Intron
2	MDM	JR-FL	176343163	176343199	chr5	+	UIMC1	NM_016290	Intron
2	MDM	JR-FL	73801676	73801732	chr17	-	UNK	NM_001080419	Intron
2	MDM	JR-FL	62693428	62693486	chr12	-	USP15	NM_001252079	Intron
2	MDM	JR-FL	219376658	219376688	chr2	-	USP37	NM_020935	Intron
2	MDM	2	49358095	49358175	chr3	-	USP4	NM_199443	Intron
2	MDM	JR-FL	35069121	35069153	chr9	-	VCP	NM_007126	Intron
2	CD4 T	2	51463560	51463663	chr3	+	VPRBP	NM_001171904	Intron
2	MDM	1	713746	713845	chr16	-	WDR90	NM_145294	Intron
2	CD4 T	2	1941961	1942034	chr4	-	WHSC1	NM_001042424	Undetermined
2	MDM	JR-FL	41259083	41259282	chr22	+	XPNPEP3	NM_022098	Intron
2	CD4 T	2	28208848	28208950	chr16	-	XPO6	NM_001270940	Intron

2	CD4 T	2	42035005	42035071	chr22	+	XRCC6	NM_001469	Intron
2	MDM	2	87462862	87462916	chr16	-	ZCCHC14	NM_015144	Intron
2	MDM	JR-FL	9534616	9534680	chr11	+	ZNF143	NM_003442	Intron
2	MDM	2	44606316	44606548	chr19	+	ZNF224	NM_013398	Intron
2	CD4 T	2	21490531	21490596	chr19	-	ZNF708	NM_021269	Intron
2	MDM	2	55991613	55991646	chr7	-	ZNF713	NM_182633	Intron
2	CD4 T	JR-FL	9920830	9920868	chr19	-			Intergenic
2	CD4 T	JR-FL	26175309	26175470	chr6	-			Intergenic
2	MDM	1	49456785	49456940	chr12	+			Intergenic
2	MDM	1	97146679	97146773	chr2	-			Intergenic
2	MDM	1	28552377	28552404	chr16	-			Intergenic
2	MDM	1	197686993	197687185	chr2	+			Intergenic
2	MDM	2	105227722	105228019	chr14	-			Intergenic
2	MDM	2	34813376	34813529	chr3	+			Intergenic
2	MDM	2	201491516	201491676	chr1	+			Intergenic
2	MDM	2	6990308	6990349	chr17	-			Intergenic
2	MDM	2	7783408	7783504	chr17	+			Intergenic
2	MDM	2	2553337	2553470	chr4	-			Intergenic
2	MDM	2	35255662	35255853	chr2	+			Intergenic
2	MDM	2	61746954	61747103	chr11	-			Intergenic
2	MDM	2	68873897	68873973	chr12	+			Intergenic
2	MDM	2	1033687	1033718	chr20	-			Intergenic
2	MDM	2	45272905	45272980	chr19	+			Intergenic
2	MDM	2	105346994	105347200	chr12	+			Intergenic
2	MDM	2	20929384	20929571	chr13	+			Intergenic
2	MDM	2	39532917	39533135	chr7	+			Intergenic
2	MDM	2	95566878	95566928	chr5	+			Intergenic
2	MDM	JR-FL	73693145	73693173	chr7	+			Intergenic
2	MDM	JR-FL	47074067	47074093	chr21	-			Intergenic
2	MDM	JR-FL	7396328	7396353	chr12	+			Intergenic
2	MDM	JR-FL	65205737	65205813	chr11	-			Intergenic
2	MDM	JR-FL	4244718	4244900	chr19	-			Intergenic
2	CD4 T	2	25415288	25415347	chr22	-			Intergenic
2	CD4 T	2	7723129	7723219	chr19	-			Intergenic
2	CD4 T	2	47746863	47747007	chr12	+			Intergenic
2	CD4 T	2	144737487	144737596	chr8	+			Intergenic
2	CD4 T	2	50130477	50130589	chr19	-			Intergenic
2	CD4 T	2	4901917	4901957	chr3	+			Intergenic
2	CD4 T	2	21327287	21327310	chr21	-			Intergenic
3	CD4 T	3	114665573	114665832	chr2	+	ACTR3	NR_102318	Intron
3	MDM	3	367116	367155	chr16	+	AXIN1	NM_181050	Intron
3	CD4 T	3	179996882	179997066	chr5	+	CNOT6	NM_015455	Intron
3	CD4 T	3	38815743	38815918	chr21	-	DYRK1A	NM_101395	Intron
3	CD4 T	3	80052880	80053098	chr17	+	FASN	NM_004104	Intron
3	CD4 T	3	76593978	76594185	chr4	+	G3BP2	NM_012297	Intron
3	CD4 T	3	54875417	54875457	chr19	+	LAIR1	NR_110279	Intron
3	CD4 T	3	338241	338316	chr19	-	MIER2	NM_017550	Intron
3	CD4 T	3	40120367	40120524	chr4	+	N4BP2	NM_018177	Intron
3	CD4 T	3	43155384	43155541	chr17	+	NMT1	NM_021079	Intron
3	CD4 T	3	22983391	22983570	chr10	+	PIP4K2A	NM_005028	Intron
3	CD4 T	3	44126438	44126628	chr12	+	PUS7L	NM_001098614	Intron
3	CD4 T	3	141209998	141210229	chr3	+	RASA2	NM_006506	Intron
3	CD4 T	3	171947002	171947124	chr2	+	TLK1	NM_012290	Intron
3	CD4 T	3	75616251	75616297	chr16	-			Intergenic
3	CD4 T	3	30476308	30476404	chr16	-			Intergenic
3	CD4 T	3	75961840	75961933	chr17	-			Intergenic

3	CD4 T	3	6294273	6294293	chr20	+			Intergenic
3	CD4 T	3	142949734	142949808	chr7	-			Intergenic
4	MDM	4	202911244	202911371	chr1	+	ADIPOR1	NM_001290553	Intron
4	MDM	6	150822288	150822374	chr7	-	AGAP3	NM_001281300	Intron
4	MDM	4	50288653	50288808	chr19	-	AP2A1	NM_014203	Intron
4	MDM	4	72827991	72828058	chr15	-	ARIH1	NM_005744	Intron
4	MDM	6	123611741	123611770	chr10	+	ATE1	NM_001288734	Intron
4	CD4 T	4	66576186	66576228	chr11	+	C11orf80	NR_048553	Intron
4	MDM	6	3326902	3327009	chr20	+	C20orf194	NM_001009984	Intron
4	MDM	4	8934729	8934825	chr1	-	ENO1	NM_001428	Intron
4	CD4 T	4	5804867	5804947	chr9	+	ERMP1	NM_024896	Intron
4	MDM	4	915805	915836	chr4	+	GAK	NM_005255	Intron
4	MDM	4	73319311	73319483	chr17	-	GRB2	NM_002086	Intron
4	MDM	4	61348990	61349062	chr2	+	KIAA1841	NM_001129993	Intron
4	MDM	6	44850391	44850416	chr3	+	KIF15	NM_020242	Intron
4	MDM	4	1811679	1811815	chr16	+	MAPK8IP3	NM_001040439	Intron
4	MDM	4	786024	786072	chr16	-	NARFL	NM_022493	Intron
4	MDM	4	39926630	39926717	chr4	+	PDS5A	NM_001100399	Intron
4	MDM	4	139363692	139363785	chr9	-	SEC16A	NM_014866	Intron
4	CD4 T	4	46448689	46448854	chr17	+	SKAP1	NM_001075099	Intron
4	MDM	4	33752133	33752193	chr17	-	SLFN12	NM_018042	Intron
4	MDM	4	1637897	1637974	chr19	-	TCF3	NM_001136139	Intron
4	MDM	4	22076509	22076686	chr1	+	USP48	NM_032236	Intron
4	MDM	6	69757256	69757307	chr12	+	YEATS4	NM_006530	Intron
4	MDM	4	103837856	103837937	chr14	-			Intergenic
4	MDM	4	187381426	187381535	chr2	-			Intergenic
4	MDM	4	15404536	15404759	chr19	+			Intergenic
4	MDM	4	466908	466995	chr16	-			Intergenic
4	MDM	4	8228130	8228281	chr12	+			Intergenic
4	MDM	4	197825931	197826006	chr2	-			Intergenic
4	MDM	4	42726410	42726508	chr6	-			Intergenic
4	MDM	4	188908954	188908993	chr4	+			Intergenic
5	CD4 T	NL4-3	45311590	45311770	chr21	+	AGPAT3	NM_020132	Intron
5	CD4 T	NL4-3	42854810	42854927	chr13	-	AKAP11	NM_016248	Intron
5	MDM	6	69314480	69314537	chr7	+	AUTS2	NM_001127232	Intron
5	CD4 T	NL4-3	60816586	60816696	chr18	+	BCL2	NM_000633	Intron
5	CD4 T	NL4-3	41214027	41214094	chr17	-	BRCA1	NM_007298	Intron
5	MDM	6	32905424	32905482	chr13	-	BRCA2	NM_000059	Intron
5	CD4 T	NL4-3	48751383	48751524	chr19	+	CARD8	NR_033678	Intron
5	CD4 T	NL4-3	96019509	96019687	chr5	+	CAST	NM_001190442	Intron
5	CD4 T	NL4-3	105484213	105484273	chr14	-	CDCA4	NM_017955	Intron
5	CD4 T	NL4-3	56334956	56335044	chr4	-	CLOCK	NM_001267843	Intron
5	CD4 T	NL4-3	58569093	58569303	chr16	+	CNOT1	NR_049763	Intron
5	CD4 T	NL4-3	14041291	14041454	chr17	-	COX10	NM_001303	Intron
5	CD4 T	NL4-3	1249371	1249431	chr1	+	CPSF3L	NM_017871	Intron
5	MDM	6	76716985	76717071	chr17	+	CYTH1	NM_001292018	Intron
5	CD4 T	NL4-3	68958470	68958576	chr1	+	DEPDC1	NM_017779	Intron
5	MDM	6	127656740	127656941	chr6	-	ECHDC1	NM_001139510	Intron
5	CD4 T	5	15885736	15885830	chr10	-	FAM188A	NM_024948	Intron
5	MDM	6	697635	697715	chr16	+	FAM195A	NM_138418	Intron
5	CD4 T	NL4-3	76209518	76209650	chr15	-	FBXO22	NM_012170	Undetermined
5	MDM	6	151054448	151054552	chr1	-	GABPB2	NM_144618	Intron
5	CD4 T	NL4-3	19567214	19567404	chr19	+	GATAD2A	NM_017660	Intron
5	MDM	6	19529705	19529855	chr16	+	GDE1	NM_016641	Intron
5	CD4 T	NL4-3	62235777	62235822	chr20	+	GMEB2	NM_012384	Intron
5	CD4 T	NL4-3	28816644	28816683	chr17	-	GOSR1	NM_004871	Intron

5	CD4 T	NL4-3	46344606	46344809	chr21	+	ITGB2	NM_001127491	Intron
5	CD4 T	NL4-3	6096303	6096376	chr4	-	JAKMIP1	NM_001099433	Intron
5	MDM	6	17958492	17958563	chr6	+	KIF13A	NM_001105568	Intron
5	CD4 T	NL4-3	47190615	47190645	chr13	-	LRCH1	NM_001164211	Intron
5	MDM	6	139985287	139985322	chr9	+	MAN1B1	NM_016219	Intron
5	CD4 T	NL4-3	43374344	43374562	chr17	-	MAP3K14	NM_003954	Intron
5	CD4 T	NL4-3	123713287	123713451	chr12	-	MPHOSPH9	NM_022782	Intron
5	CD4 T	NL4-3	16036716	16036915	chr17	+	NCOR1	NM_001190440	Intron
5	CD4 T	NL4-3	2941261	2941291	chr4	-	NOP14-AS1	NR_015453	Undetermined
5	MDM	6	33607497	33607572	chr10	+	NRP1	NM_003873	Intron
5	CD4 T	NL4-3	4591958	4592013	chr17	+	PELP1	NM_014389	Intron
5	MDM	6	71886074	71886198	chrX	-	PHKA1	NM_002637	Exon
5	CD4 T	NL4-3	65038570	65038638	chr11	+	POLA2	NM_002689	Intron
5	CD4 T	NL4-3	26167993	26168165	chr8	-	PPP2R2A	NM_002717	Intron
5	MDM	6	57127715	57127923	chr12	+	PRIM1	NM_000946	Intron
5	CD4 T	NL4-3	48870150	48870248	chr3	+	PRKAR2A	NM_004157	Intron
5	CD4 T	NL4-3	48726761	48726918	chr8	+	PRKDC	NM_006904	Intron
5	MDM	6	48059839	48059876	chr21	+	PRMT2	NM_001242865	Intron
5	MDM	6	6049306	6049359	chr19	-	RFX2	NM_000635	Intron
5	CD4 T	NL4-3	74209980	74210054	chr17	+	RNF157	NM_052916	Intron
5	CD4 T	NL4-3	127892611	127892846	chr9	-	SCAI	NM_173690	Intron
5	CD4 T	NL4-3	150323413	150323588	chr3	-	SELT	NM_016275	Intron
5	MDM	6	83800348	83800445	chr7	-	SEMA3A	NM_006080	Intron
5	CD4 T	NL4-3	52087956	52087984	chr10	-	SGMS1	NM_147156	Intron
5	MDM	6	2145503	2145579	chr9	+	SMARCA2	NM_139045	Intron
5	MDM	6	2796226	2796451	chr19	+	THOP1	NM_003249	Intron
5	CD4 T	NL4-3	36691333	36691484	chr1	+	THRAP3	NM_005119	Intron
5	CD4 T	NL4-3	76059676	76059857	chr17	+	TNRC6C	NM_018996	Intron
5	CD4 T	NL4-3	139795212	139795397	chr9	+	TRAF2	NM_021138	Intron
5	MDM	6	45466926	45466999	chr21	+	TRAPPC10	NM_003274	Intron
5	MDM	6	2132992	2133143	chr16	-	TSC2	NM_001077183	Intron
5	CD4 T	NL4-3	4225357	4225427	chr17	-	UBE2G1	NM_003342	Intron
5	CD4 T	NL4-3	157002259	157002437	chr7	-	UBE3C	NM_014671	Intron
5	MDM	6	12478609	12478712	chr1	+	VPS13D	NM_015378	Intron
5	CD4 T	NL4-3	33253719	33253830	chr1	-	YARS	NM_003680	Intron
5	CD4 T	NL4-3	338596	338625	chr4	+	ZNF141	NM_003441	Intron
5	CD4 T	NL4-3	52407677	52407878	chr20	+			Intergenic
5	CD4 T	NL4-3	149853808	149854016	chr1	+			Intergenic
5	CD4 T	NL4-3	49996380	49996602	chr19	-			Intergenic
5	CD4 T	NL4-3	50334701	50334883	chr22	-			Intergenic
5	CD4 T	NL4-3	146290536	146290747	chr8	+			Intergenic
5	CD4 T	NL4-3	3664497	3664726	chr10	-			Intergenic
5	CD4 T	NL4-3	112065944	112066115	chr10	+			Intergenic
5	CD4 T	NL4-3	3129592	3129701	chr16	-			Intergenic
5	MDM	6	60127572	60127718	chr11	+			Intergenic
5	MDM	6	89307513	89307550	chr13	+			Intergenic
5	MDM	6	32347784	32347830	chr6	+			Intergenic
5	MDM	6	35843220	35843338	chr17	+			Intergenic
5	MDM	6	208142794	208142848	chr2	+			Intergenic
5	CD4 T	NL4-3	22475274	22475352	chr15	+			Intergenic
5	CD4 T	NL4-3	106384882	106384976	chr6	+			Intergenic
6	MDM	6	35377991	35378157	chr17	+	AATF	NM_012138	Intron
6	MDM	6	2444743	2444807	chr16	-	ABCA17P	NR_003574	Intron
6	MDM	6	935991	936085	chr17	-	ABR	NM_001282149	Intron
6	MDM	JR-FL	1080287	1080414	chr17	+	ABR	NM_021962	Intron
6	CD4 T	JR-FL	39161737	39161854	chr19	+	ACTN4	NM_004924	Intron

6	MDM	6	114699773	114699948	chr2	-	ACTR3	NR_102318	Undetermined
6	MDM	6	43637094	43637186	chr15	+	ADAL	NM_001012969	Intron
6	CD4 T	6	49544324	49544477	chr20	+	ADNP	NM_001282531	Intron
6	CD4 T	6	244605430	244605580	chr1	-	ADSS	NM_001126	Intron
6	MDM	6	247028711	247028753	chr1	-	AHCTF1	NM_015446	Intron
6	MDM	6	49970312	49970435	chr19	+	ALDH16A1	NM_001145396	Intron
6	MDM	6	111688284	111688305	chr11	+	ALG9	NM_001077691	Intron
6	MDM	6	46516799	46516912	chr11	+	AMBRA1	NM_017749	Intron
6	MDM	6	46547920	46548059	chr11	+	AMBRA1	NM_017749	Intron
6	MDM	JR-FL	139849112	139849138	chr5	+	ANKHD1	NM_017978	Intron
6	MDM	6	43646219	43646247	chr3	-	ANO10	NM_001204831	Intron
6	MDM	6	22226228	22226337	chr11	+	ANO5	NM_001142649	Intron
6	MDM	6	954804	954914	chr11	+	AP2A2	NM_001242837	Intron
6	MDM	6	33998791	33998909	chr17	+	AP2B1	NM_001030006	Exon
6	CD4 T	6	33949210	33949249	chr17	-	AP2B1	NM_001030006	Intron
6	CD4 T	6	33953381	33953443	chr17	+	AP2B1	NM_001030006	Intron
6	MDM	6	114422541	114422650	chr1	-	AP4B1-AS1	NR_037864	Undetermined
6	CD4 T	6	39443483	39443522	chr22	+	APOBEC3F	NM_145298	Intron
6	CD4 T	6	35225833	35225874	chr15	+	AQR	NM_014691	Intron
6	MDM	6	36118958	36119081	chr4	+	ARAP2	NM_015230	Intron
6	MDM	6	148947965	148947987	chr4	-	ARHGAP10	NM_024605	Intron
6	MDM	6	47424985	47425041	chr19	+	ARHGAP35	NM_004491	Exon
6	CD4 T	6	155941379	155941417	chr1	+	ARHGEF2	NM_004723	Intron
6	MDM	6	74843948	74844019	chr15	-	ARID3B	NM_006465	Intron
6	CD4 T	6	48965494	48965590	chr3	-	ARIH2	NM_006321	Intron
6	CD4 T	6	49004797	49004945	chr3	+	ARIH2	NM_006321	Intron
6	MDM	6	100882665	100882779	chrX	-	ARMCX3	NM_016607	3´UTR
6	MDM	6	66290022	66290211	chr17	+	ARSG	NM_014960	Intron
6	MDM	6	14540287	14540314	chr12	+	ATF7IP	NM_001286515	Intron
6	MDM	6	11343951	11344095	chr3	+	ATG7	NM_001136031	Intron
6	MDM	6	108157412	108157465	chr11	-	ATM	NM_000051	Intron
6	MDM	6	182578019	182578229	chr3	-	ATP11B	NM_014616	Intron
6	MDM	6	142221820	142221845	chr3	+	ATR	NM_001184	Intron
6	CD4 T	6	111908473	111908573	chr12	+	ATXN2	NM_002973	Intron
6	MDM	JR-FL	30851865	30851904	chr16	-	BCL7C	NM_001286526	Intron
6	CD4 T	6	70758318	70758429	chrX	+	BCYRN1	NR_001568	Intron
6	CD4 T	6	70770663	70770751	chrX	-	BCYRN1	NR_001568	Intron
6	MDM	6	28617116	28617176	chr17	+	BLMH	NM_000386	Intron
6	CD4 T	6	145512273	145512305	chr8	+	BOP1	NM_015201	Intron
6	MDM	6	73311282	73311422	chr13	+	BORA	NM_001286747	Intron
6	MDM	JR-FL	41219671	41219706	chr17	+	BRCA1	NM_007298	Exon
6	MDM	6	40623441	40623571	chr21	+	BRWD1	NM_018963	Intron
6	CD4 T	6	103304621	103304657	chr10	-	BTRC	NM_001256856	Intron
6	MDM	6	103713579	103713601	chr10	+	C10orf76	NM_024541	Intron
6	MDM	JR-FL	66598748	66598795	chr11	+	C11orf80	NM_024650	Intron
6	MDM	6	36985196	36985245	chr15	+	C15orf41	NM_001290233	Intron
6	MDM	JR-FL	41861128	41861162	chr17	-	C17orf105	NM_001136483	Intron
6	MDM	JR-FL	3347549	3347583	chr20	-	C20orf194	NM_001009984	Intron
6	MDM	JR-FL	43344150	43344179	chr21	+	C2CD2	NM_199050	Intron
6	MDM	6	73820284	73820418	chr11	+	C2CD3	NM_001286577	Intron
6	MDM	JR-FL	47349944	47350006	chr2	-	C2orf61	NM_001163561	Intron
6	MDM	6	32293579	32293699	chr6	-	C6orf10	NM_006781	Intron
6	MDM	6	34617818	34617910	chr6	-	C6orf106	NM_024294	Intron
6	MDM	JR-FL	71280990	71281028	chr6	-	C6orf57	NM_145267	Intron
6	MDM	JR-FL	87965426	87965502	chr16	-	CA5A	NM_001739	Intron
6	MDM	6	115290044	115290164	chr11	+	CADM1	NM_014333	Intron

6	MDM	JR-FL	15281702	15281726	chr3	+	CAPN7	NM_014296	Intron
6	MDM	6	105448909	105448984	chr3	-	CBLB	NM_170662	Intron
6	MDM	JR-FL	131633122	131633185	chr9	+	CCBL1	NR_109829	Intron
6	MDM	6	49914491	49914528	chr19	+	CCDC155	NM_144688	Intron
6	MDM	6	43103690	43103820	chr1	+	CCDC30	NM_001080850	Intron
6	MDM	6	80156278	80156359	chr17	-	CCDC57	NM_198082	Exon
6	MDM	6	80149376	80149412	chr17	+	CCDC57	NM_198082	Intron
6	MDM	6	80082290	80082355	chr17	+	CCDC57	NM_198082	Intron
6	MDM	6	34316568	34316616	chr17	-	CCL15-CCL14	NR_027922	Intron
6	CD4 T	6	45053566	45053610	chr7	-	CCM2	NR_030770	Intron
6	MDM	6	156285844	156285894	chr1	-	CCT3	NR_036565	Intron
6	MDM	6	5461926	5462042	chr9	+	CD274	NR_052005	Intron
6	CD4 T	6	111296252	111296368	chr3	+	CD96	NM_198196	Undetermined
6	MDM	6	2599889	2599933	chrY	+	CD99	NM_002414	Intron
6	MDM	6	103500310	103500381	chr14	-	CDC42BPB	NM_006035	Intron
6	MDM	6	96744462	96744615	chr12	+	CDK17	NM_001170464	Intron
6	MDM	6	92380671	92380756	chr7	-	CDK6	NM_001259	Intron
6	MDM	6	80966648	80966743	chr14	+	CEP128	NM_152446	Intron
6	CD4 T	6	40162218	40162348	chr20	+	CHD6	NM_032221	Intron
6	CD4 T	6	66603630	66603831	chr16	-	CKLF-CMTM1	NM_001202509	Intron
6	MDM	JR-FL	1512941	1512977	chr16	+	CLCN7	NM_001114331	Intron
6	MDM	6	9850936	9851022	chr12	+	CLEC2D	NM_001004419	3´UTR
6	CD4 T	6	9831835	9832006	chr12	+	CLEC2D	NM_001197318	Intron
6	MDM	6	122828233	122828472	chr12	-	CLIP1	NM_001247997	Intron
6	MDM	JR-FL	29368176	29368238	chr2	+	CLIP4	NM_001287528	Intron
6	MDM	6	72080887	72080963	chr11	+	CLPB	NM_001258393	Intron
6	MDM	6	135121238	135121259	chr7	+	CNOT4	NM_013316	Intron
6	MDM	JR-FL	7846933	7846959	chr17	+	CNTROB	NM_001037144	Intron
6	CD4 T	6	107193129	107193166	chr7	-	COG5	NM_181733	Intron
6	MDM	JR-FL	160296538	160296578	chr1	-	COPA	NM_001098398	Intron
6	MDM	6	160287917	160288019	chr1	+	COPA	NM_001098398	Undetermined
6	MDM	6	109075119	109075157	chr12	+	CORO1C	NM_001105237	Intron
6	MDM	6	28793201	28793316	chr17	-	CPD	NM_001304	3´UTR
6	MDM	6	93967101	93967206	chr10	-	CPEB3	NM_001178137	Intron
6	MDM	6	39101853	39101873	chr12	+	CPNE8	NM_153634	Intron
6	CD4 T	6	211343274	211343533	chr2	-	CPS1	NM_001122633	Intron
6	MDM	6	1689075	1689147	chr16	+	CRAMP1L	NM_020825	Intron
6	MDM	6	1328009	1328056	chr17	+	CRK	NM_005206	Intron
6	MDM	6	18146437	18146553	chr20	-	CSRP2BP	NM_020536	Intron
6	CD4 T	6	8131334	8131399	chr17	+	CTC1	NM_025099	3´UTR
6	MDM	1	7151968	7152051	chr17	+	CTDNEP1	NM_001143775	Intron
6	MDM	6	150714853	150715168	chr1	-	CTSS	NM_001199739	Intron
6	MDM	JR-FL	49547379	49547421	chr3	+	DAG1	NM_001177643	Intron
6	MDM	6	61480095	61480185	chr11	+	DAGLA	NM_006133	Intron
6	CD4 T	6	6481491	6481575	chr7	+	DAGLB	NM_001142936	Intron
6	MDM	6	668469	668556	chr11	-	DEAF1	NM_021008	Intron
6	MDM	6	65981074	65981276	chr15	+	DENND4A	NM_005848	Intron
6	CD4 T	6	47946240	47946308	chr21	-	DIP2A	NM_206891	Intron
6	CD4 T	6	122531712	122531786	chr3	-	DIRC2	NM_032839	Intron
6	CD4 T	6	102963327	102963373	chr7	-	DNAJC2	NM_014377	Intron
6	CD4 T	6	102973921	102974003	chr7	-	DNAJC2	NM_001129887	Intron
6	MDM	6	10900040	10900118	chr19	+	DNM2	NM_004945	Intron
6	MDM	6	225691089	225691221	chr2	+	DOCK10	NM_001290263	Intron
6	MDM	6	116702495	116702581	chr6	-	DSE	NM_001080976	Intron
6	MDM	6	15605981	15606125	chr6	-	DTNBP1	NM_032122	Intron
6	MDM	6	46799714	46799893	chr18	+	DYM	NM_017653	Intron

6	MDM	6	127425529	127425692	chr10	+	EDRF1	NR_110857	Intron
6	CD4 T	6	85987292	85987364	chr11	-	EED	NM_152991	Intron
6	MDM	6	25276078	25276186	chr2	+	EFR3B	NM_014971	Intron
6	MDM	JR-FL	6096263	6096339	chr7	+	EIF2AK1	NM_014413	Intron
6	CD4 T	6	40257099	40257257	chr15	+	EIF2AK4	NM_001013703	Intron
6	CD4 T	6	120809279	120809329	chr10	+	EIF3A	NM_003750	Exon
6	CD4 T	6	53418794	53418820	chr12	-	EIF4B	NM_001417	Intron
6	CD4 T	6	99803627	99803757	chr4	+	EIF4E	NM_001130678	Intron
6	MDM	6	8065663	8065715	chr19	+	ELAVL1	NM_001419	Intron
6	MDM	6	130615715	130615749	chr9	+	ENG	NM_001114753	Intron
6	MDM	6	101449004	101449134	chr10	+	ENTPD7	NM_020354	Intron
6	CD4 T	6	29339073	29339169	chr1	-	EPB41	NM_001166007	Intron
6	MDM	6	131268652	131268736	chr6	+	EPB41L2	NM_001252660	Intron
6	MDM	6	62146796	62146859	chr17	+	ERN1	NM_001433	Intron
6	CD4 T	6	76580679	76580761	chr15	-	ETFA	NM_001127716	Intron
6	MDM	6	94788232	94788289	chr10	+	EXOC6	NM_001013848	Intron
6	MDM	6	143594814	143594925	chr7	-	FAM115A	NM_014719	Intron
6	MDM	6	130314346	130314416	chr9	+	FAM129B	NM_022833	Intron
6	MDM	6	40739686	40739780	chr17	+	FAM134C	NM_178126	Intron
6	MDM	6	73243596	73243622	chr11	+	FAM168A	NM_001286050	Intron
6	MDM	6	73147657	73147776	chr11	+	FAM168A	NM_001286051	Intron
6	MDM	6	155223417	155223458	chr1	-	FAM189B	NM_001267608	Exon
6	MDM	6	159090021	159090164	chr4	-	FAM198B	NM_016613	Intron
6	CD4 T	6	89854866	89854913	chr16	+	FANCA	NM_000135	Intron
6	CD4 T	6	89831044	89831115	chr16	-	FANCA	NM_000135	Intron
6	MDM	6	73925491	73925529	chr17	+	FBF1	NM_001080542	Intron
6	MDM	6	72680784	72680860	chr11	-	FCHSD2	NM_014824	Intron
6	MDM	6	72814239	72814292	chr11	+	FCHSD2	NM_014824	Intron
6	CD4 T	6	95724084	95724195	chr9	-	FGD3	NM_001083536	Intron
6	MDM	6	241670155	241670233	chr1	+	FH	NM_000143	Intron
6	MDM	6	81185611	81185710	chr17	-	FLJ43681	NR_029406	Intron
6	MDM	6	76069718	76069868	chr14	+	FLVCR2	NM_017791	Intron
6	CD4 T	6	147001963	147002177	chrX	+	FMR1-AS1	NR_024502	Intron
6	CD4 T	6	132760877	132760940	chr9	+	FNBP1	NM_015033	Intron
6	CD4 T	6	48557061	48557117	chr2	-	FOXN2	NM_002158	Intron
6	CD4 T	6	180688643	180688744	chr3	+	FXR1	NM_001013439	Intron
6	MDM	JR-FL	7494684	7494730	chr17	+	FXR2	NM_004860	3´UTR
6	MDM	6	854264	854308	chr4	+	GAK	NM_005255	Intron
6	MDM	6	153860712	153860858	chr1	+	GATAD2B	NM_020699	Intron
6	MDM	6	153827526	153827585	chr1	+	GATAD2B	NM_020699	Intron
6	MDM	6	155195249	155195283	chr1	-	GBAP1	NR_002188	Intron
6	MDM	6	81639096	81639215	chr3	-	GBE1	NM_000158	Intron
6	CD4 T	6	89647393	89647460	chr1	+	GBP4	NM_052941	3´UTR
6	MDM	6	158379118	158379250	chr3	-	GFM1	NM_024996	Intron
6	MDM	6	110093498	110093571	chr1	-	GNAI3	NM_006496	Intron
6	MDM	6	2532297	2532374	chr19	-	GNG7	NM_052847	Intron
6	CD4 T	6	150658857	150658950	chr1	+	GOLPH3L	NM_018178	Intron
6	MDM	6	155810978	155811045	chr1	+	GON4L	NM_001282858	Intron
6	MDM	6	157327262	157327342	chr2	-	GPD2	NM_001083112	Intron
6	MDM	6	89939580	89939702	chr5	-	GPR98	NM_032119	Intron
6	MDM	6	1874766	1874862	chr16	+	HAGH	NM_005326	Intron
6	CD4 T	6	48665020	48665152	chrX	-	HDAC6	NM_006044	Exon
6	MDM	6	242223739	242223965	chr2	-	HDLBP	NM_005336	Intron
6	MDM	6	79656258	79656384	chr17	+	HGS	NM_004712	Intron
6	MDM	JR-FL	149767367	149767392	chr1	+	HIST2H2BF	NM_001161334	Intron
6	CD4 T	6	73140705	73140727	chr17	+	HN1	NM_001288610	Intron

6	MDM	6	1731907	1732004	chr16	-	HN1L	NM_144570	Intron
6	CD4 T	6	179041467	179041588	chr5	+	HNRNPH1	NM_001257293	3´UTR
6	CD4 T	6	30557156	30557201	chr22	-	HORMAD2	NM_152510	Intron
6	CD4 T	6	30563468	30563585	chr22	+	HORMAD2	NM_152510	Intron
6	CD4 T	6	30529772	30529848	chr22	+	HORMAD2	NM_152510	Intron
6	MDM	6	115166362	115166539	chr9	+	HSDL2	NR_036651	Intron
6	MDM	6	145523777	145523832	chr8	-	HSF1	NM_005526	Intron
6	MDM	6	44960684	44960801	chr21	+	HSF2BP	NM_007031	Intron
6	MDM	JR-FL	102548121	102548149	chr14	+	HSP90AA1	NM_005348	Exon
6	MDM	JR-FL	55779288	55779322	chr19	+	HSPBP1	NM_001130106	Intron
6	CD4 T	6	82915912	82916002	chr6	-	IBTK	NM_015525	Intron
6	CD4 T	6	37971685	37971807	chr17	+	IKZF3	NM_001257409	Intron
6	MDM	6	50430925	50431030	chr19	-	IL4I1	NR_047577	Intron
6	CD4 T	6	41340412	41340527	chr15	+	INO80	NM_017553	Intron
6	MDM	6	99065462	99065580	chr2	+	INPP4A	NM_001566	Intron
6	MDM	6	143460316	143460382	chr4	-	INPP4B	NM_001101669	Intron
6	MDM	6	49816957	49817026	chr3	-	IP6K1	NM_001242829	Intron
6	CD4 T	6	2604964	2605115	chr7	-	IQCE	NM_001287500	Intron
6	CD4 T	6	64984234	64984325	chr10	-	JMJD1C	NM_001282948	Intron
6	MDM	6	79158160	79158323	chr10	-	KCNMA1	NM_001014797	Intron
6	MDM	JR-FL	66940650	66940706	chr11	+	KDM2A	NM_012308	Intron
6	CD4 T	6	66988684	66988708	chr11	+	KDM2A	NM_012308	Intron
6	MDM	JR-FL	6828038	6828058	chr4	-	KIAA0232	NM_014743	Intron
6	CD4 T	6	6801342	6801479	chr4	+	KIAA0232	NM_001100590	Intron
6	MDM	6	123272206	123272362	chr4	-	KIAA1109	NM_015312	Intron
6	MDM	6	24153236	24153274	chr10	-	KIAA1217	NM_001098500	Intron
6	CD4 T	6	25468043	25468114	chr22	-	KIAA1671	NM_001145206	Intron
6	CD4 T	6	10294137	10294201	chr1	-	KIF1B	NM_183416	Intron
6	MDM	6	39811019	39811091	chr12	-	KIF21A	NM_001173463	Intron
6	MDM	6	45421439	45421538	chr14	-	KLHL28	NM_017658	Intron
6	MDM	6	151995319	151995343	chr7	+	KMT2C	NM_170606	Intron
6	MDM	6	45735609	45735696	chr17	+	KPNB1	NM_002265	Intron
6	MDM	6	36109030	36109137	chr11	-	LDLRAD3	NM_174902	Intron
6	MDM	6	62932714	62932803	chr20	-	LINC00266-1	NR_040415	Intron
6	MDM	6	177164883	177165040	chr3	+	LINC00578	NR_047568	Intron
6	MDM	6	36933287	36933342	chr18	+	LINC00669	NR_024391	Intron
6	MDM	6	33894683	33894729	chr11	+	LMO2	NM_005574	Intron
6	MDM	JR-FL	2503854	2503903	chr1	-	LOC100996583	NR_121638	Intron
6	MDM	6	98017267	98017293	chr14	-	LOC101929241	NR_110166	Intron
6	CD4 T	6	54039202	54039270	chr5	+	LOC102467080	NR_104658	Intron
6	MDM	6	243057439	243057477	chr2	-	LOC728323	NR_024437	Intron
6	CD4 T	6	34681039	34681061	chr19	-	LSM14A	NM_015578	Intron
6	CD4 T	6	31770511	31770702	chr6	-	LSM2	NM_021177	Intron
6	CD4 T	6	147103418	147103481	chr4	-	LSM6	NM_007080	Intron
6	MDM	6	33260089	33260144	chr2	+	LTBP1	NM_206943	Intron
6	MDM	JR-FL	6495242	6495342	chr12	+	LTBR	NM_001270987	Intron
6	MDM	6	264695	264719	chr16	+	LUC7L	NM_018032	Intron
6	CD4 T	6	139059218	139059278	chr7	-	LUC7L2	NM_001244585	Intron
6	MDM	JR-FL	31680067	31680089	chr6	-	LY6G6E	NR_024541	Intron
6	MDM	6	235887592	235887771	chr1	-	LYST	NM_000081	Intron
6	MDM	6	39888424	39888590	chr1	+	MACF1	NM_012090	Intron
6	MDM	6	139988842	139988883	chr9	-	MAN1B1	NM_016219	Intron
6	MDM	6	12761602	12761634	chr19	+	MAN2B1	NM_000528	Intron
6	MDM	6	67927091	67927271	chr15	-	MAP2K5	NM_145160	Intron
6	MDM	JR-FL	53892464	53892539	chr12	-	MAP3K12	NM_001193511	Intron
6	MDM	6	137078373	137078530	chr6	-	MAP3K5	NM_005923	Intron

6	CD4 T	6	39090909	39091056	chr19	+	MAP4K1	NM_007181	Intron
6	MDM	6	46312518	46312564	chr1	+	MAST2	NM_015112	Intron
6	MDM	6	148818162	148818344	chr2	-	MBD5	NM_018328	Intron
6	MDM	JR-FL	94474793	94474839	chr5	+	MCTP1	NM_024717	Intron
6	MDM	6	153327820	153327973	chrX	+	MECP2	NM_004992	Intron
6	MDM	6	153339314	153339361	chrX	+	MECP2	NM_004992	Intron
6	MDM	JR-FL	153303340	153303382	chrX	+	MECP2	NM_004992	Intron
6	CD4 T	6	153334197	153334269	chrX	+	MECP2	NM_004992	Intron
6	MDM	6	134837331	134837392	chr9	-	MED27	NM_004269	Intron
6	CD4 T	6	6549423	6549535	chr17	-	MED31	NM_016060	Intron
6	MDM	6	112667199	112667272	chr2	-	MERTK	NM_006343	Intron
6	MDM	6	112680304	112680435	chr2	+	MERTK	NM_006343	Intron
6	MDM	6	39334279	39334375	chr4	+	MIR1273H	NR_106996	Intron
6	CD4 T	6	99591572	99591621	chr3	+	MIR548G	NR_031662	Intron
6	MDM	6	156088192	156088250	chr1	-	MIR7851	NR_107005	Intron
6	MDM	6	146576706	146576765	chr4	-	MMAA	NM_172250	3´UTR
6	MDM	6	108745228	108745317	chr3	+	MORC1	NM_014429	Intron
6	MDM	6	94203468	94203611	chr11	+	MRE11A	NM_005590	Intron
6	MDM	6	145218750	145218903	chr8	-	MROH1	NM_001099281	Exon
6	MDM	1	145232001	145232052	chr8	-	MROH1	NM_001099281	Intron
6	MDM	6	125055716	125055851	chr9	-	MRRF	NM_138777	Intron
6	MDM	6	60073888	60073909	chr11	+	MS4A4A	NM_024021	Intron
6	MDM	JR-FL	60070651	60070732	chr11	-	MS4A4A	NM_148975	Intron
6	MDM	6	31727021	31727067	chr6	+	MSH5	NM_002441	Intron
6	CD4 T	6	55338741	55338812	chr17	-	MSI2	NM_138962	Intron
6	MDM	JR-FL	166259214	166259261	chr4	-	MSMO1	NM_006745	Intron
6	MDM	6	30312705	30312863	chr22	-	MTMR3	NM_153050	Intron
6	CD4 T	6	56547134	56547190	chr12	+	MYL6B	NM_001199629	Intron
6	CD4 T	6	164084885	164084948	chr4	-	NAF1	NM_001128931	Intron
6	MDM	6	201630623	201630718	chr1	+	NAV1	NM_020443	Intron
6	MDM	6	136654179	136654246	chr3	+	NCK1	NM_006153	Intron
6	MDM	6	24816886	24816929	chr2	-	NCOA1	NM_003743	Intron
6	CD4 T	6	46224340	46224458	chr20	-	NCOA3	NM_181659	Intron
6	CD4 T	6	29657782	29657893	chr17	+	NF1	NM_000267	Intron
6	CD4 T	6	68153154	68153292	chr16	-	NFATC3	NM_173165	Intron
6	CD4 T	6	68177237	68177285	chr16	+	NFATC3	NM_173165	Intron
6	MDM	6	41202911	41203004	chr1	-	NFYC	NM_001142587	Intron
6	CD4 T	6	36997481	36997696	chr5	-	NIPBL	NM_015384	Intron
6	MDM	6	50068544	50068600	chr19	-	NOSIP	NM_015953	Intron
6	MDM	6	50065359	50065385	chr19	-	NOSIP	NM_015953	Intron
6	MDM	6	73910326	73910454	chr14	+	NUMB	NM_001005743	Intron
6	MDM	6	134065770	134065856	chr9	-	NUP214	NM_005085	Intron
6	CD4 T	6	44671793	44671864	chr7	-	OGDH	NM_001003941	Intron
6	MDM	6	107659872	107659914	chr8	-	OXR1	NM_018002	Intron
6	MDM	6	74771706	74771812	chr10	+	P4HA1	NM_000917	Intron
6	MDM	6	74771871	74771939	chr10	-	P4HA1	NM_001142596	Intron
6	MDM	6	49029277	49029329	chr3	+	P4HTM	NM_177938	Intron
6	MDM	6	66005724	66005750	chr11	+	PACS1	NM_018026	Intron
6	MDM	JR-FL	2447368	2447416	chr1	-	PANK4	NM_018216	Intron
6	MDM	JR-FL	72536870	72536920	chr15	+	PARP6	NM_020214	Intron
6	MDM	6	1487511	1487538	chr19	-	PCSK4	NM_017573	Intron
6	MDM	6	17629661	17629860	chr19	-	PGLS	NM_012088	Intron
6	MDM	6	129194015	129194182	chr4	+	PGRMC2	NM_006320	Intron
6	CD4 T	6	79780924	79781024	chr6	-	PHIP	NM_017934	Intron
6	MDM	6	39653781	39653885	chr18	-	PIK3C3	NM_002647	Intron
6	CD4 T	6	9719286	9719463	chr1	-	PIK3CD	NM_005026	Intron

6	CD4 T	6	67554053	67554079	chr5	+	PIK3R1	NM_181523	Intron
6	CD4 T	6	65180906	65181020	chr14	-	PLEKHG3	NM_015549	Intron
6	MDM	1	50732968	50733095	chr22	-	PLXNB2	NM_012401	Intron
6	MDM	6	139307642	139307710	chr9	-	PMPCA	NM_001282946	Intron
6	MDM	6	65053341	65053389	chr11	+	POLA2	NM_002689	Intron
6	MDM	6	106817496	106817628	chr12	+	POLR3B	NM_018082	Intron
6	CD4 T	6	167299624	167299676	chr1	-	POU2F1	NM_001198786	Intron
6	MDM	6	51582055	51582134	chr12	+	POU6F1	NM_002702	3´UTR
6	MDM	6	55618201	55618341	chr19	-	PPP1R12C	NM_017607	Intron
6	MDM	JR-FL	68259443	68259484	chr11	+	PPP6R3	NM_001164161	Intron
6	MDM	JR-FL	57125987	57126063	chr12	-	PRIM1	NM_000946	Intron
6	CD4 T	6	48827893	48827943	chr3	-	PRKAR2A	NM_004157	Intron
6	CD4 T	6	23950942	23951069	chr16	+	PRKCB	NM_212535	Intron
6	MDM	JR-FL	68371324	68371395	chr16	-	PRMT7	NM_001184824	Intron
6	MDM	6	150307767	150307928	chr1	-	PRPF3	NM_004698	Intron
6	MDM	6	231994216	231994335	chr2	-	PSMD1	NM_001191037	Intron
6	MDM	6	232013319	232013482	chr2	+	PSMD1	NM_002807	Intron
6	MDM	6	40990203	40990227	chr17	+	PSME3	NM_005789	Intron
6	MDM	6	1113742	1113865	chr20	-	PSMF1	NM_178578	Intron
6	MDM	6	809703	809783	chr19	-	PTBP1	NM_031990	Intron
6	MDM	6	454197	454363	chr11	+	PTDSS2	NM_030783	Intron
6	CD4 T	6	198705252	198705309	chr1	+	PTPRC	NM_080921	Intron
6	CD4 T	6	58350859	58350966	chr3	-	PXK	NM_017771	Intron
6	MDM	JR-FL	49093688	49093838	chr3	+	QRICH1	NM_198880	Intron
6	MDM	6	99998292	99998328	chr10	+	R3HCC1L	NM_001256620	Intron
6	MDM	6	135844852	135844932	chr2	-	RAB3GAP1	NM_012233	Intron
6	MDM	JR-FL	653335	653462	chr16	-	RAB40C	NM_001172663	Intron
6	CD4 T	6	5197692	5197736	chr17	+	RABEP1	NM_001291582	Intron
6	CD4 T	6	5239009	5239074	chr17	-	RABEP1	NM_001291582	Intron
6	MDM	JR-FL	139709758	139709783	chr9	-	RABL6	NM_001173989	Intron
6	CD4 T	6	39691010	39691141	chr7	+	RALA	NM_005402	Intron
6	CD4 T	6	5948660	5948694	chr19	-	RANBP3	NM_003624	Intron
6	MDM	6	99184661	99184692	chr4	+	RAP1GDS1	NM_001100429	Intron
6	MDM	6	134567527	134567617	chr9	-	RAPGEF1	NM_198679	Intron
6	CD4 T	6	130879473	130879498	chr5	+	RAPGEF6	NM_001164387	Intron
6	CD4 T	6	64502191	64502315	chr11	-	RASGRP2	NM_001098671	Intron
6	MDM	6	35706453	35706558	chr20	+	RBL1	NM_002895	Intron
6	MDM	1	66389264	66389356	chr11	+	RBM14	NM_006328	Intron
6	MDM	6	79917759	79917843	chr13	-	RBM26	NM_022118	Intron
6	CD4 T	6	34300922	34300944	chr20	+	RBM39	NM_004902	Undetermined
6	MDM	JR-FL	50000963	50000998	chr3	-	RBM6	NM_001167582	Intron
6	CD4 T	6	125653612	125653763	chr9	+	RC3H2	NM_001100588	Intron
6	MDM	6	66612880	66612968	chr11	-	RCE1	NM_005133	Intron
6	MDM	1	8760783	8761000	chr1	+	RERE	NM_001042681	Intron
6	MDM	6	33401982	33402038	chr17	-	RFFL	NR_037713	Intron
6	MDM	6	3231586	3231624	chr9	+	RFX3	NM_001282116	Intron
6	MDM	6	12919465	12919598	chr19	+	RNASEH2A	NM_006397	Intron
6	MDM	JR-FL	71683317	71683367	chr11	-	RNF121	NR_024148	Intron
6	MDM	JR-FL	179490809	179490843	chr5	+	RNF130	NM_018434	Intron
6	MDM	JR-FL	101908372	101908440	chr2	+	RNF149	NM_173647	Intron
6	MDM	6	74166727	74166774	chr17	-	RNF157	NM_052916	Intron
6	MDM	JR-FL	74545031	74545072	chr11	+	RNF169	NM_001098638	Intron
6	CD4 T	6	3956141	3956171	chr20	-	RNF24	NM_007219	Intron
6	MDM	6	55540562	55540583	chr8	-	RP1	NM_006269	Exon
6	MDM	6	1760768	1760919	chr17	+	RPA1	NM_002945	Intron
6	MDM	JR-FL	78579507	78579607	chr17	+	RPTOR	NM_001163034	Intron

6	CD4 T	6	78707419	78707513	chr17	-	RPTOR	NM_001163034	Intron
6	CD4 T	6	4132193	4132258	chr11	+	RRM1	NM_001033	Intron
6	CD4 T	6	123001527	123001644	chr12	+	RSRC2	NR_036435	Intron
6	MDM	6	63472080	63472159	chr11	-	RTN3	NM_006054	Intron
6	MDM	6	35500782	35500830	chr9	-	RUSC2	NR_052015	Intron
6	CD4 T	6	127833189	127833373	chr3	-	RUVBL1	NM_003707	Intron
6	MDM	JR-FL	5651572	5651615	chr19	-	SAFB	NM_002967	Intron
6	MDM	6	5620802	5620869	chr19	+	SAFB2	NM_014649	Intron
6	CD4 T	6	39844113	39844137	chr19	-	SAMD4B	NM_018028	Intron
6	MDM	6	109763113	109763222	chr1	+	SARS	NM_006513	Intron
6	MDM	6	53803929	53804149	chr4	-	SCFD2	NM_152540	Intron
6	MDM	6	100676803	100676937	chr12	-	SCYL2	NM_017988	Exon
6	MDM	JR-FL	75168974	75169046	chr17	-	SEC14L1	NM_001204408	Intron
6	MDM	6	80426176	80426242	chr7	+	SEMA3C	NM_006379	Intron
6	CD4 T	6	76371878	76372023	chr6	-	SENP6	NM_015571	Intron
6	MDM	6	47102340	47102436	chr3	+	SETD2	NM_014159	Intron
6	MDM	JR-FL	65824881	65824914	chr11	-	SF3B2	NM_006842	Intron
6	CD4 T	6	52355739	52355842	chr10	-	SGMS1	NM_147156	Intron
6	CD4 T	6	46620728	46620831	chr16	+	SHCBP1	NM_024745	Intron
6	MDM	JR-FL	38454724	38454789	chr19	-	SIPA1L3	NM_015073	Intron
6	MDM	JR-FL	38635110	38635185	chr19	+	SIPA1L3	NM_015073	Intron
6	CD4 T	6	46486056	46486254	chr17	+	SKAP1	NM_001075099	Intron
6	CD4 T	6	2230816	2230907	chr1	-	SKI	NM_003036	Intron
6	MDM	JR-FL	147377467	147377503	chr4	+	SLC10A7	NM_001029998	Intron
6	MDM	6	79236307	79236602	chr17	-	SLC38A10	NM_001037984	Intron
6	MDM	6	46594783	46594848	chrX	-	SLC9A7	NM_001257291	Intron
6	CD4 T	6	40844318	40844366	chr1	+	SMAP2	NM_022733	Intron
6	CD4 T	6	53436683	53436738	chrX	+	SMC1A	NM_006306	Intron
6	MDM	6	2727843	2727866	chr18	-	SMCHD1	NM_015295	Intron
6	CD4 T	6	55831672	55831721	chr2	+	SMEK2	NM_020463	Intron
6	MDM	6	1696055	1696098	chr17	-	SMYD4	NM_052928	Intron
6	MDM	6	43345939	43346048	chr3	-	SNRK	NM_017719	Intron
6	MDM	6	49593466	49593639	chr19	+	SNRNP70	NM_003089	Intron
6	MDM	6	53785556	53785641	chr12	-	SP1	NM_001251825	Intron
6	MDM	6	16221808	16221891	chr1	+	SPEN	NM_015001	Intron
6	MDM	6	54817392	54817486	chr2	+	SPTBN1	NM_003128	Intron
6	CD4 T	6	191965715	191965785	chr2	+	STAT4	NM_003151	Intron
6	CD4 T	6	43665137	43665259	chr7	+	STK17A	NM_004760	3´UTR
6	CD4 T	6	43631416	43631466	chr20	-	STK4	NM_006282	Intron
6	MDM	6	124118802	124118826	chr9	+	STOM	NR_073037	Intron
6	MDM	6	130404838	130404863	chr9	+	STXBP1	NM_001032221	Intron
6	CD4 T	6	172510503	172510637	chr1	+	SUCO	NM_016227	Intron
6	MDM	6	882095	882158	chr7	+	SUN1	NM_001171946	Intron
6	MDM	6	44945454	44945525	chr6	+	SUPT3H	NM_181356	Intron
6	MDM	6	50996447	50996573	chr22	+	SYCE3	NM_001123225	Intron
6	MDM	JR-FL	39759342	39759380	chr22	-	SYNGR1	NM_145731	Intron
6	MDM	1	16701586	16701756	chr1	-	SZRD1	NR_073503	Intron
6	MDM	6	27750595	27750653	chr17	+	TAOK1	NM_020791	Intron
6	MDM	JR-FL	27803667	27803749	chr17	-	TAOK1	NM_025142	Intron
6	MDM	JR-FL	29996037	29996111	chr16	+	TAOK2	NM_001252043	Intron
6	MDM	6	153641961	153642011	chrX	-	TAZ	NM_181312	Intron
6	CD4 T	6	38108864	38109038	chr4	+	TBC1D1	NM_015173	Intron
6	CD4 T	6	78320957	78321035	chr15	-	TBC1D2B	NM_144572	Intron
6	MDM	JR-FL	80762784	80762805	chr17	+	TBCD	NM_005993	Intron
6	MDM	JR-FL	52912384	52912457	chr18	+	TCF4	NM_001243236	Intron
6	MDM	6	62294212	62294335	chr17	-	TEX2	NM_001288733	Intron

6	MDM	JR-FL	153504966	153505012	chrX	-	TEX28	NM_001586	Intron
6	CD4 T	6	195785344	195785375	chr3	-	TFRC	NM_003234	Intron
6	CD4 T	6	82308216	82308307	chr9	+	TLE4	NM_001282753	Intron
6	CD4 T	6	172013255	172013292	chr2	+	TLK1	NM_012290	Intron
6	CD4 T	6	50142751	50142830	chr12	+	TMBIM6	NM_003217	Intron
6	CD4 T	6	124081043	124081179	chr12	-	TMED2	NM_006815	Intron
6	MDM	6	38663112	38663174	chr22	-	TMEM184B	NM_001195071	Intron
6	MDM	6	25777200	25777319	chr1	+	TMEM57	NM_018202	Intron
6	MDM	6	76064321	76064393	chr17	+	TNRC6C	NM_001142640	Intron
6	MDM	6	47516606	47516900	chr7	+	TNS3	NM_022748	Intron
6	MDM	6	47438161	47438260	chr7	+	TNS3	NM_022748	Intron
6	CD4 T	6	1303470	1303541	chr11	+	TOLLIP	NM_019009	Intron
6	MDM	6	144268134	144268165	chr7	+	TPK1	NM_022445	Intron
6	MDM	6	140089162	140089231	chr9	-	TPRN	NM_001128228	Intron
6	MDM	6	140090589	140090734	chr9	+	TPRN	NM_001128228	Intron
6	MDM	6	45503665	45503834	chr21	-	TRAPPC10	NM_003274	Intron
6	MDM	6	42307584	42307607	chr6	+	TRERF1	NM_033502	Intron
6	CD4 T	6	230697012	230697126	chr2	+	TRIP12	NM_001284215	Intron
6	MDM	6	49681229	49681284	chr19	-	TRPM4	NM_001195227	Intron
6	MDM	6	110268222	110268353	chr12	-	TRPV4	NM_021625	Intron
6	MDM	6	116736189	116736248	chr10	+	TRUB1	NM_139169	3´UTR
6	MDM	1	852868	852891	chr11	-	TSPAN4	NM_001025239	Intron
6	CD4 T	6	43476871	43476897	chr11	-	TTC17	NM_018259	Intron
6	MDM	6	99776207	99776319	chr15	+	TTC23	NM_001288615	Intron
6	MDM	6	20096537	20096573	chr2	+	TTC32	NM_001008237	3´UTR
6	CD4 T	6	94843163	94843318	chr5	-	TTC37	NM_014639	Intron
6	CD4 T	6	20766951	20767059	chr14	-	TTC5	NM_138376	Intron
6	MDM	JR-FL	52269897	52269920	chr3	+	TWF2	NM_007284	Intron
6	MDM	6	4217989	4218107	chr17	+	UBE2G1	NM_003342	Intron
6	MDM	6	4902828	4902919	chr16	-	UBN1	NM_001079514	Intron
6	MDM	6	4446828	4446919	chr19	-	UBXN6	NM_001171091	Intron
6	MDM	6	96991585	96991647	chr6	+	UFL1	NM_015323	Intron
6	MDM	6	62744201	62744264	chr12	+	USP15	NM_001252078	Intron
6	MDM	6	17180125	17180196	chr21	+	USP25	NM_001283042	Intron
6	MDM	6	219420168	219420196	chr2	+	USP37	NM_020935	Intron
6	MDM	6	234422638	234422728	chr2	+	USP40	NM_018218	Intron
6	MDM	6	144986190	144986215	chr6	-	UTRN	NM_007124	Intron
6	MDM	6	75705787	75705816	chr11	-	UVRAG	NM_003369	Intron
6	CD4 T	6	6851872	6852013	chr19	+	VAV1	NM_005428	Intron
6	MDM	6	51443102	51443214	chr3	+	VPRBP	NM_014703	Intron
6	CD4 T	6	51454157	51454240	chr3	+	VPRBP	NM_014703	Intron
6	MDM	6	150057733	150057826	chr1	-	VPS45	NM_007259	Intron
6	CD4 T	6	150085488	150085607	chr1	-	VPS45	NM_001279353	Intron
6	MDM	6	184650888	184650930	chr3	+	VPS8	NM_015303	Intron
6	MDM	6	102642114	102642401	chr14	-	WDR20	NM_001242418	Intron
6	CD4 T	6	118495976	118496285	chr1	-	WDR3	NM_006784	Intron
6	MDM	6	128506472	128506494	chr2	+	WDR33	NM_018383	Intron
6	MDM	6	36568010	36568057	chr19	+	WDR62	NM_173636	Intron
6	MDM	6	37706040	37706129	chr5	+	WDR70	NM_018034	Intron
6	CD4 T	6	9607492	9607533	chr11	-	WEE1	NM_003390	Intron
6	CD4 T	6	38192313	38192337	chr8	-	WHSC1L1	NM_023034	Intron
6	CD4 T	JR-FL	882726	882756	chr12	-	WNK1	NM_213655	Intron
6	CD4 T	6	898371	898511	chr12	-	WNK1	NM_213655	Intron
6	CD4 T	6	78832749	78832770	chr16	-	WWOX	NM_001291997	Intron
6	CD4 T	6	61749812	61749855	chr2	-	XPO1	NM_003400	Exon
6	MDM	6	28198382	28198492	chr16	-	XPO6	NM_001270940	Intron

6	MDM	JR-FL	64812782	64812872	chr12	-	XPOT	NM_007235	Exon
6	CD4 T	6	183442293	183442404	chr3	+	YEATS2	NM_018023	Intron
6	MDM	6	112858418	112858548	chr5	+	YTHDC2	NM_022828	Intron
6	CD4 T	6	1292584	1292623	chr17	+	YWHAE	NM_006761	Intron
6	MDM	6	9751250	9751322	chr2	+	YWHAQ	NM_006826	Intron
6	CD4 T	6	114117701	114117813	chr3	-	ZBTB20	NM_001164347	Intron
6	MDM	6	197146189	197146425	chr1	-	ZBTB41	NM_194314	Intron
6	MDM	1	113040138	113040392	chr2	-	ZC3H6	NM_198581	Intron
6	CD4 T	6	11873650	11873722	chr16	+	ZC3H7A	NM_014153	Intron
6	MDM	JR-FL	132338411	132338477	chr5	-	ZCCHC10	NM_017665	Intron
6	MDM	6	53011435	53011466	chr1	-	ZCCHC11	NM_015269	Intron
6	CD4 T	6	37343286	37343384	chr9	-	ZCCHC7	NR_110317	Intron
6	MDM	JR-FL	6623502	6623527	chr7	+	ZDHHC4	NM_001134388	Intron
6	MDM	6	133662456	133662538	chr12	-	ZNF140	NM_003440	Intron
6	MDM	6	3386159	3386203	chr11	+	ZNF195	NM_001130519	Intron
6	MDM	6	54030449	54030537	chr19	-	ZNF331	NM_018555	Intron
6	MDM	6	33750599	33750639	chr1	+	ZNF362	NM_152493	Intron
6	CD4 T	6	64192739	64192861	chr10	+	ZNF365	NM_199450	Intron
6	MDM	JR-FL	74392948	74392972	chr14	-	ZNF410	NM_021188	Intron
6	CD4 T	6	74378805	74378865	chr14	+	ZNF410	NM_021188	Intron
6	MDM	6	57012511	57012655	chr6	+	ZNF451	NM_015555	Undetermined
6	MDM	6	74199803	74200035	chr18	+	ZNF516	NM_014643	Intron
6	MDM	JR-FL	40505873	40505941	chr19	+	ZNF546	NM_178544	Intron
6	MDM	6	58924587	58924646	chr19	-	ZNF584	NM_173548	Intron
6	MDM	6	12260427	12260498	chr19	+	ZNF625-ZNF20	NR_037802	Intron
6	MDM	JR-FL	2939934	2940031	chr19	+	ZNF77	NM_021217	Intron
6	MDM	6	40592490	40592510	chr19	-	ZNF780A	NM_001142579	Intron
6	CD4 T	6	127026824	127026905	chr7	-	ZNF800	NM_176814	Intron
6	MDM	6	53461878	53461935	chr19	-	ZNF816-ZNF321P	NM_001202473	Intron
6	MDM	6	53158841	53158988	chr19	+	ZNF83	NM_001277946	Undetermined
6	CD4 T	6	30361783	30361806	chr7	+	ZNRF2	NM_147128	Intron
6	MDM	1	24637352	24637538	chr14	-			Intergenic
6	MDM	6	153323576	153323619	chr3	-			Intergenic
6	MDM	6	57800434	57800468	chr12	+			Intergenic
6	MDM	6	82338614	82338804	chr12	+			Intergenic
6	MDM	6	80718719	80718791	chr13	+			Intergenic
6	MDM	6	75460787	75460833	chr15	+			Intergenic
6	MDM	6	113117414	113117589	chr4	-			Intergenic
6	MDM	6	8407924	8408100	chr12	-			Intergenic
6	MDM	6	151808141	151808342	chr6	+			Intergenic
6	MDM	6	104281887	104282012	chr13	-			Intergenic
6	MDM	6	84021416	84021459	chr5	+			Intergenic
6	MDM	6	6937389	6937521	chr17	-			Intergenic
6	MDM	6	154953417	154953565	chr1	-			Intergenic
6	MDM	6	72387592	72387796	chr2	+			Intergenic
6	MDM	6	114853249	114853387	chr5	+			Intergenic
6	MDM	6	90375416	90375524	chr3	+			Intergenic
6	MDM	6	109399042	109399148	chr4	-			Intergenic
6	MDM	6	1539071	1539148	chr16	+			Intergenic
6	MDM	6	46635993	46636184	chr19	+			Intergenic
6	MDM	6	98358003	98358123	chr3	+			Intergenic
6	MDM	6	137782487	137782635	chr9	+			Intergenic
6	MDM	6	13853271	13853383	chr19	+			Intergenic
6	MDM	6	77382944	77383007	chr10	-			Intergenic
6	MDM	6	169890652	169890746	chr2	-			Intergenic

6	MDM	6	238166551	238166719	chr2	+	Intergenic
6	MDM	6	29557431	29557559	chr11	+	Intergenic
6	MDM	6	35689990	35690109	chr19	-	Intergenic
6	MDM	6	195679608	195679639	chr1	+	Intergenic
6	MDM	6	75829374	75829490	chr9	-	Intergenic
6	MDM	6	150586443	150586532	chrX	+	Intergenic
6	MDM	6	99481303	99481341	chr7	-	Intergenic
6	MDM	6	31538685	31538847	chr6	-	Intergenic
6	MDM	6	48391263	48391304	chr3	-	Intergenic
6	MDM	6	53001155	53001204	chr15	-	Intergenic
6	MDM	6	193585744	193585838	chr1	-	Intergenic
6	MDM	6	109528743	109528774	chr9	+	Intergenic
6	MDM	6	30141745	30141876	chr19	-	Intergenic
6	MDM	6	119748090	119748173	chr6	+	Intergenic
6	MDM	6	27802095	27802147	chr6	+	Intergenic
6	MDM	6	14337610	14337662	chr4	+	Intergenic
6	MDM	6	72278532	72278618	chr11	+	Intergenic
6	MDM	6	37122864	37122927	chr6	-	Intergenic
6	MDM	6	90265251	90265357	chr16	-	Intergenic
6	MDM	6	802031	802215	chr16	+	Intergenic
6	MDM	6	81553164	81553293	chr7	+	Intergenic
6	MDM	6	65319245	65319415	chrX	-	Intergenic
6	MDM	6	38042399	38042432	chr2	+	Intergenic
6	MDM	6	16751163	16751275	chr19	+	Intergenic
6	MDM	6	121571850	121571898	chr5	-	Intergenic
6	MDM	6	64209190	64209252	chr4	-	Intergenic
6	MDM	6	66445502	66445583	chr16	+	Intergenic
6	MDM	6	68990061	68990143	chr12	-	Intergenic
6	MDM	6	7777509	7777600	chr17	-	Intergenic
6	MDM	6	120103429	120103520	chr12	-	Intergenic
6	MDM	6	198418895	198418927	chr2	-	Intergenic
6	MDM	6	180251812	180251921	chr5	-	Intergenic
6	MDM	6	109699565	109699617	chr5	+	Intergenic
6	MDM	6	193974413	193974439	chr1	+	Intergenic
6	MDM	6	156367367	156367407	chr3	-	Intergenic
6	MDM	6	86136255	86136285	chr2	-	Intergenic
6	MDM	6	74829571	74829596	chr15	-	Intergenic
6	MDM	6	75773523	75773593	chr7	+	Intergenic
6	MDM	6	122093853	122093890	chr8	+	Intergenic
6	MDM	6	158296114	158296142	chr1	+	Intergenic
6	MDM	6	90686037	90686181	chr10	-	Intergenic
6	MDM	6	57316681	57316751	chr12	-	Intergenic
6	MDM	6	83210235	83210310	chr5	+	Intergenic
6	MDM	6	85197868	85197905	chr7	+	Intergenic
6	MDM	JR-FL	55184284	55184311	chr19	+	Intergenic
6	MDM	JR-FL	158703559	158703669	chr1	-	Intergenic
6	MDM	JR-FL	145989795	145989957	chr8	-	Intergenic
6	MDM	JR-FL	145996008	145996087	chr8	-	Intergenic
6	MDM	JR-FL	3768967	3769000	chr19	+	Intergenic
6	MDM	JR-FL	46429309	46429331	chr21	-	Intergenic
6	MDM	JR-FL	59982431	59982469	chr11	-	Intergenic
6	MDM	JR-FL	802765	802803	chr16	-	Intergenic
6	MDM	JR-FL	31441181	31441215	chr20	-	Intergenic
6	MDM	JR-FL	42855856	42855902	chr14	+	Intergenic
6	MDM	JR-FL	23669493	23669521	chr14	+	Intergenic
6	MDM	JR-FL	160858989	160859039	chr1	-	Intergenic

6	MDM	JR-FL	194641110	194641190	chr3	-			Intergenic
6	MDM	JR-FL	26137569	26137602	chr6	+			Intergenic
6	MDM	JR-FL	187749	187776	chr19	+			Intergenic
6	MDM	JR-FL	65710293	65710345	chr11	-			Intergenic
6	MDM	JR-FL	71827044	71827079	chr14	-			Intergenic
6	MDM	JR-FL	248786925	248787011	chr1	-			Intergenic
6	MDM	JR-FL	39455770	39455792	chr22	-			Intergenic
6	CD4 T	6	110339291	110339316	chr1	-			Intergenic
6	CD4 T	6	118966646	118966684	chr11	-			Intergenic
6	CD4 T	6	32807715	32807743	chr6	-			Intergenic
6	CD4 T	6	34778008	34778038	chr15	+			Intergenic
6	CD4 T	6	68767510	68767595	chr13	-			Intergenic
6	CD4 T	6	18728351	18728533	chr5	-			Intergenic
6	CD4 T	6	17650753	17650892	chr7	-			Intergenic
6	CD4 T	6	129292850	129292948	chr8	+			Intergenic
6	CD4 T	6	59631654	59631783	chr8	+			Intergenic
6	CD4 T	6	7780477	7780554	chr17	+			Intergenic
6	CD4 T	6	31775857	31775880	chr6	+			Intergenic
6	CD4 T	6	203590683	203590779	chr1	-			Intergenic
6	CD4 T	6	117323269	117323290	chr1	+			Intergenic
6	CD4 T	6	32574161	32574266	chr6	-			Intergenic
6	CD4 T	6	9000652	9000787	chr1	-			Intergenic
6	CD4 T	6	25882549	25882574	chr4	+			Intergenic
6	CD4 T	6	62516864	62516946	chr11	-			Intergenic
6	CD4 T	6	10966611	10966668	chr16	+			Intergenic
6	CD4 T	6	64553339	64553451	chr11	+			Intergenic
6	CD4 T	6	37516061	37516099	chr22	-			Intergenic
6	CD4 T	6	109735568	109735591	chr11	+			Intergenic
6	CD4 T	6	65521889	65521912	chr13	-			Intergenic
6	CD4 T	6	36198591	36198680	chr19	-			Intergenic
7	CD4 T	7	60563121	60563174	chr13	+	DIAPH3	NM_001258367	Intron
7	CD4 T	7	94758083	94758197	chr10	-	EXOC6	NM_001013848	Intron
7	CD4 T	7	179075552	179075695	chr3	-	MFN1	NM_033540	Intron
7	CD4 T	7	219364280	219364300	chr2	+	USP37	NM_020935	Intron
7	CD4 T	7	75283538	75283588	chr14	+	YLPM1	NM_019589	Intron
